# Greenhouse Gas Mitigation Potential of Temperate Fen Paludicultures

**DOI:** 10.1111/gcb.70385

**Published:** 2025-07-30

**Authors:** Carla Bockermann, Tim Eickenscheidt, Matthias Drösler

**Affiliations:** ^1^ Weihenstephan‐Triesdorf University of Applied Sciences, Peatland Science Centre (PSC) Freising Germany; ^2^ Technical University of Munich, TUM School of Life Sciences Freising Germany

**Keywords:** carbon balance, *Carex*, LULUCF, organic soil, peatland, *Phalaris*, *Phragmites*, preliminary emission factor, *Typha*, water table

## Abstract

Peatlands lose their valuable carbon (C) sink function under intensive land use and turn into greenhouse gas (GHG) emission hotspots. Despite scarce empirical evidence, paludiculture is expected to have significant GHG mitigation potential for organic soils. This study provides the first comprehensive dataset on full GHG balances for newly established fen paludicultures over a water table (WT) gradient spanning an annual mean WT of −0.29 to +0.04 m, stratified into moderately rewetted conditions (−0.30 < WT < −0.10 m) and rewetted conditions (WT ≥ −0.10 m). We used manual and novel automated chambers to measure annual carbon dioxide (CO_2_), methane, and nitrous oxide emissions from five typical fen plant species ( *Carex acutiformis* , *Phalaris arundinacea* , *Phragmites australis* , *Typha angustifolia* , and *Typha latifolia* ) newly established as peatland biomass crops in three temperate fen peatlands in southern Germany. Our study confirms a significant GHG mitigation potential for the tested plant species and found a C sink function of paludiculture. The results yield preliminary emission factors of −0.1 and −12.0 t CO_2_‐equivalents ha^−1^ year^−1^ under moderately rewetted conditions (*n* = 39) and under rewetted conditions (*n* = 43), respectively. We further identify an optimal annual mean WT of −0.07 m for maximizing GHG reduction across all plant species and sites with a net C sink achieved at a mean annual WT of ≥ −0.12 m. Presuming the conversion of arable land into paludiculture, a mitigation potential of up to −51.9 t CO_2_‐equivalents is attainable per hectare and year. These findings highlight that well‐managed paludiculture could make a considerable contribution toward achieving the politically targeted CO_2_ sink function in the LULUCF sector.

## Graphical abstract

Peatlands lose their valuable carbon sink function under intensive land use and turn into greenhouse gas (GHG) emission hotspots. This study presents the first full GHG balance dataset for five potential fen paludicultures, measuring CO_2_, methane, and nitrous oxide emissions using manual and novel automated chambers across a water table gradient in southern Germany. Results show paludiculture can restore the carbon sink function and significantly mitigate GHG emissions, especially at an optimal mean water table of −0.07 m. Rewetted conditions offer mitigation of up to −51.9 t CO_2_‐equivalents per hectare annually, supporting paludiculture's role in climate targets.

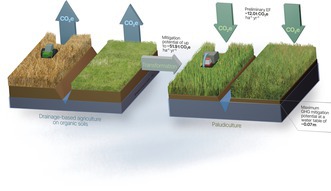

## 1 Introduction

Peatlands can be part of the climate problem or become part of the solution—depending on how we decide to use them. Disturbances like drainage and intensive use degrade peat carbon (C), weaken valuable ecosystem functions, and create hot spots of greenhouse gas (GHG) emissions (e.g., Drösler et al. 2008; IPCC 2014; Freeman et al. 2022; UNEP 2022). In Europe, most of the 58.6 million ha of peatlands are found in the temperate and boreal regions, in which approximately 46% of these peatlands are considered degraded (UNEP 2022). Consequently, drainage‐based land use in Russia, Belarus, Germany, Finland, Poland, Great Britain, and Ireland causes emissions of 582 Mt carbon dioxide (CO_2_)‐equivalents (CO_2_e) year^−1^ (UNEP 2022). These peatland emissions, contributing to around 25% of the total European agricultural emissions (Tanneberger et al. 2020), are reported in the sectors Agriculture (direct nitrous oxide (N_2_O) emissions) and Land Use, Land Use Change and Forestry (LULUCF) (CO_2_ and methane (CH_4_) emissions) in annual national inventory reports (NIR) under the UNFCCC. In Germany, decades of intensive agriculture and forestry land use have led to the degradation of about 92% of the 1.93 million ha of peatland soils (5.1% of total land area, 7% of the total agricultural area) (Tiemeyer et al. 2020; Wittnebel et al. 2023). The repercussions of Germany's intensive land use have caused substantial GHG emissions amounting to 53.3 Mt CO_2_e or 7.1% of the total national budget in 2022, with ca. 80% originating from drained cropland and grassland (UBA 2024). Despite Germany's efforts to comply with the Paris Agreement and associated European regulations to reduce national GHG emissions by 65% (compared with 1990) by 2030, Germany is not on track to meet this target (Federal Climate Change Act 2019, amended 2021). Reaching this goal entails cutting the agricultural sector's emission budget by 14 Mt CO_2_e annually between 2020 and 2030 and enhancing the sink function of the LULUCF sector to −25 Mt CO_2_e by 2030 (Federal Climate Change Act 2019, amended 2021). Considering their disproportionately high emission rates, organic soils present a sensible and timely target for national GHG reduction efforts. Accordingly, Germany aims to reduce five of the 14 Mt CO_2_e through more sustainable peatland use (National Peatland Protection Strategy, BMUV 2022).

The primary measure to mitigate the large amounts of GHG emissions from degraded organic soils is rewetting (e.g., IPCC 2014; Wilson et al. 2016, 2022; Bianchi et al. 2021; Evans et al. 2021). Rewetting the soil reinstates anaerobic conditions, arresting aerobic microbial mineralization of soil C and the resulting release of CO_2_, which constitutes the majority of total GHG emissions from drained organic soils (e.g., Tiemeyer et al. 2016, 2020; Freeman et al. 2022). Water tables (WT) shallower than −0.1 m below the surface define naturally wet or rewetted soils (IPCC 2014; Tiemeyer et al. 2020) and present the most reliable estimate for greatest mitigation effects (Drösler et al. 2008; Evans et al. 2021). Restoration is a further effective mitigation approach that has contributed to revitalizing ecosystem services of degraded organic soils in Germany since the 1990s. However, the associated land loss has caused a lack of economic acceptance, which continues to impede large‐scale implementation (e.g., Buschmann et al. 2020). While restoration will regain significance through the 2024 EU Nature Restoration Law, restoration and rewetting are often loosely defined, measures may not always recover anticipated ecosystem services, and they often entail small net emissions. This can be attributed to increased CH_4_ emissions from unintended WT fluctuations or flooded soils (i.e., imprecise water management) as reflected in the proposed national EF of 5.5 t CO_2_e ha^−1^ year^−1^ for ‘rewetted organic soils’ (Tiemeyer et al. 2020). To reconcile the seemingly contradictory demands on land use, restoration and mitigation, the cultivation of biomass on wet soils (paludiculture) presents a promising nature‐based climate solution that is integrating sustainable land use with peat soil preservation and enhancing ecological services (IPCC 2014; UNEP 2022). Plant species highly suitable for paludiculture with promising biomass yield and utilization potentials include common fen species such as sedges (*Carex* spp.), reed canary grass ( *Phalaris arundinacea* L.), common reed ( *Phragmites australis* (Cav.) Trin. ex Steud.), cattail spp. (*Typha* spp.), and bog species *Sphagnum* spp. (cf. Abel et al. 2013; Hartung et al. 2020, 2023; Kuptz et al. 2022). While complete GHG balances for *Sphagnum* paludiculture in bogs are empirically established to be a weak net source (e.g., Oestmann et al. 2022; Daun et al. 2023), GHG values for managed fen paludicultures differ widely between the few empirical field studies and are estimated at 0 to 18 t CO_2_e ha^−1^ year^−1^ (e.g., Geurts and Fritz 2018; Bianchi et al. 2021; Närmann and Tanneberger 2021; Tanneberger et al. 2022). For simulated or estimated harvests in natural or rewetted fens with potential paludiculture vegetation, direct flux measurements resulted in 0.4–31.1 t CO_2_e ha^−1^ year^−1^ for *Carex* stands, 3.7–17.3 t CO_2_e ha^−1^ year^−1^ for *Phragmites*, and −1.4–13.1 t CO_2_e ha^−1^ year^−1^ for *Typha* spp. (Günther et al. 2015; Van Den Berg et al. 2024). Presently, the only studies providing complete GHG balances from direct flux measurements in newly established paludicultures are Kandel et al. (2020) reporting 10.4 to 36.4 t CO_2_e ha^−1^ year^−1^ in fertilized *Phalaris*, and Bockermann et al. (2024), reporting a sink of −11.1 t CO_2_e ha^−1^ year^−1^ in a *Carex* paludiculture. Hence, the concept is vastly growing in policy, scenario modelling, and implementation strategies as studies conclude strong mitigation potentials. However, a comprehensive empirical basis under appropriate rewetting scenarios and different management intensities is lacking to give coherent GHG values for paludicultures and adequate representation in EFs for accurate representation in climate models or eco‐schemes.

The objectives of this field study were to (1) assess the GHG mitigation potential of newly established fen paludiculture plant species across gradients of WTs, management intensities, and site conditions, (2) identify the most significant key drivers influencing the GHG mitigation potential of fen paludicultures, (3) determine the optimal water level for maximizing GHG mitigation across all tested paludicultures, with the goal of optimizing implementation strategies and WT management practices in fen paludicultures, (4) empirically verify the minimum required water level as defined by the present paludiculture definition, and (5) derive preliminary EFs for paludicultures, disaggregated by WT levels, using measured GHG balances to facilitate potential integration into emission reporting frameworks.

The following reported comprehensive dataset provides a novel and robust argument for immediate action to improve peatland management as a key mitigation solution for the agricultural and LULUCF sectors.

## 2 Methods

### 2.1 Site Description

We conducted the field experiments at three fen peatlands in southeastern Germany, representing different typical drainage‐based land‐use histories and degradation states (see Table S1). The study region has a humid continental climate that is transitioning to a temperate oceanic climate due to climate change (Köppen‐Geiger climate classification, Beck et al. 2023). The FSM site lies within the “Freisinger Moos,” a percolation fen located at the northern edge of the Munich gravel plain, about 30 km north of Munich, Germany (experimental site 1.0 ha, location 48°22′43.6″ N 11°41′01.6″ E, elevation 446 m a.m.s.l.). The FSM site was originally drained and intensively managed as grassland with an average ground WT of approximately −0.4 m. The FSM site consists of two plots: a field‐scale plot (FSM‐F for field‐scale) within the rewetted part of a paludiculture plant establishment trial area and an adjacent experimental plot (FSM‐E for experimental‐scale) with a basin structure for precise water management.

The LM site (field‐scale site 5.0 ha, location 48°37′32.9″ N 11°12′54.2″ E, elevation 384 m a.m.s.l.) lies within the “Old Bavarian Donaumoos,” the largest lowland fen in southern Germany. This site was originally drained and intensively managed as arable land with an average WT of approximately −0.7 m.

The RH site (field‐scale site 1.2 ha, location 48°29′54.0″ N 10°15′09.8″ E, elevation 442 m a.m.s.l.) lies within the “Swabian Donaumoos,” a lowland fen shared between the states of Bavaria and Baden‐Württemberg. This site was originally drained and intensively managed as arable land and grassland with an average WT below −1.0 m. Site climate information is provided in Table 1 and methods for soil property analyses are given in Data S1.

**TABLE 1:** Site meteorological data for the study sites Freisinger Moos (FSM), Langenmosen (LM), and Riedhausen (RH) in the relevant measurement years 2019–2021 and compared with multi‐year means from nearby climate stations.

Parameter	FSM	LM	RH
Air temperature 2 m, °C			
2019	9.8		
2020	9.7		
2021	8.6	8.9	8.5
*Multi‐year mean (1991–2020)* ^a^	*8.9*	*9.2*	*9.2*
Precipitation annual sum, mm m^−2^ year^−1^ ^a^			
2019	648.0		
2020	762.2		
2021	896.4	861.7	804.2
*Multi‐year mean (1991–2020)*	*757.1*	*732.3*	*716.2*
Sum of global radiation, Wh m^−2^ year^−1^ ^b^			
2019	1230.3		
2020	1248.2		
2021	1230.1	1148.8	1283.1
Amount of vegetation days^b^			
2019	257		
2020	254		
2021	234	237	227

^a^ DWD [German Weather Service] stations Munich airport, Neuburg/Donau, and Günzburg.

^b^ Bayerische Landesanstalt für Landwirtschaft [Bavarian State Office for Agriculture], Agrarmeteorologie Bayern, www.wetter‐by.de.

### 2.2 Investigated Plant Species and Paludiculture Management

Establishment of paludicultures occurred between 2016 and 2020 by planting or sowing and subsequent rewetting (Eickenscheidt et al. 2023) to quantify GHG fluxes 1 to 5 years after paludiculture crop establishment. The paludicultures include the five fen plant species lesser pond sedge ( *Carex acutiformis* Ehrh., hereafter *Carex*), reed canary grass ( *Phalaris arundinacea* L., *Phalaris*), common reed ( *Phragmites australis* (Cav.) Trin. ex Steud., *Phragmites*), narrowleaf cattail ( *Typha angustifolia* L., *T. angustifolia*), and broadleaf cattail ( *Typha latifolia* L., *T. latifolia*). Prior to plant establishment, the sites were mechanically prepared by reverse rotary tilling to a depth of 10–13 cm in FSM and by conventional plowing to a depth of 20–25 cm in LM and RH, followed by rotary cultivation with a roller to reconsolidate the soil. No topsoil was displaced. Weeds were removed from the established *T. latifolia* stands at site FSM in the year preceding the measurements. All other frames exhibited minimal weed presence and consisted almost entirely of the target paludiculture species. Paludiculture management included an annual winter cut (Dec to Feb) starting the year of plant establishment. The harvested biomass was oven‐dried at 60°C for 48 h for dry matter (DM) yield quantification and for organic C (Corg) analyses via dry combustion (AGROLAB GmbH, Germany). In 2021, an additional early summer harvest was conducted to test the effects of a two‐cut regime in the RCG paludicultures at LM (June harvest) and at FSM‐E (July harvest). To assess further effects of management intensity, we subsequently applied liquid biogas digestate for balanced fertilization at FSM‐E (N application rates: 92, 122, and 143 kg N ha^−1^ for basins 1, 2, and 3, respectively). Detailed information on experimental setup and rewetting measures is provided in S2. Table 2 details all implemented paludiculture treatments in this study (*n* = 33).

**TABLE 2:** Overview of tested paludiculture treatments (with three replicates each), including plant species and management‐specific information for the field‐scale sites Freisinger Moos (FSM‐F), Langenmosen (LM), and Riedhausen (RH) in 2019–2021, and at the experimental basin site at Freisinger Moos (FSM‐E).

Site	Plant species	Target species cover and accompanying species^a^, %	Y.o.e	Y.o.m	Method	WT class	Mean annual, WT, m	Organic fertilizer, N application, kg N ha^−1^	Harvest t.o.y	Mean yield ± SD, t DM ha^−1^ year^−1^
FSM‐F	*Carex acutiformis*	98%–100%	2016	2019	Manual	Rewetted	−0.07		December	7.6 ± 0.9
FSM‐F	*Phalaris arundinacea*	92%–99%; *Carex acuta* 0%–8%	2016	2019	Manual	Rewetted	−0.07		December	7.0 ± 2.9
FSM‐F	*Phragmites australis*	90%–97%; *Carex acuta* 0%–4%; *Lolium perenne* 0%–3%	2016	2019	Manual	Rewetted	−0.04		December	4.1 ± 0.3
FSM‐F	*Typha latifolia*	85%–95%; *Brachypodium pinnatum* 3%–4%; *Lolium perenne* 0%–4%	2016	2019	Manual	Rewetted	−0.09		December	3.2 ± 0.5
LM	*Carex acutiformis*	95%–99%; *Poa pratensis* 1%	2019	2021	Manual	Rewetted	−0.04		December	11.5 ± 2.9
LM	*Phalaris arundinacea*	95%–99%; *Poa pratensis* 0%–4%	2019	2021	Manual	Rewetted	0.00		July + December	15.0 ± 2.0
LM	*Typha angustifolia*	85%–95%; *Elymus repens* 2%; *Equisetum palustre* 2%	2018	2021	Manual	Rewetted	0.00		December	9.7 ± 1.5
RH	*Carex acutiformis*	95%–99%; *Poa pratensis* 1%–4%; *Equisetum arvense* 1%–2%	2019	2021	Manual	Moderately rewetted	−0.29		December	10.0 ± 1.7
RH	*Phalaris arundinacea*	95%–99%; *Elymus repens* 2%–4%; *Equisetum palustre* 1%–2%	2020	2021	Manual	Moderately rewetted	−0.13		December	2.9 ± 0.2
FSM‐E	*Carex acutiformis*	Not surveyed	2017	2020	ARC	Rewetted	−0.03		December	10.3 ± 0.8
FSM‐E	*Carex acutiformis*	Not surveyed	2017	2020	ARC	Moderately rewetted	−0.12		December	9.3 ± 1.7
FSM‐E	*Carex acutiformis*	Not surveyed	2017	2020	ARC	Moderately rewetted	−0.20		December	6.2 ± 0.7
FSM‐E	*Phalaris arundinacea*	Not surveyed	2017	2020	ARC	Rewetted	−0.04		December	5.7 ± 0.6
FSM‐E	*Phalaris arundinacea*	Not surveyed	2017	2020	ARC	Moderately rewetted	−0.12		December	7.0 ± 0.6
FSM‐E	*Phalaris arundinacea*	Not surveyed	2017	2020	ARC	Moderately rewetted	−0.22		December	5.1 ± 0.6
FSM‐E	*Phragmites australis*	Not surveyed	2017	2020	ARC	Rewetted	−0.03		December	6.6 ± 0.8
FSM‐E	*Phragmites australis*	Not surveyed	2017	2020	ARC	Moderately rewetted	−0.13		December	6.4 ± 0.3
FSM‐E	*Phragmites australis*	Not surveyed	2017	2020	ARC	Moderately rewetted	−0.22		December	3.9 ± 1.1
FSM‐E	*Typha latifolia*	Not surveyed	2017	2020	ARC	Rewetted	0.00		December	7.9 ± 1.2
FSM‐E	*Typha latifolia*	Not surveyed	2017	2020	ARC	Moderately rewetted	−0.12		December	5.7 ± 1.7
FSM‐E	*Typha latifolia*	Not surveyed	2017	2020	ARC	Moderately rewetted	−0.22		December	4.3 ± 0.3
FSM‐E	*Carex acutiformis*	Not surveyed	2017	2021	ARC	Rewetted	−0.02		December	9.3 ± 0.4
FSM‐E	*Carex acutiformis*	Not surveyed	2017	2021	ARC	Rewetted	−0.10		December	7.6 ± 1.5
FSM‐E	*Carex acutiformis*	Not surveyed	2017	2021	ARC	Moderately rewetted	−0.14		December	4.3 ± 0.8
FSM‐E	*Phalaris arundinacea*	Not surveyed	2017	2021	ARC	Rewetted	0.01	92.0	July + December	12.4 ± 2.2
FSM‐E	*Phalaris arundinacea*	Not surveyed	2017	2021	ARC	Rewetted	−0.10	122.0	July + December	10.8 ± 1.9
FSM‐E	*Phalaris arundinacea*	Not surveyed	2017	2021	ARC	Moderately rewetted	−0.15	143.0	July + December	8.2 ± 1.6
FSM‐E	*Phragmites australis*	Not surveyed	2017	2021	ARC	Rewetted	0.01		December	11.9 ± 0.8
FSM‐E	*Phragmites australis*	Not surveyed	2017	2021	ARC	Rewetted	−0.09		December	9.97 ± 0.6
FSM‐E	*Phragmites australis*	Not surveyed	2017	2021	ARC	Moderately rewetted	−0.18		December	1.6 ± 0.8
FSM‐E	*Typha latifolia*	Not surveyed	2017	2021	ARC	Rewetted	0.04		December	8.8 ± 0.8
FSM‐E	*Typha latifolia*	Not surveyed	2017	2021	ARC	Rewetted	−0.10		December	6.2 ± 0.5
FSM‐E	*Typha latifolia*	Not surveyed	2017	2021	ARC	Moderately rewetted	−0.16		December	1.8 ± 0.3
FSM^b^	*Carex acutiformis*	> 90%	2016	2017	Manual	Moderately rewetted	−0.13		September	14.3 ± 1.0
FSM^b^, ^c^	*Carex acutiformis*	> 90%	2016	2017	Manual	Moderately drained	−0.39		September	13.9 ± 0.7

Abbreviations: harvest t.o.y, harvest time of year; method, chamber measurement method; Y.o.e, year of establishment; y.o.m, year of measurement.

^a^ Up to two most abundant accompanying species.

^b^ Bockermann et al. (2024).

^c^ Not included in paludiculture EF calculations due to WT threshold.

### 2.3 Greenhouse Gas Flux Measurements

Flux measurements of the GHG CO_2_, CH_4_, and N_2_O to capture annual GHG exchange were determined with manual chambers between 02 Jan 2019 and 09 Jan 2020 at FSM‐F, and between 17 Dec 2020 and 14 Jan 2022 at LM and RH. Fluxes at site FSM‐E were conducted with a fully automatic robotic chamber measurement system (ARC) between 02 Jan 2020 and 31 Dec 2021. Manual measurements of CO_2_ were carried out three‐weekly as full‐day campaigns, and measurements of CH_4_ and N_2_O fluxes were conducted in seven‐day intervals. ARC measurements of CO_2_ (NEE and Reco) and combined measurements of CH_4_ and N_2_O alternated on a quasi‐daily basis, enabling high temporal resolution of fluxes while minimizing site disturbance. For chamber GHG flux measurements, we permanently installed soil collars (PVC) in clusters of three spatial replicates per paludiculture treatment covering an area of 0.5625 m^2^ per treatment replicate for manual measurements and 1.0 m^2^ for ARC GHG exchange detection. We modeled CO_2_ fluxes (Reco and GPP) using a campaign‐ and treatment‐based approach for manually determined fluxes (e.g., Eickenscheidt et al. 2015; Tiemeyer et al. 2024). For ARC, the increased measurement frequency and expanded spatial coverage, along with the weekly aggregation of fluxes, allowed the modeling of fluxes per replicate. A detailed description of manual and automated GHG measurements, GHG flux calculations, and CO_2_ modelling, including metrics for model validation and uncertainty estimation, is provided in S3, Figure S4, and Table S2. A list of campaign‐specific model parameters is given in Repository Table R1 (R1.1 Reco and R1.2 GPP) Bockermann et al. (2025).

### 2.4 Calculation of Net Ecosystem Carbon Balance, Total Greenhouse Gas Balances, and Emission Factors

We calculated the net ecosystem carbon balance (NECB) in t C ha^−1^ year^−1^ from the total C loss via emissions of NEE CO_2_ and CH_4_, and including C imports determined from the applied fertilizer and C exports from the dried harvest biomass (Equation 1):

(1) $$\text{NECB}\left[t\;C\;{\mathrm{ha}}^{-1}{\text{year}}^{-1}\right]=\mathrm{NEE}‐C-C\;\text{import}+C\;\text{export}+{\mathrm{CH}}_{4}-C$$

We calculated the net climate effects from the total GHG emissions in CO_2_‐equivalents (CO_2_e), including C imports and exports. We used the 100‐year global warming potentials (GWP100) of 28 for CH_4_ and 265 for N_2_O, as provided in the IPCC Fifth Assessment Report (Myhre et al. 2013), consistent with the current German national GHG inventory reporting. For comparison with the current EFs for organic soil land‐use categories (Tiemeyer et al. 2020) we reported values using IPCC AR4 GWP100 (25 for CH_4_, 298 for N_2_O; Forster et al. 2007). We give uncertainties of the GHG exchange means as the 95th percentiles. Fertilizer C import and harvest C export were converted from Corg to CO_2_e (IPCC 2014) and included in the total balances (Equation 2):

(2) $$\begin{array}{c} \mathrm{GHG}\;\text{balance}\;\left[t\;{\mathrm{CO}}_{2}e\;{\mathrm{ha}}^{-1}{\text{year}}^{-1}\right]=\mathrm{NEE}\\ \;-3.66*C\;\text{import}+3.66*\text{Cexport}+28*{\mathrm{CH}}_{4}+265*N_{2}O \end{array}$$

Imported DOC from irrigation water and DOC losses were not quantified.

For calculation of emission factors (EF) and for model development, we extended the dataset by including annual balances of two *C. acutiformis* treatments from the site FSM (see Table 2). Treatments were measured manually in 2017 and quantified using the same methodological approach as described in Bockermann et al. (2024). The final CO_2_ and GHG balance datasets included 83 annual balances from 11 manually measured treatments (with three replicates each) and 72‐ARC measured replicates (from 12 treatments × 3 replicates × 2 years). The final CH_4_ and N_2_O datasets included 105 replicate‐specific annual balances each from the 33 manual and 72 ARC replicates.

We calculated a preliminary EF for fen paludiculture on wet organic soils (‘rewetted fen paludiculture,’ mean annual WT ≥ −0.1 m) in accordance with the current German GHG inventory reporting for organic soils (Tiemeyer et al. 2020) and the current IPCC definition of ‘rewetted organic soils’ (IPCC 2014). Further, we suggest a distinction for fen paludiculture on moderately wet organic soils (‘moderately rewetted fen paludiculture’) where mean annual WTs are shallower than −0.3 m. This threshold is considered a proxy for rewetted (naturally wet) or ‘shallow‐drained’ organic soils by the IPCC Wetlands Supplement (IPCC 2014). For comparability, we included IPCC default EFs for DOC C export (drained: CO_2_–CDOC = 0.31 t C ha^−1^ year^−1^; rewetted: CO_2_–CDOC = 0.24 t C ha^−1^ year^−1^) and for ditch CH_4_ emissions (drained: EF 527 kg CH_4_ ha^−1^ year^−1^ multiplied by the German default ditch fraction 0.013; rewetted: not applicable) (IPCC 2014; Tiemeyer et al. 2020). In our dataset of *n* = 83 balances, 43 balances were considered ‘rewetted fen paludiculture’ and 39 balances ‘moderately rewetted fen paludiculture’. One balance with a mean annual WT of −0.39 m was excluded for EF derivation. We follow the atmospheric sign convention, where positive fluxes and budgets indicate a release of C and GHGs from the ecosystem into the atmosphere.

### 2.5 Statistical Analyses

All statistical analyses were performed using the statistical software R (version 4.4.2, R Core Team 2024). For data exploration, we followed the eight‐step protocol described by Zuur et al. (2010), which comprises the following assessments: detection of outliers in the response (*Y*) and explanatory variables (*X*); evaluation of homogeneity and normality in *Y*; assessment of zero‐inflation in *Y*; examination of collinearity among *X* variables; exploration of relationships between *Y* and *X*; identification of potential interactions; and evaluation of independence in *Y*. To investigate the observed nonlinear responses of annual CH_4_ exchange, annual N_2_O exchange, or annual GHG balances to ecological or management‐related drivers, we used generalized linear mixed effects models (GLMMs for CH_4_ and N_2_O) (R package “glmmTMB” v1.1.10, Brooks et al. 2024) or generalized additive mixed effects models (GAMMs for GHG balances) (R package “mgcv” v1.9.1, Wood 2023). The CH_4_, N_2_O, or GHG balances at each measurement site are not independent of each other; therefore, we included the covariate “Site” as a random effect in each GLMM or GAMM. For each response variable, the model formulation began with a saturated model followed by backward selection through dropping non‐significant terms one at a time based on *p*‐values of an applied likelihood ratio test. The final formula for the annual CH_4_ exchange was CH_4_~WT + plant genus + plant biomass + (1|Site) (*R* ^2^marg. = 0.88, *R* ^2^cond. = 0.90, ICC = 0.12). Since CH_4_ data were positively skewed, we used a Tweedie distribution with a log link function for the conditional mean to avoid unrealistic high annual CH_4_ uptakes and to account for the nonlinear response of CH_4_ to WT. For the annual N_2_O exchange, the final formula was N_2_O~WT + plant genus + stand year after establishment + (1|Site) (*R* ^2^marg. = 0.40, *R* ^2^cond. = 0.40, ICC = 0.00). For N_2_O, we used a Gaussian distribution with a square root link function to account for the nonlinear response of N_2_O to WT and the positively skewed N_2_O data. For annual NECBs or GHG balances, the final formula was NECB or GHG balance~s(WT, bs = “cr”) + plant genus + management intensity, random = list(Site = ~1) (NECB: *R* ^2^adj. = 0.58, ICC = 0.67; GHG balance: *R* ^2^adj. = 0.52, ICC = 0.63). Model validation was performed following Zuur and Ieno (2014, 2021, 2024), using (i) scatterplots of the residuals versus fitted values, versus each covariate in the model and those not in the model, (ii) inspection of the random intercepts, and (iii) model validation options (e.g., Q–Q plot residuals, nonparametric dispersion test) from the R package “DHARMa” (v0.4.7, Hartig et al. 2024). As the final step of model validation, we used the R package “ggeffect” (v2.0.0; Lüdecke et al. 2024) to assess the reliability of the model predictions.

## 3 Results

### 3.1 Site Conditions and Biomass Development

#### 3.1.1 Site Conditions

Regional weather conditions were slightly warmer and drier in 2019, slightly warmer with equal precipitation in 2020, and slightly cooler and wetter in 2021 compared to the multi‐year mean (Table 1). We classified the soils at both sites, FSM and LM, as *Sapric Histosols* according to the IUSS Working Group WRB (2022), corresponding to “Erdniedermoor” in the German soil classification system (KA6; AG Boden 2024). At FSM, the peat layer ranged from 2.55 to 2.90 m, and at LM from 2.30 to 3.65 m, both consisting predominantly of “radicels” sedge peat with varying admixtures of alder and *Phragmites* peat. The upper peat layers were earthified: 0.34–0.40 m at FSM and 0.37–0.54 m at LM, composed of amorphous, unstructured peats with high decomposition degrees (von Post H8–H9). The soil at site RH was classified as a Mollic Gleysol or “Moorfolgeboden” (peat‐derived organic soil) with a 0.26–0.65 m peat layer. The upper 0.26–0.58 m was strongly earthified peat and exhibited advanced decomposition (von Post H9–H10). Further classification and chemical and physical peat properties are given in S1, Table S1 and Figure S1.

#### 3.1.2 Water Management

**FIGURE 1:**
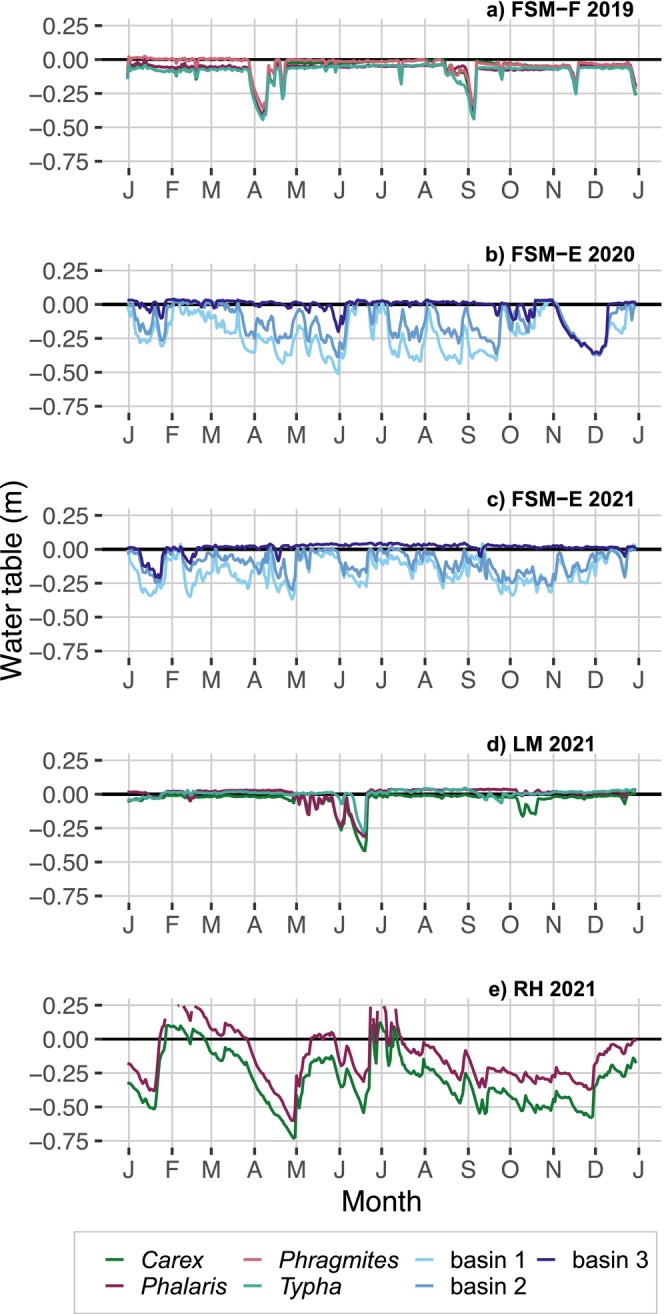
Water table (WT) levels (m) during the greenhouse gas measurement periods 2019–2021 in the experimental basin site at Freisinger Moos (b) FSM‐E 2020 and c) FSM‐E 2021) differentiated by the three WT class basins, and in the field‐scale sites a) Freisinger Moos (FSM‐F 2019), d) Langenmosen (LM 2021), and e) Riedhausen (RH 2021) differentiated by paludiculture plant. WT values are interpolated from 0.5‐h logged data.

WT dynamics in the FSM‐F treatments were decoupled from precipitation due to successful rewetting measures, resulting in mean annual WTs of −0.04 to −0.09 m with virtually no seasonal dynamic (Figure 1a). Water level drops in April and September 2019 resulted from pump defects. Water levels in the FSM‐E basin were precisely regulated for most of 2020 (WT drops due to sensor defects in May and November) and for all of 2021, resulting in six distinct WT classes spanning annual WTs from moderately rewetted (−0.22 m) to flooded conditions (+0.04 m) as anticipated in 2020 and 2021 (Figure 1b,c). Comparable to conditions at FSM‐F, mean annual WTs in the field‐scale site LM were −0.04 to 0.00 m. Moreover, intra‐annual water level dynamics were affected by rewetting measures (Figure 1d) rather than precipitation dynamics or surrounding site conditions (e.g., deep drainage landscape water levels). The drop in water level in June was due to the adjustment of the water management infrastructure to the sloping field relief. In the same year, WTs in RH showed high fluctuation dynamics but no seasonal trend, plausibly due to advanced soil degradation and surrounding site conditions (Figure 1e). Rewetting measures were not sufficient to counteract the dry spring and heavy rainfall in summer, which led to periods of unintended low water levels (min. −0.73 m) and prolonged high flooding (max. +0.29 m). The *Carex* treatment at slightly higher elevation along the drainage ditch (i.e., water supply) was particularly affected, with a resulting low mean annual WT of −0.29 m, whereas the mean annual WT in *Phalaris* could be maintained at −0.13 m.

#### 3.1.3 Biomass Development

We observed wide intraspecific ranges in biomass yields of the paludicultures, with wetter conditions leading to increases in yield across all species (Table 2). Yields span over similar values across the species, with 4.3 ± 0.3 to 10.3 ± 0.8 t DM ha^−1^ year^−1^ in *Carex*, 2.9 ± 0.2 to 15.0 ± 2.0 t DM ha^−1^ year^−1^ in *Phalaris*, 1.6 ± 0.8 to 11.9 ± 0.8 t DM ha^−1^ year^−1^ in *Phragmites*, and 1.8 ± 0.3 to 9.7 ± 1.5 t DM ha^−1^ year^−1^ in *Typha* sp. Vegetation surveys in the field‐scale sites in 2019 and 2021 (Hartbrich, 2024 unpubl. BA thesis, HSWT 2024) (Table 2) showed expected species‐specific stand densities of 95%–100% in *Carex* and 92%–99% in *Phalaris*. While target species cover of *Phragmites* reached 90%–99% in most stands, we observed a significant yield reduction in one moderately rewetted treatment in site FSM‐E in 2021. Similarly, *Typha* spp. exhibited a moderate, species‐typical cover of around 85% in rewetted plots, whereas its abundance greatly decreased in moderately rewetted stands—again with one treatment showing substantially lower biomass yields in site FSM‐E in 2021. These anomalies suggest site‐specific stressors related to suboptimal conditions for *Phragmites* and *Typha* plant development. Further, we described a decreasing trend in yield with stand age in *Carex*; however, all other species showed no clear stand age‐related trends. The additional summer harvest of *Phalaris* in FSM‐E and LM in 2021 led to increased yields compared with the one‐cut *Phalaris* treatments. The range in biomass Corg contents was 44.9% to 48.3% DM.

### 3.2 Greenhouse Gas Emissions Across Paludiculture Species

#### 3.2.1 Carbon Dioxide

Flux dynamics of CO_2_ followed the expected seasonal pattern in all species and across the treatments. Reco and GPP fluxes increased during the vegetation phases and with elevating temperatures (max. fluxes Reco 35.3 μmol m^−2^ s^−1^; GPP −82.9 μmol m^−2^ s^−1^, both in *Phragmites* rewetted), while harvest events in *Phalaris* during summer reduced GPP to near zero (Figure S2). CO_2_ flux dynamics further reflect species‐specific phenological phases. *Carex* and *Phalaris* began production of photosynthetically active shoots in early spring, whereas *Phragmites* initiated growth between April and May, and *Typha* spp. resumed growth in May. Higher water levels generally resulted in slightly reduced Reco fluxes and greater GPP fluxes, ensuing lower overall NEE balances. However, prolonged inundation of *Phalaris* at site RH in 2021 led to a marked reduction in growth, as reflected by low Reco and GPP fluxes and a low positive annual CO_2_ balance (Figure S2a). Moreover, differences in WT growth optima were apparent at the site FSM‐E in *Phragmites* and *Typha* spp. at lower WT, resulting in lower Reco and higher GPP (Figure S2b). In contrast, *Carex* and *Phalaris* show only marginal differences in flux strength under drier conditions. Resulting campaign‐specific models revealed Tair as the main driver for Reco independent of plant species (full model parameter tables are provided in repository Table R1). Overall, campaign‐based model evaluation metrics indicated good performance following Moriasi et al. (2007), with NSE > 0.5 in 84% of Reco and 94% of GPP models, suggesting satisfactory simulations. A slight model overestimation was observed, with mean PBIAS of −0.1 for Reco and −0.8 for GPP models (Figure S4). The mean RMSE giving the standard deviation of the model prediction error was 0.963 μmol CO_2_ m^−2^ s^−1^ for Reco and 1.821 μmol CO_2_ m^−2^ s^−1^ for GPP.

Annual balances of all CO_2_ flux components (Reco, GPP, and NEE) showed a clear negative correlation with WT despite substantial scatter in the dataset. The annual balances of Reco ranged from 41.3 ± 6.5 ( *T. latifolia* rewetted) to 114.2 ± 27.1 (*Phalaris* mod. rewetted) t CO_2_ ha^−1^ year^−1^; GPP balances from −131.6 ± 9.4 (*Phragmites* rewetted) to −43.0 ± 3.2 (*Phalaris* mod. rewetted); and NEE balances from −59.0 ± 0.9 t CO_2_ ha^−1^ year^−1^ (*Phragmites* rewetted) to 27.8 ± 10.6 ( *T. latifolia* mod. rewetted) t CO_2_ ha^−1^ year^−1^ (Table R2). We detected a persistent CO_2_‐source‐to‐sink transition in 77 of the 81 tested treatments in *Phalaris* as early as April and in *Typha* by the latest end of June Annual balances reflect the interaction between plant species and their species‐specific site preferences, i.e., WT optimum ranges, where *Carex* and *Phalaris* retain relatively large uptake capacities under all tested WT levels and *Typha* and *Phragmites*' sink strength shows only at higher WT. Overall, 77 of the 81 studied paludicultures had negative NEE balances, i.e., were a net sink of CO_2_. Positive NEE balances in single replicates resulted from larger Reco fluxes relative to the other replicates of the same treatment, i.e., large intra‐treatment variability. The metrics for model performance of the annual balances are provided in Table S2. In adherence to the current national emission reporting protocol, we give CO_2_–C_onsite_ and CO_2_–C_organic_ values in t C ha^−1^ year^−1^ in Table 3 (IPCC 2014; Tiemeyer et al. 2020). The relative contribution of fertilizer‐derived C to the CO_2_–C_onsite_ values in fertilized *Phalaris* treatments was 4% to 8%.

**TABLE 3:** Average emission factors (EF) (95th percentiles in brackets) for emission reporting of selected land‐use categories on organic soils in Germany as given by Tiemeyer et al. (2020) and proposed preliminary EF derived for fen paludicultures disaggregated by GHG and water table (WT) for the greenhouse gases (GHG) carbon dioxide (CO_2_), methane (CH_4_), nitrous oxide (N_2_O), and for total GHG balances.

Land‐use category	CO_2_–C_onsite_	CH_4land_	Direct N_2_O–N	CO_2_–C_organic_ ^a^	CH_4 organic_ ^b^	N_2_O–N_organic_	GHG balance^c^	
							IPCC AR4	IPCC AR5
	t C ha^−1^ year^−1^	kg CH_4_ ha^−1^ year^−1^	kg N_2_O–N ha^−1^ year^−1^	t C ha^−1^ year^−1^	kg CH_4_ ha^−1^ year^−1^	kg N_2_O–N ha^−1^ year^−1^	t CO_2_e ha^−1^ year^−1^	
Cropland	9.2 (3.8–11.2)	5.5 (0.5–17.9)	11.1 (1.8–40.5)	9.5	20.6	11.1	40.4	39.9^d^
Grassland	8.3 (1.4–11.0)	11.2 (0.6–86.4)	4.6 (0.3–22.2)	8.0	21.7	4.2	31.7	31.6^d^
Rewetted organic soils (defined as WT ≥ −0.1 m)	−0.4 (−2.4–1.3)	279 (140–700)	0.1 (−0.5–1.0)	−0.4	279.0	0.1	5.5	6.4^d^
Paludiculture rewetted, WT ≥ −0.1 m, *n* = 43	−5.5 (−10–2.8)	270.5 (43.9–663.7)	0.2 (−0.3–1.1)	−5.3	270.5	0.2	−12.8	−12.0
Paludiculture moderately rewetted, WT > −0.3 to < −0.1 m, *n* = 39	−0.74 (−5.5–5)	29.9 (−1.7–116.2)	1.5 (0.2–5.7)	−0.4	36.4	1.5	−0.1	−0.1

*Note:* Organic soils are considered rewetted where mean annual WT are ≥ −0.1 m below the surface. We further suggest an EF for moderately rewetted paludicultures where WT are between −0.3 and < −0.1 m below the surface.

^a^ Incl. IPCC default EF DOC: drained: CO_2_–C DOC = 0.31 t C ha^−1^ year^−1^, rewetted: CO_2_–C DOC = 0.24 t C ha^−1^ year^−1^; IPCC (2014).

^b^ Incl. default EFs CH_4_ ditch according to IPCC (2014) and Tiemeyer et al. (2020).

^c^ Incl. DOC and CH_4_ ditch.

^d^ Calculated from Tiemeyer et al. (2020) using IPCC AR5 GWP100 (28 for CH_4_ and 265 for N_2_O); Myhre et al. (2013).

#### 3.2.2 Methane

Methane flux magnitudes reflect clear differences between WT and paludiculture plant species. Under rewetted conditions, CH_4_ fluxes became more pronounced with increasing WT and followed the expected seasonal pattern, with highest fluxes from July to September when vegetation biomass and temperatures were highest (Figure S3). The dynamics further depended on plant species, where, on one hand, *Phalaris* and *Typha* spp. showed maximum summer fluxes of up to 55,000 μg m^−2^ h^−1^, whereas *Carex* and *Phragmites* remained well below 20,000 μg m^−2^ h^−1^ in summer despite high WT conditions.

**FIGURE 2:**
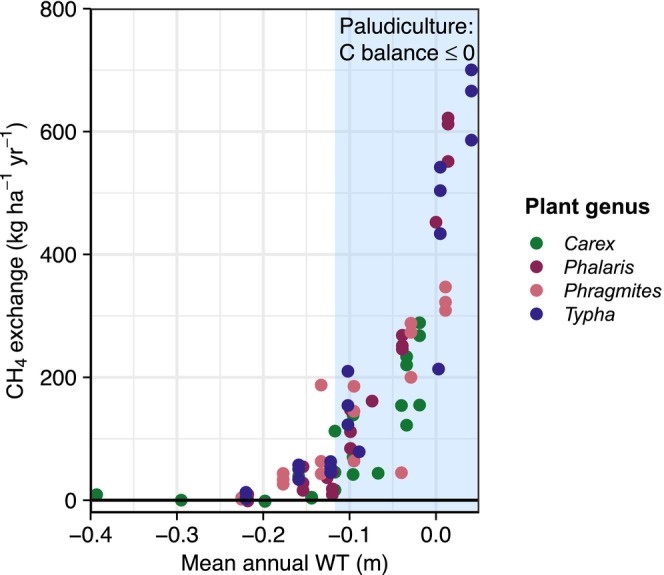
Responses of methane emissions (CH_4_ exchange in kg CH_4_ ha^−1^ year^−1^) to the mean annual water table (WT in m) for the paludicultures differentiated by plant genus (*n* = 105 replicate‐specific annual balances). The blue band shows the WT range where the net carbon (C) balance is ≤ 0 indicating peat‐preserving potential in line with the definition of paludicultures. Note that CH_4_ ditch emissions are included in the preliminary emission factors used for emission reporting where mean annual WT are < −0.1 m below the surface (CH_4organic_, Table 3).

The significant WT effect was further apparent in the annual CH_4_ exchange, with total balances remaining around zero (min. −2.0 kg CH_4_ ha^−1^ year^−1^) until mean annual WTs exceeded −0.2 m. Beyond this threshold, CH_4_ exchange became positive and exhibited an exponential relationship with WT (Figure 2). Plant genus significantly influenced CH_4_ exchange, with the highest fluxes in *Phalaris* and in *Typha* spp. reaching a maximum of 700.3 ± 46.3 kg CH_4_ ha^−1^ year^−1^ in flooded *T. latifolia* (Table R2). We further found a significant positive effect of annual biomass yield on the annual CH_4_ exchange. Overall, in our CH_4_ exchange model, a high proportion of the variance was explained by the covariates (marginal *R* ^2^ of 0.88). Site as a random effect contributed marginally to the explained variance (conditional *R* ^2^ of 0.90; interclass correlation coefficient ICC = 0.12). CH_4_ exchange is reported as CH_4land_ and CH_4organic_ in kg CH_4_ ha^−1^ year^−1^ in Table 3.

#### 3.2.3 Nitrous Oxide

**FIGURE 3:**
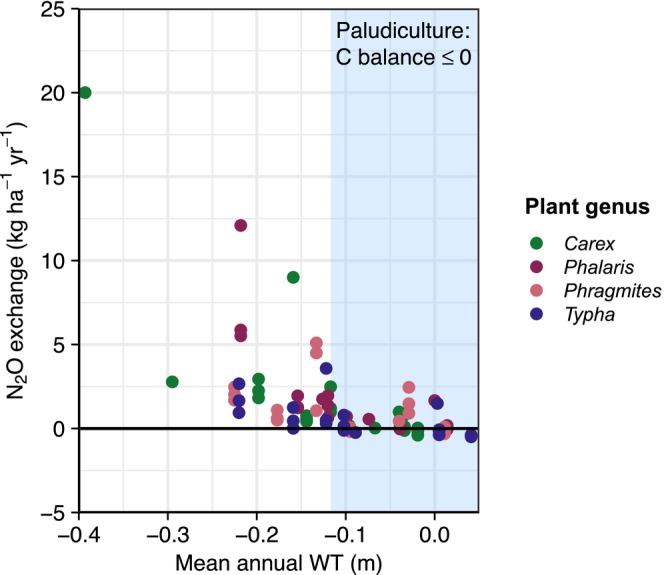
Responses of nitrous oxide emissions (N_2_O exchange in kg N_2_O ha^−1^ year^−1^) to the mean annual water table (WT in m) for the paludicultures differentiated by plant genus (*n* = 105 replicate‐specific annual balances). The blue band shows the WT range where the net carbon (C) balance is ≤ 0 indicating peat‐preserving potential in line with the definition of paludicultures. Note that direct N_2_O–N and N_2_O–N organic are given in kg N_2_O–N ha^−1^ year^−1^ for emission reporting (Table 3).

Nitrous oxide showed higher dynamics in background fluxes under lower WT conditions and lower dynamics with values around zero in the wetter paludiculture treatments throughout the year (Figure S3). Fertilization of *Phalaris* in 2021 led to single peaks in N_2_O fluxes that were more pronounced under lower WT conditions (max. 4328.8 μg m^−2^ h^−1^) (see Figure S3b). Annual N_2_O exchange ranged from −0.5 ± 1.1 to 12.1 ± 1.6 kg N_2_O ha^−1^ year^−1^ (Table R2). We detected a significant negative WT association, with emissions decreasing approximately quadratically as mean annual WT increased (Figure 3). Plant genus significantly affected N_2_O emissions. *Phalaris* showed significantly higher N_2_O balances compared with *Carex* and *Phragmites*, while *Typha* showed the lowest N_2_O emissions. We found a significant effect of stand age, i.e., time since establishment. The most recently established treatments (stand age 1 year) exhibited significantly larger N_2_O emissions compared with stand age ≥ 2 years. Most of the variation in the N_2_O flux dataset could not be explained by our covariates (marginal *R* ^2^ of 0.40, dispersion factor = 1.02). Site as a random effect did not contribute to the explained variance (conditional *R* ^2^ of 0.40, ICC = 8.42e^−08^). N_2_O exchange is reported as direct N_2_O–N and N_2_O–N_organic_ in kg N_2_O–N ha^−1^ year^−1^ in Table 3.

### 3.3 Net Ecosystem Carbon Balances, GHG Balances, Emission Factors, and Optimal WT for Maximum Mitigation

We observed the greatest net C uptake of −13.6 ± 0.6 t C ha^−1^ year^−1^ in *Phragmites* at a mean WT of −0.04 m and the highest net C loss of 8.3 ± 2.9 t C ha^−1^ year^−1^ in *T. latifolia* at a mean WT of −0.16 m (Figure S5, Table R2). NECBs are very similar to the C_onsite_ values, as the maximum relative CH_4_‐C contribution to the C balance merely amounted to 3% in *Typha* under flooded WT conditions. Net C losses decreased nonlinearly with increasing WT. We deduced a shift from net C source‐to‐sink from our GAMM at a threshold WT of −0.12 m (Figure 3 and Figure S5). Besides WT, plant genus and management intensity were found to significantly affect NECB. Disaggregated by plant genus, the estimated WT threshold for net C balances ≤ 0 are −0.14 m for *Carex*, −0.13 m for *Phragmites*, −0.10 m for *Typha*, −0.12 m for *Phalaris* (1 cut), and −0.10 m for *Phalaris* (2 cut).

**FIGURE 4:**
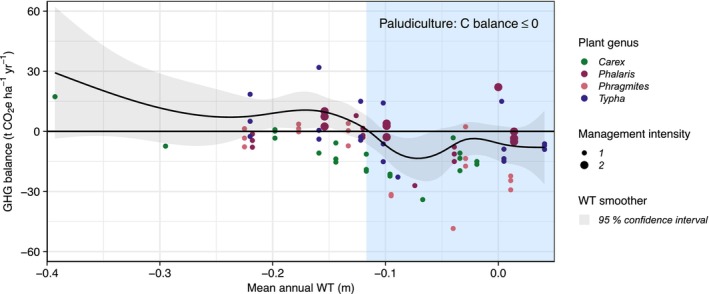
Responses of greenhouse gas (GHG) balances (t CO_2_e ha^−1^ year^−1^) to the mean annual water table (WT in m) for the paludicultures differentiated by plant genus and management intensity: 1 = one winter harvest; 2 = one summer harvest + one winter harvest + fertilization (in FSM‐E treatments only). *n* = 83 annual treatment (field‐scale sites) and replicate (FSM‐E) balances. The blue band shows the WT range where the net carbon (C) balance is ≤ 0 indicating peat‐preserving potential in line with the definition of paludicultures. The black line gives the estimated nonlinear relationship between GHG balances and mean annual WT (smoother in the GAMM), with the grey band showing the 95% confidence interval.

GHG balances ranged from −48.3 ± 2.1 to 32.0 ± 11.0 t CO_2_e ha^−1^ year^−1^ (AR5). As observed in the NECB, *Phragmites* at a mean WT of −0.04 m acted as the greatest GHG sink, while *T. latifolia* at a mean WT of −0.16 m was the greatest GHG source (Figure 4, Table R2). The average contribution of biomass C export, CH_4_, and N_2_O to the GHG balances was 34% (min. 8%, max. 71%), 9% (min. 0%, max. 26%), and 2% (min. 0%, max. 13%), respectively. We observed a significant highly nonlinear effect (effective degrees of freedom = 6.281) of mean annual WT on total GHG emissions. The nonlinear relationship shows a WT optimum of −0.07 m for maximum GHG mitigation potential in this fen paludiculture dataset (Figure 4). All treatments with a mean annual WT of −0.07 ± 0.03 m led to negative GHG balances. *Carex* and *Phragmites* had significantly greater GHG mitigation potential compared to *Phalaris* and *Typha* (Table 3). Moreover, the increased management intensity in *Phalaris* resulted in significantly higher GHG balances compared with all one‐cut treatments. Site as a random effect in our GAMM had a high ICC (0.63), indicating a significant contribution to the explained variance (adjusted *R* ^2^ of 0.52).

From CO_2_–C_onsite_, CH_4land_ and direct N_2_O emissions, and accounting for CO_2_ emissions from DOC and CH_4_ emissions from ditches (IPCC default), we calculated a preliminary EF of −12.0 (−32.9 to 16.4) t CO_2_e ha^−1^ year^−1^ for ‘rewetted fen paludiculture’ and of −0.1 (−17.6 to 20.4) t CO_2_e ha^−1^ year^−1^ for ‘moderately rewetted fen paludiculture’. Detailed preliminary EFs for CO_2_–C_organic_, CH_4organic_, N_2_O–N_organic_ and total GHG emissions are given in Table 3.

## 4 Discussion

### 4.1 Greenhouse Gas Exchange in Fen Paludicultures

The GHG emissions observed in the studied fen paludicultures under moderately to completely rewetted conditions demonstrate considerable emission reduction potentials. To date, annual GHG emission budgets for fen paludicultures remain scarce, and the few available studies on simulated or newly established sites report emissions ranging from moderate CO_2_ sources to small sinks. Compared to simulated paludicultures of rewetted *Carex*, *Phragmites*, and *Typha* in a restored fen (Günther et al. 2015), we observed a substantially higher CO_2_ uptake. In contrast, newly planted paludicultures of flooded *T. angustifolia* and *T. latifolia* showed CO_2_ uptakes and estimated yields comparable to our observations (Van Den Berg et al. 2024). Reported yields in newly established *Phalaris* paludicultures under intensive harvest and fertilization regimes (Kandel et al. 2020; Rodriguez et al. 2024) were like those in our higher‐intensity, two‐cut *Phalaris* systems, conceivably reflecting optimal nutrient supply from fertilization. However, fertilization led to significantly higher N_2_O emissions, and the slightly to significantly reduced NEE could partly be the result of earlier harvesting, affecting both GPP and Reco.

Compared to the German climate reporting for the land‐use category “rewetted organic soils” with emission estimates of −0.4 t CO_2_–C ha^−1^ year^−1^ (reported as “CO_2_–C onsite”, including direct CO_2_ emissions and biomass C export) (Tiemeyer et al. 2020), the net CO_2_ emissions in our rewetted paludiculture were substantially more negative at −5.5 t CO_2_–C ha^−1^ year^−1^, indicating higher CO_2_ uptake under the same WT definition (Table 3). Notably, the national EF aligns more closely with the moderately rewetted paludicultures in our dataset with −0.7 t CO_2_–C ha^−1^ year^−1^, albeit with higher variability. These findings support general trends of lower CO_2_ emissions with increasing WTs (Tiemeyer et al. 2020; Evans et al. 2021; Koch et al. 2023) and suggest that paludicultures can achieve near‐zero CO_2_ onsite emissions, even under moderately rewetted conditions and accounting for biomass export. Across all paludiculture plants, the ratio of net photosynthesis (approximately 50% of GPP) to C export averaged 4.8, indicating that belowground C fixation in roots and rhizomes likely far exceeds aboveground biomass production. This aligns well with studies by Nielsen et al. (2021), who found that 70% of the total biomass production of *Phalaris* is stored underground. Nevertheless, our observations cover only the initial 1 to 5 years of establishment, where enhanced root and rhizome growth is expected. Hence, statements regarding the long‐term CO_2_ sequestration potential of paludiculture are still uncertain. However, stand age had no significant effect in our GHG balance model, and net negative NEE has been reported for long‐term restored eutrophic fens (Minke et al. 2016).

The current German EFs for CH_4_ and N_2_O exchange for the land‐use category “rewetted organic soils” (279.0 kg CH_4_ ha^−1^ year^−1^ and 0.1 kg N_2_O–N ha^−1^ year^−1^; Tiemeyer et al. 2020) corroborate with the aggregated mean emissions from our rewetted paludicultures (270.5 kg CH_4_ ha^−1^ year^−1^ and 0.2 kg N_2_O–N ha^−1^ year^−1^) (Table 3). Methane emissions increase when anaerobic conditions are reestablished in rewetted organic soils, facilitating methanogenesis from readily available C sources, particularly during warm seasons (cf. syntheses by Turetsky et al. (2014) or Wilson et al. (2016)). As in the national dataset, observed CH_4_ emissions start to increase exponentially above a WT of −0.2 m (Tiemeyer et al. 2016, 2020). Wetland plants such as sedges, reeds, and cattails further amplify emissions by facilitating CH_4_ transport from the soil via aerenchyma, bypassing the oxic zone. The observed rise in CH_4_ emissions with increased biomass yields is likely associated with greater below‐ground biomass production and an increase in photosynthate transport to the plant rhizosphere, favoring methanogenesis from root exudates. Persistent flooding increased CH_4_ emissions with maxima of 700 kg ha^−1^ year^−1^ in slightly flooded *T. latifolia* stands, comparable to values reported for young *T. latifolia* in a fully flooded coastal fen (Van Den Berg et al. 2024). While we observed significantly lower CH_4_ emissions in *Carex* and *Phragmites* paludicultures, this may not hold true for rewetted *Carex* spp.‐dominated stands in general. The national dataset includes values of 1380–1600 kg CH_4_ ha^−1^ year^−1^ in rewetted *Carex*‐moss fens (Tiemeyer et al. 2020), and older *Carex* stands under persistent flooding also exhibited high emissions (Günther et al. 2015). As expected, lower WTs resulted in significantly decreased CH_4_ emissions in all plant species, evident in mean emissions of 29.9 kg ha^−1^ year^−1^ in our moderately rewetted paludicultures (Table 3). Peak CH_4_ emissions, often cited as negating mitigation benefits from rewetting, were not observed in our paludiculture sites, where moderate emissions in the first 1–5 years did not offset mitigation benefits. Initial peaks are linked to rewetting flood‐intolerant species (Tiemeyer et al. 2016; Freeman et al. 2022) or vegetation succession after rewetting (IPCC 2014; Minke et al. 2016; Wilson et al. 2022). Unlike natural wetland restoration, precise WT and vegetation management in paludicultures bypass transition phases, avoiding large organic matter inputs from initial plant die‐off.

N_2_O emissions are lower in fully rewetted organic soils but higher in drained peatlands, where large amounts of the naturally stored N in peat are released via decomposition (e.g., Wang et al. 2024 GCB; Mander et al. 2025). Highest N_2_O fluxes are found in nutrient‐rich drained peatlands and are associated with water levels fluctuating between −0.2 and −0.5 m or when water‐filled pore space exceeded 70% (Ruser et al. 2006; Goldberg and Gebauer 2009; Tiemeyer et al. 2016; Mander et al. 2025). Under rewetted conditions, N nitrification is greatly restricted by low oxygen availability, providing limited substrate for denitrification processes (Butterbach‐Bahl et al. 2013). The IPCC deems N_2_O emissions from rewetted organic soils negligible for reporting, setting them to zero, but recommends developing national EFs where local N inputs, soil enrichment, or water level fluctuations may cause sporadic oxygenation (IPCC 2014). Due to the intensive land‐use history of peatlands in Germany, a national EF for direct N_2_O emissions in rewetted organic soils has been suggested by Tiemeyer et al. (2020). Our observations are consistent with the expected N_2_O exchange pattern, showing net emissions around zero under rewetted conditions and an increase under moderate rewetting associated with ideal soil conditions for denitrification (Table 3). Interestingly, potentially supporting the rationale for national EFs, our dataset showed significantly higher N_2_O emissions in stands aged 1 year compared to older stands, which could reflect the initial influence of nutrient‐rich conditions from prior land use. While we observed significantly higher N_2_O exchange in *Phalaris* stands, cutting frequency and organic fertilization did not significantly increase emissions (max. 1.3 kg N_2_O–N ha^−1^ year^−1^). However, mineral fertilization in rewetted *Phalaris* paludicultures with two harvests led to emissions up to 6.0 kg N_2_O–N ha^−1^ year^−1^ (Kandel et al. 2020).

Total GHG emissions in our paludicultures considerably undercut published GHG balances from field studies in temperate rewetted fens. For example, Günther et al. (2015) reported 0.4 to 31.1 t CO_2_e ha^−1^ year^−1^ for *Carex* stands (WT −0.01 to 0.10 m), 3.7 to 17.3 t CO_2_e ha^−1^ year^−1^ for *Phragmites* (WT −0.20 to 0 m), and 13.0 to 13.1 t CO_2_e ha^−1^ year^−1^ for *T. latifolia* (WT −0.06 to 0.03 m) from direct flux (excluding N_2_O) and harvest measurements in simulated paludicultures. To date, the only field studies from cropping paludiculture sites covering growth and harvest cycles report annual GHG emissions of −1.4 and 10.5 t CO_2_e ha^−1^ year^−1^ for *T. angustifolia* and *T. latifolia* , respectively, inclusive of C export deduced from nearby biomass harvests (Van Den Berg et al. 2024), and 10.4 to 36.4 t CO_2_e ha^−1^ year^−1^ in rewetted, more intensively managed *Phalaris* (Kandel et al. 2020; Rodriguez et al. 2024). We relate the distinctly higher emissions reported by previous studies to probable quality issues related to GHG measurement methods, WT, or inaccurate biomass sampling and harvest simulations. For completeness, full GHG balances from *Sphagnum* paludicultures in bogs span −1.8 to 10 t CO_2_e ha^−1^ year^−1^ (Oestmann et al. 2022; Daun et al. 2023). Existing predictions for GHG emissions of various paludicultures indicate an equally wide but lower range of outcomes—yet consistently point to net positive emissions, highlighting the influence of site conditions and CH_4_ emissions. Estimated GHG emissions for paludicultures range from 0 to 18 t CO_2_e ha^−1^ year^−1^ (Geurts and Fritz 2018; Kasimir et al. 2018; Bianchi et al. 2021; Närmann and Tanneberger 2021). Discrepancy mainly arises from the scarce data basis (i.e., rewetted grassland; limited range of pre‐use, etc.), underlying assumptions (near‐zero NEE under wet conditions), or the use of proxies. While all tested paludicultures in this study exhibited significantly lower overall emissions, the strongest net negative GHG balances in the peat‐forming species *Carex* and *Phragmites* are likely driven by their physiological traits. Despite similar above‐ground biomass, below‐ground C sequestration appears more efficient in *Carex* and *Phragmites*, possibly due to greater below‐ground biomass production or reduced decomposition of more recalcitrant rhizomes and roots, i.e., the main peat‐forming parts. Moreover, differences in root morphology (e.g., porosity or volume) are linked to CH_4_ transport capacity (Määttä and Malhotra 2024), which may have contributed to the less favorable GHG balances observed in *Phalaris* and *Typha*. Besides plant genus, we identify WT management and timing of harvest events as the main reasons for the large differences in GHG balances between the previously reported values and our observations, explaining a substantial proportion of the observed variability. Furthermore, site as a random effect contributed notably to the large variability in the GHG balances (cf. Tiemeyer et al. 2016), underscoring the influence of land‐use history and nutrient status on GHG emissions. Soil data analysis showed greater topsoil degradation in LM and RH and higher nutrient stocks in LM (Table S1, Figure S1). The high variability observed suggests that nutrient‐rich sites may offer greater relative GHG mitigation potential through paludiculture, as drained agricultural systems on such sites likely exhibit higher emissions than those on nutrient‐poor soils. Implementing paludiculture on nutrient‐rich land could also mitigate nutrient runoff through filtration processes, as demonstrated in field studies with *Phragmites* and *Typha* (Meuleman et al. 2002; Geurts et al. 2020; Van Den Berg et al. 2024). Additionally, these sites may support prolonged biomass production due to pre‐existing soil nutrient loads, with *Phragmites* having a potential competitive advantage due to their extensive root system and high nutrient‐use efficiency.

### 4.2 Management

Water management was identified as the primary factor governing the magnitude of GHG emissions. Our GHG balance model predicts an optimal WT for the maximum GHG mitigation potential of fen paludicultures at a mean annual WT of −0.07 m, with GHG balances increasing again toward WT of 0 due to increased CH_4_ exchange (Figure 4). This coincides with previous estimates of −0.1 and 0 m for best climate impact (e.g., Jungkunst et al. 2008; Jurasinski et al. 2016; Evans et al. 2021). Under these optimal WT conditions, all tested paludiculture species showed successful plant establishment and high biomass yields, except for *Typha* spp., which exhibited greater biomass development at WT above the surface only (Eickenscheidt et al. 2023). The high target species cover in fully rewetted sites reflects a competitive advantage under saturated soil conditions. In contrast, the lower abundance under moderate rewetting of *Typha* spp. suggests suboptimal hydrological conditions. Overly low biomass yields of the *Phragmites* and *T. latifolia* treatments at WT of −0.18 and −0.16 m, respectively, likely resulted from prolonged plant stress caused by suboptimal conditions relative to the species' growth optima, as their WT was between −0.2 and −0.3 m the previous 4 years.

WT management should correspond to management events, target plant species, and site conditions. Particularly after harvest, the WT should remain below the cut stalk height to prevent plant inundation, which can cause die‐off and increased GHG emissions, as observed in *Phalaris* in RH and in studies by Günther et al. (2015) and Kandel et al. (2020). Similarly, we observed that prolonged or excessive flooding entails large CH_4_ emissions, even in productive adapted fen communities. Particularly at restored sites where *Phragmites* was dominant and net CO_2_ balances were mostly negative, high CH_4_ emissions were reported to turn these sites into net C sources (Günther et al. 2015; Minke et al. 2016; Van Den Berg et al. 2016; Tiemeyer et al. 2020). In paludicultures, this effect is exacerbated by additional C export from harvests, which depends on the timing and frequency of harvest events. If increased yields are deemed necessary for greater biomass production or a decline in yields requires fertilization to maintain adequate biomass quality and quantity, the potential climate impact of such intensification must be carefully considered. Increased management intensity has mostly been tested in *Phalaris*, with higher overall emissions reported for fertilized two‐ and five‐cut paludicultures, predominantly resulting from reduced GPP due to summer harvest and, in part, higher CH_4_ and N_2_O emissions (Kandel et al. 2020; Rodriguez et al. 2024). Likewise, our observation in intensively managed *Phalaris* stands indicates increased overall emissions in comparison with extensive management, but the general GHG sink function remained intact under rewetted conditions, albeit at a reduced level. Contrasting results from a few field studies do not yet allow for reliable conclusions regarding the effect of fertilizing on N_2_O emissions and yield stabilization. Rodriguez et al. (2024) report that more than two harvest events could not increase yields—even with fertilization. However, in Germany, the fertilizer ordinance currently prohibits fertilizer application in water‐saturated soils such as paludiculture to prevent potential nutrient contamination of groundwater.

### 4.3 Implications for Policy

The overriding idea that paludiculture should be peat‐preserving necessitates a neutral or negative NECB. To date, paludiculture has been classified by WT levels of ≥ −0.10 m as a proxy, but it is lacking empirical evidence for its peat‐preservation effectiveness. The majority of observed NECBs in our paludiculture were negative, with a predicted overall WT threshold of −0.12 m for NECB ≤ 0, agreeing with the previously proclaimed WT minimum of −0.10 m. As for total GHG balances, threshold differences between plant genera and management intensities exist, with *Carex* stands at the lower WT end (≥ −0.14 m) and *Typha* and intensive *Phalaris* at the high end of ≥ −0.10 m. In general, we ascribe the consistent net C uptakes primarily to the high observed GPP values and moderate CH_4_ emissions even under high WTs. While empirically proven C sequestration may not be prioritized by most landowners, they may be more inclined to adopt robust management practices that prevent land degradation and promote resilient production systems (Kasimir et al. 2018; Freeman et al. 2022).

Our calculated preliminary EF aggregated for ‘rewetted fen paludiculture’ at mean annual WTs ≥ −0.1 m is in accordance with current national and international definitions of “rewetted organic soils” (IPCC 2014; Tiemeyer et al. 2020), amounting to −12.8 t CO_2_e ha^−1^ year^−1^ (AR4; AR5: −12.0; Table 3). The unprecedented large net GHG uptake for fen paludicultures far exceeds the German EF for the land‐use category “rewetted organic soils” of 5.5 t CO_2_e ha^−1^ year^−1^ (AR4; Tiemeyer et al. 2020). This difference could be explained by the underlying national dataset, which predominantly comprises restored bogs and presumably oligotrophic to mesotrophic fens, as they are more accessible for restoration due to their low agricultural value and exhibit lower CO_2_ uptake compared to rewetted eutrophic fens (Wilson et al. 2016; Nugent et al. 2018). In comparison, our results indicate greater climate benefits from fen paludiculture than from restored peatlands, at least in the initial years following establishment. As expected, we observed an increase in total GHG emission at lower WTs, with aggregated values for ‘moderately rewetted fen paludiculture’ of −0.1 t CO_2_e ha^−1^ year^−1^ (AR4; AR5: −0.1; Table 3). The proposed EF for paludiculture under moderately rewetted conditions captures a realistic range of field conditions, including datasets with suboptimal growing conditions such as the 2021 GHG balances of *Phalaris* (RH), *Phragmites*, and *Typha* (FSM‐E). Further WT drawdown likely reinstates strong GHG sources in designated paludiculture systems, as reported for *Carex*, where emissions increased from −11.1 t CO_2_e ha^−1^ year^−1^ (AR5) at a WT of −0.16 m to 16.8 t CO_2_e ha^−1^ year^−1^ at a WT of −0.39 m (Bockermann et al. 2024). As more empirical data become available, implementing Tier 3 emission reporting and disaggregating EFs by species and their respective transition phases would be advisable to reduce uncertainties in the proposed EFs.

Considering the high GHG emissions of 40.4 t CO_2_e ha^−1^ year^−1^ (AR4) from current agricultural use of organic soils as cropland in Germany (Tiemeyer et al. 2020), paludiculture under rewetted conditions yields a mitigation potential of up to 53.2 t CO_2_e ha^−1^ year^−1^ (AR4; AR5: −51.9 t CO_2_e ha^−1^ year^−1^; Table 3). Thus, our results indicate larger mitigation potentials than currently propagated for temperate paludicultures, with best estimates varying between reductions of 7 to 30 t CO_2_e ha^−1^ year^−1^ (Geurts and Fritz 2018; Bianchi et al. 2021; Tanneberger et al. 2022), currently making fen paludiculture the most effective nature‐based climate solution. Reduced N_2_O emissions from rewetted paludiculture on organic soils can further contribute to achieving C neutrality in the agriculture sector, presently accounting for 27% of the total direct sectoral N_2_O emissions (UBA 2024). This added value from paludicultures persists even under moderately rewetted soil conditions, as total emissions remain near zero and maintain a strong mitigation potential in comparison with all other land‐use categories on organic soils. Additionally, the mitigation potential further increases as biomass utilization substitutes fossil fuels, particularly in durable products like insulation and construction panels, which offer long‐term C storage and cascading use. While the ideal situation from a nature conservation perspective would be peatland rewetting with natural plant colonization, ambitious large‐scale implementation of paludiculture could provide the indispensable contribution urgently needed to secure and expand the net sink function of the LULUCF sector, fulfill EU nature restoration goals, and support regional economies.

To fully realize this potential, a comprehensive political, economic, and legal approach is imperative. One that actively involves agriculture and establishes paludiculture as a key component of the national climate protection strategy. Thus far, national peatland strategies developed by eight European countries over the past decade address national concerns as well as international agreements (Nordbeck and Hogl 2024). More recent (post‐Paris) strategies reflect the value of peatlands as nature‐based solutions to climate change, with Germany solely and explicitly including paludiculture in suggested measures. To meet national climate protection targets, transformation pathways for Germany to reach net zero CO_2_ emissions from peatlands require a stepwise approach to rewetting all organic soils until 2050 (Tanneberger et al. 2021; Wichmann and Nordt 2024). Estimated area potentials for transforming drained organic soils into paludiculture vary between ca. 18% (325,000 ha; Prognos et al. 2020) and 58% (1,045,500 ha; Schäfer et al. 2022) until 2050. The most efficient measure could entail converting all cropland on fen peat and fen peat‐derived organic soils (213,862 ha; Wittnebel et al. 2023) into rewetted paludiculture, resulting in annual emission savings of 11.1 Mt CO_2_e using our preliminary EFs, only reducing Germany's cropland by 1.7%. For comparison, the conversion of about 1.9 million ha of land to forest would yield an equally large emission reduction (cf. EEA 2025), highlighting the benefits of organic soil cropland conversion to paludiculture in terms of land‐use efficiency and considering affiliated actors. A further target for climate action warrants the conversion of intensively used grassland on drained organic soils with a current EF of 31.7 t CO_2_e ha^−1^ year^−1^ (AR4; Table 3). While grassland rewetting may result in bidirectional GHG emission outcomes under moderately rewetted conditions (Evans et al. 2021; Offermanns et al. 2023; Bockermann et al. 2024; Tiemeyer et al. 2024; Heller et al. 2025), conversion to rewetted paludiculture could offer an additional mitigation potential of up to 44.5 t CO_2_e ha^−1^ year^−1^ (AR4; AR5: −43.6 t CO_2_e ha^−1^ year^−1^). A profound strategy would be the inclusion of paludiculture as eligible under the EU's Common Agricultural Policy (CAP). However, the necessary policy frameworks are still missing, and unsustainable land use remains heavily subsidized, further counteracting mitigation goals. The use of drained peatlands for agriculture in Germany annually receives about € 400 million in subsidies, entailing an economic damage estimated at nearly € 9 billion annually (Schäfer et al. 2022). This mismatch of effective planning and funding measures prevents farmers and landowners from implementing paludiculture unless other financial incentives are explored.

Generating revenue from fen paludiculture biomass—e.g., through energetic or material utilization (Hartung et al. 2020, 2023; Krus et al. 2021; Kuptz et al. 2022)—is inherent to the definition of paludiculture. The economic analysis of currently achievable contribution margins, excluding the monetization of GHG mitigation costs, reiterates the insufficient profitability of paludiculture under current market conditions (Eickenscheidt et al. 2023), thus hindering implementation. Until market incentives or a state‐funded climate premium are established (such as, e.g., the Bavarian “Moorbauernprogramm” 2023), financial risks for landowners could be reduced by integrating paludiculture into frameworks like the Voluntary Carbon Market. For example, the EU Carbon Removals and Carbon Farming Certification (CRCF) Regulation, adopted in 2024, involves practices that enhance C sequestration in soils or reduce GHG emissions from soils (European Commission 2024). Focusing on C credit markets is expected to be far more profitable than biomass utilization, as C credits will likely outpace the financial returns from sustainably sourced products, even with increasing demand and willingness to pay for sustainability. However, the valuation of the benefits depends on the specific primary aims of paludiculture, i.e., biomass production, nature conservation, or climate protection. While it is crucial to prioritize fast implementation rather than focusing solely on short‐term monetary gains, economic opportunities for local communities and sustainable land use must be equally considered. Nevertheless, large‐scale land‐use transformation must go beyond short‐term profits, making resilience to future climate change a priority. Compared with the current use, paludiculture on rewetted peatlands creates robust agricultural systems, enhancing key ecological structures and processes (e.g., IPCC 2014; Beyer et al. 2021; Schwieger et al. 2022). Recent findings by Bockermann et al. (2024) demonstrated persistent mitigation potentials of *Carex* paludicultures under a predicted mid‐term warming scenario in southern Germany, while conventional grassland emissions were exacerbated. Resiliency against heat stress has additionally been reported for rewetted fens and *Sphagnum* farming sites (Koebsch et al. 2020; Beyer et al. 2021; Oestmann et al. 2022). Despite the need for long‐term studies and analyses across diverse species ranges and the potential critical roles of WT and nutrient management, the findings are promising regarding yield stability and underscore the pertinence of promoting the rapid implementation of well‐managed paludicultures.

## 5 Conclusions

According to the German government's Climate Protection Plan 2050, the land‐use sector must remain a net GHG sink until at least 2030. However, current projections suggest this target will not be met unless more ambitious measures are adopted, and drainage‐based peatland incentives are phased out. Our field study, based on 83 annual GHG balances along a rewetting gradient, provides the first comprehensive database on the climate relevance of potential paludiculture species under various management practices. We clearly demonstrate the substantial climate protection achievable through rewetting organic soils and using them as paludiculture. Our study underscores the critical role of adaptive WT management for both climate impact and biomass development. Fen paludicultures currently show the highest empirically proven mitigation potential among land‐use measures, representing one of the most efficient and cost‐effective nature‐based climate solutions. Although long‐term carbon sequestration in fen paludicultures requires ongoing monitoring, the observed initial savings are promising for immediate climate benefits and emission offsetting.

## Author Contributions

**Carla Bockermann:** data curation, formal analysis, investigation, visualization, writing – original draft, writing – review and editing. **Tim Eickenscheidt:** conceptualization, data curation, formal analysis, funding acquisition, investigation, methodology, project administration, software, supervision, visualization, writing – review and editing. **Matthias Drösler:** conceptualization, funding acquisition, project administration, resources, supervision, writing – review and editing.

## Conflicts of Interest

The authors declare no conflicts of interest.

## Supporting information


Data S1.

## Data Availability

The data supporting the findings of this study and R code for data exploration are openly available in Zenodo at https://doi.org/10.5281/zenodo.16026838.
