# Supplementary material for: Greenhouse Gas Mitigation Potential of Temperate Fen Paludicultures
Source: Glob Chang Biol. 2025 Jul 30;31(8):e70385. doi: 10.1111/gcb.70385 (PMC12311260; doi:10.1111/gcb.70385)
Supplement: Supplementary file 1 — Data S1. [file GCB-31-e70385-s001.docx]

Supplementary Information

**Greenhouse gas mitigation potential of temperate fen paludicultures**

Carla Bockermann*, Tim Eickenscheidt & Matthias Drösler

*corresponding author E-mail: carla.bockermann@hswt.de

The Supplementary Information contains an extended description of the methods including Supplementary Figure S1 and Table S1 in sections S1-3, as well as Figures S2-S5 and Tables S2 to complement the results section of the main manuscript.

Additional data and code that support the findings of this study are openly available in Zenodo at <https://doi.org/10.5281/zenodo.16026838>

# Extended description of methods

## S1 Soil properties

We classified the soil at site FSM as a Sapric Histosol according to the IUSS Working Group WRB (2022), corresponding to an “Erdniedermoor” in the German soil classification system (KA6, AG Boden, 2024). The peat layer ranged from 2.55–2.90 m in thickness and consisted mainly of “radicels” sedge peat (Hnr), with varying admixtures of alder (Hnle) and *Phragmites* peat (Hnp). The upper 0.34–0.40 m was earthified (nHv) amorphous peats without recognizable structure and with high von Post value of decomposition of H8–H9 (von Post, 1924). The soil at site LM was also classified as a Sapric Histosol (“Erdniedermoor”) with a 2.30–3.65 m peat layer, mainly composed of “radicels” sedge peat (Hnr), again with varying admixtures of alder (Hnle) and *Phragmites* peat (Hnp). The upper 0.37–0.54 m was earthified (nHv) to strongly earthified (nHm) peats without recognizable structure (Ha), exhibiting von Post decomposition values of H8–H9. The soil at site RH was classified as a Mollic Gleysol or “Moorfolgeboden” (peat derived organic soil) with a 0.26–0.65 m peat layer without recognizable structure (Ha). The upper 0.26–0.58 m was earthified (nHv) to strongly earthified (nHm) peat and exhibited advanced decomposition (von Post H9–H10).

From three soil profiles excavated at each study site (FSM, LM, RH) in 2021, we determine soil/peat type, the horizons, the von Post value of decomposition, and the peat depths down to the underlying mineral layer. Further, from each profile we determined the most important chemical and physical soil or peat parameters down to 1.0 m depth respectively down to the mineral horizon (**Figure S1**) by taking soil core samples (rings 100 cm^3^) every 5 cm to a depth of 50 cm, along with a disturbed mixed sample every 10 cm. For samples below 50 cm, we combined the depth levels of 50 – 70 cm and 70 – 100 cm. Chemical analyses were conducted by AGROLAB (AGROLAB GmbH, Germany). To further investigate peat quality, soil samples of one profile next to the GHG measurement treatments were analyzed via standard ^13^C solid-state cross polarization magic angle spinning (MAS) NMR spectra as described in Säurich et al. (2021) at the Institute of Soil Science at the TU Munich, Germany, using a Bruker DSX 200 (Bruker BioSpin GmbH, Karlsruhe, Germany). **Carboxyl C contributes to soil organic matter rea**ctivity and stabilization through interactions with minerals, Aryl C indicates lignin content and degradation stability, O-alkyl C represents readily degradable carbohydrates such as cellulose and hemicellulose, and Alkyl C reflects hydrophobic, aliphatic compounds that are more resistant to decomposition and contribute to long-term carbon sequestration. The Alkyl C:O-alkyl C ratio serves as an indicator of soil organic matter turnover potential, with increasing values suggesting greater microbial processing and decomposition. Results are given in **Table S1**.


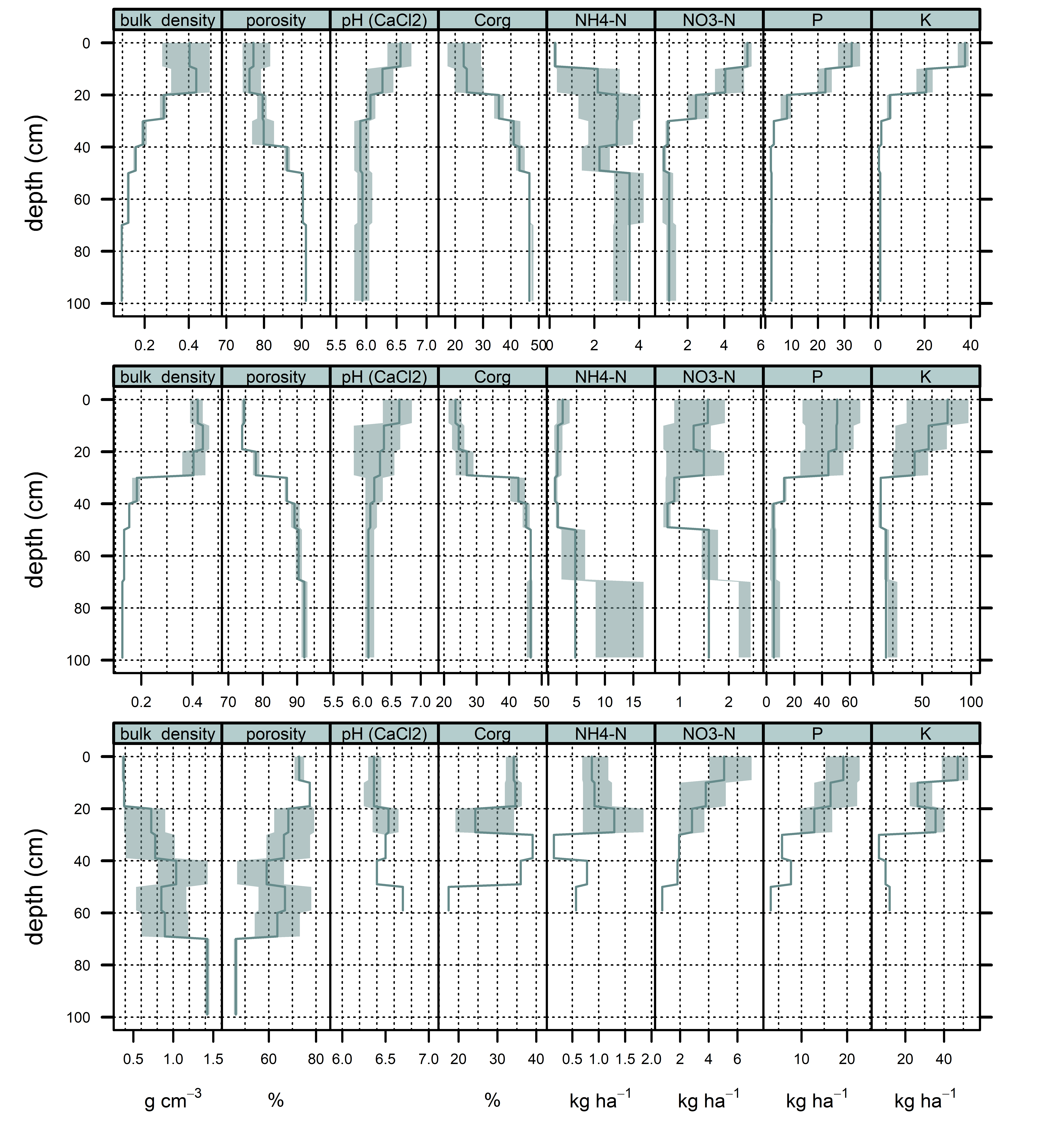


**Figure S1**. Soil depth profiles illustrating the physical and chemical parameters across the three study sites Freisinger Moos (FSM), Langenmosen (LM) and Riedhausen (RH). Lines give mean values of three soil profiles per study site excavated in 2021, with lighter colored bands indicating bounds defined by the 25th and 75th percentiles.

**Table S1**. Organic matter quality indicated by respective abundance of four C functional groups and the turnover indicator (ratio Alkyl C:O-alkyl C) of soil samples collected in profiles next to the GHG measurement treatments in the three study sites Freisinger Moos (FSM: FSM-E and FSM-F), Langenmosen (LM) and Riedhausen (RH) in 2021. Note that sampling at the site RH was confined to the organic soil layer of maximum 0.26 m depth.

| Site and soil depth | Carboxyl C | Aryl C | O-alkyl C | Alkyl C | Alkyl C/O-alkyl C ratio |
| --- | --- | --- | --- | --- | --- |
| cm | % C | % C | % C | % C |  |
| FSM 0-10 | 13.7 | 19.6 | 46.5 | 20.0 | 0.430 |
| FSM 10-20 | 13.9 | 17.9 | 44.8 | 23.3 | 0.520 |
| FSM 20-30 | 13.3 | 24.2 | 39.3 | 22.9 | 0.583 |
| FSM 30-40 | 12.4 | 26.1 | 39.4 | 21.6 | 0.548 |
| FSM 40-50 | 11.2 | 25.3 | 40.0 | 23.4 | 0.585 |
| FSM 50-70 | 13.2 | 28.7 | 37.2 | 20.5 | 0.551 |
| FSM 70-100 | 11.2 | 29.0 | 39.3 | 20.2 | 0.514 |
|  |  |  |  |  |  |
| LM 0-10 | 14.8 | 19.8 | 43.2 | 22.0 | 0.509 |
| LM 10-20 | 13.0 | 17.9 | 45.3 | 23.7 | 0.523 |
| LM 20-30 | 12.4 | 17.0 | 45.5 | 25.0 | 0.549 |
| LM 30-40 | 13.0 | 20.5 | 42.3 | 24.0 | 0.567 |
| LM 40-50 | 10.9 | 22.8 | 43.3 | 22.7 | 0.524 |
| LM 50-70 | 7.6 | 24.2 | 43.7 | 24.4 | 0.558 |
| LM 70-100 | 8.6 | 27.4 | 42.8 | 21.1 | 0.493 |
|  |  |  |  |  |  |
| RH 0-10 | 13.5 | 21.7 | 43.1 | 21.6 | 0.501 |
| RH 10-20 | 13.5 | 22.9 | 40.0 | 23.4 | 0.585 |
| RH 20-30 | 14.9 | 26.9 | 33.6 | 24.2 | 0.720 |

## S2 Experimental setup and rewetting measures

Rewetting of the FSM-F plot started in February 2017 and was achieved by installing a subsurface drip irrigation network (Uniram 16010 AS 2.3 l h^−1^, Netafim Ltd.) at 0.2 m depth, which was supplied with water by two submersible pumps (Caprari Unterwasserpumpe E6P35-6/41W) via a programmable controlling unit (Safeguard Steuerung + Pumpensteuerung FluMaster). The FSM-E basin was installed in 2016 and designed to accommodate twelve different paludiculture treatments. For precise WT regulation, the basin was divided into three adjacent 10 x 16 m sheet pile compartments. Walls were driven 2.3 m into the peat layer to simulate percolation fen conditions with slow subsurface water flow above the underlying mineral soil. Wall heights of 0.2 m above the soil surface prevented surface water and nutrient exchange between the compartments. To achieve precise WT control in the three basins, we employed a system similar to that described in Bockermann et al. (2024). This system consisted of a network of drainage tubes, central pump shafts, piezoresistive pressure transmitters (PR-26W, KELLER AG, CH), rotary and dew pumps, and a programmable controlling unit (DT85, dataTaker). As reference, a piezoresistive pressure transmitter was installed in 50-m distance in the surrounding drained field to replicate typical seasonal water level fluctuations with dampened amplitude.

To determine the optimal WT for GHG mitigation and maximized biomass yield, we established a range of WT classes across the three basin compartments, spanning from ‘moderately rewetted’ to ‘rewetted’ and ‘flooded’ conditions, with varied mean water levels of each WT class between 2020 and 2021 (Table 2 of main manuscript). Rewetting measures at the field-scale site LM involved the removal of all drainage pipes. A meandering inlet ditch connected to an uphill permanently water-filled drainage ditch enabled continuous water inflow and rewetting from August 2018 onward. Rewetting in the field-scale site RH was achieved by closing the two drainage ditches on the site with small plastic sheet pile weirs in March 2019. In April 2021, two wells were drilled to supplement rewetting with groundwater from the gravel aquifer, pumped into the dammed ditches via solar-powered pumps. Achieved WTs for all treatments are given in Table 2 (main manuscript). A WT below the ground surface is defined as negative and flooding as positive. The experimental setup at all field-scale sites further included the permanent installation of boardwalks to prevent soil disturbance during measurements. For chamber GHG flux measurements we permanently installed soil collars (PVC) in clusters of three spatial replicates per paludiculture treatment. At each treatment, we measured soil temperatures (109 Thermistor Probe, Campbell Scientific, UK) at −0.02 m (ST2), −0.05 m (ST5), and −0.10 m (ST10), air temperatures (CS215 sensors with unaspirated radiation shields, Campbell Scientific, UK) at +0.20 m (Tair), and groundwater levels (PVC dip wells, 6 cm diameter, 1 m length, Brunnenfilter DN 50, 2", logger Rugged TROLL 100, In-Situ Inc., US). Each site was further equipped with two photosynthetically active radiation (PAR) sensors (SKP215 Quantum Sensor, Campbell Scientific, UK) at 2 m height. PAR values were logged as 30-min averages of 1-min measurements while all other values were measured and logged in 30-min intervals (data logger CR300 at field-scale sites and CR1000 at FSM-E (one per basin), Campbell Scientific, UK).

## S3 Greenhouse gas measurements

### Manual greenhouse gas flux measurements

Manual measurements of CO_2_ were carried out three-weekly as full-day campaigns using transparent (net ecosystem exchange, NEE) and white opaque (ecosystem respiration, Reco) non-steady state chambers (Livingston and Hutchinson, 1995; Drösler, 2005). Chamber dimensions were 0.78 x 0.78 x 0.5 m with added extensions of the same dimensions to accommodate plant growth (max. chamber heights of 2 m at LM for NC). Soil collars had inside dimensions of 0.75 × 0.75 m with blade depths of 0.2 m resulting in an area of 0.5625 m² for GHG exchange detection per treatment replicate. Details on chamber design and measurement procedure can be found in Bockermann et al. (2024). In this study, we measured CO_2_ and H_2_O concentrations using infrared gas analyzers (IRGA; LI-840 and LI-850, LI-COR, USA) and logging intervals of 1 s (GP2 Data logger, Delta-T Devices, UK). We conducted a total of 19, 18 (19 in RCG to cover pre-harvest conditions in June 2021) and 17 CO2 campaigns at FSM-F (2019), LM (2021) and RH (2021), respectively. Manual CO_2_ campaigns covered a minimum of four measurements per replicate in winter and a maximum of 25 measurements per replicate in summer.

Manual measurements of CH_4_ and N_2_O fluxes were conducted using opaque chambers and extensions (same dimensions as for CO_2_) in seven-day intervals between 9 and 11 am wintertime when mean soil temperatures at −0.02 m depth prevail. CH_4_ and N_2_O concentrations at FSM-F were measured directly in the field by connecting six opaque chambers at a time (two treatments with three replicates each) to a 16-Port Distribution Manifold (A0311, Picarro Inc., USA) for dynamic sequential headspace gas sampling between the chambers. The headspace concentration of each replicate was determined online at equal intervals six to nine times for a period of 50 sec each over a 60 to 120-min closure time (depending on chamber height) by cavity ring-down spectrometry (CRDS; G2308, Picarro Inc., USA). Single gas concentrations for each sampling interval were calculated as the 30-sec average of measured concentrations at 1.2 Hz, excluding the initial sampling lag phase (first 1-20 sec) due to tube length.

CH_4_ and N_2_O fluxes in LM and RH were determined from overpressurized headspace gas samples (approx. 28 ml) collected in 20-ml glass vials in the field (five samples taken at equal intervals over a 30 to 90-min closure time) using gas chromatography (Clarus 480 GC, PerkinElmer, USA) in the laboratory. Further details on manual measurement protocols can be found in Eickenscheidt et al. (2015) and Bockermann et al. (2024). We conducted a total of 52, 55 and 55 weekly CH_4_ and N_2_O measurements in the field sites FSM-F (2019), LM (2021) and RH (2021), respectively.

### Automated greenhouse gas flux measurements

A fully automatic robotic chamber measurement system (ARC) was installed above the FSM-E basin setup. ARC was developed during 2016 to 2019 by the Peatland Science Centre (PSC, University of Applied Sciences Weihenstephan-Triesdorf, Germany) and the company Umwelt Geräte Technik GmbH (UGT, Germany). Four pairs of opaque and transparent automatic chambers on rails allow for daily alternating measurements of CO_2_ (NEE and Reco) and combined measurements of CH_4_ and N_2_O of twelve treatments. Chamber dimensions are 1.0 x 1.0 m at the base with heights of 1.0 m for *Carex*, 1.5 m for RCG, and up to 2.5 m for CR and BC to accommodate maximum plant growth. Soil collars (n=36) with silicon bottom sealing to achieve gas-tightness have inside dimensions of 1.0 × 1.0 m with blade depths of 0.16 m resulting in an area of 1.0 m² for GHG exchange detection per treatment replicate. Chambers are equipped with inside air temperature sensors (type MQ 0008, tmg, Germany), PAR sensors (Quantum Sensor EMS 12, EMS, Czech Republic, transparent chambers only), pressure equilibration valves, ventilators (12-fold mixing of chamber volume per minute). Each chamber pair is equipped with an IRGA analyzer (LI-840, LICOR Biosciences, USA) for the continuous detection of CO_2_ and H_2_O concentrations. CO_2_ measurements alternated between NEE and Reco with closure times of 3 min during the vegetation period of April to mid Oct and 5 min otherwise. For CH_4_ and N_2_O concentration detection, the four opaque chambers are connected to a 16-Port Distribution Manifold (A0311, Picarro Inc., USA) and a CRDS analyzer (G2308, Picarro, USA). During a 45-min chamber closure time, the headspace concentration of each chamber was determined online at equal intervals ten times for a period of 60 sec each. Single gas concentrations for each sampling interval were calculated as the 30-sec average of measured concentrations at 1.2 Hz, excluding the initial sampling lag phase (first 1-30 sec) due to tube length. All data including further ancillary measurements of outside air pressure, PAR and wind speed were recorded in 1-second intervals (Simens SPS SIMATIC S7-1200) and transferred hourly to a database on a network server via a cellular communication system. ARC does not operate at 30-min mean wind speed > 4 m s^−1^ or 3-min mean wind speed > 6 m s^−1^. The final dataset consists of 60,145 single NEE fluxes, 63,322 single Reco fluxes, 22,715 single CH_4_ fluxes and 22,715 single N_2_O fluxes over the 24-months measurement period.

### Greenhouse gas flux calculations and modelling

Gas flux calculations and modelling were done in R (R Core Team 2022). We calculated CO_2_ fluxes by linear regression over a moving window of each flux measurement dataset including a minimum of 31 data points (sec 0-30) for net ecosystem exchange (NEE) and 61 data points (sec 0-60) for ecosystem respiration (Reco), using the R package *flux* (version 0.3-0.1, Jurasinski et al., 2014). We adjusted model selection to prioritizing the model with the steepest slope, provided all necessary linear regression assumptions were met (α = 0.05), rather than choosing the model with the highest R² (cf. Bockermann et al., 2024). We modelled CO_2_ fluxes (Reco and GPP) using a campaign- and treatment-based approach for manually determined fluxes (e.g., Eickenscheidt et al., 2015, Tiemeyer et al., 2024). For high-frequency flux data determined with ARC, we used weekly aggregated fluxes per replicate as the model basis. While models for the campaign-based approach correspond to the specific measurement date, the weekly aggregated flux models were assigned to the first day of the respective measurement week.

We modelled the dependency between the measured R_eco_ fluxes and temperature (Tair, ST2, ST5 or ST10) as the explanatory variable according to Lloyd and Taylor (1994) (Eq. 1):

$Reco = Rref \times e^{E0} \times((1/Tref - T0) - 1/T - T0)$ (1)

where Reco is the ecosystem respiration in µmol CO_2_ m^−2^ s^−1^, Rref is the respiration at the reference temperature in µmol CO_2_ m^−2^ s^−1^, E0 is the activation energy in K, Tref is the reference temperature 283.15 K, T0 is the temperature constant for the start of biological processes: 227.13 K, and T is air or soil temperature in K.

Gross primary productivity (GPP) values were calculated by subtracting the temporally corresponding modelled Reco flux from the measured NEE flux. Subsequently, GPP for each treatment/replicate was modeled using campaign/week-derived fluxes, with PAR as the explanatory variable, following the Michaelis–Menten type rectangular hyperbolic function as proposed by Falge et al. (2001):

$GPP = \alpha\times PAR/(1-(PAR/2000) + (\alpha\times PAR/GPP2000))$ (2)

where GPP is gross primary production in µmol CO_2_ m^−2^ s^−1^, α is the initial slope of the curve or light use efficiency in µmol CO_2_ m^−2^ s^−1^/μmol m^−2^ s^−1^, the photon flux density of PAR is given in μmol m^−2^ s^−1^, and GPP2000 is the GPP at PAR 2000 μmol m^−2^ s^−1^ in µmol CO_2_ m^−2^ s^−1^.

Regarding model evaluation, we evaluated campaign models of Reco and GPP as well as the resulting annual Reco, GPP and NEE balances using Pearson's Correlation Coefficient (Pearson’s *r*) indicating strength and direction of the linear relationship between measured and modelled values, Nash-Sutcliffe Efficiency (NSE) indicating the goodness of fit of the models (Nash and Sutcliffe, 1970), Percent Bias (PBIAS) representing model accuracy and systematic bias, i.e., under- or overestimation, Root Mean Square Error (RMSE) giving the standard deviation of the model prediction error, Normalized Root Mean Square Error (NRMSE) expressing model performance relative to the range of observed values, and Root Mean Square Error to Standard Deviation Ratio (RSR) giving the ratio of the root mean square error to the standard deviation of measured data (Moriasi et al., 2007; Eickenscheidt et al., 2015). See model parameter overview in **Figure S4** and full parameter tables in repository **Table R1** (Bockermann et al., 2025).

Consecutive campaigns/weeks were pooled if no statistically significant relationship was found between temperature and Reco fluxes or PAR and GPP fluxes (during wintertime) within an individual campaign/week. A mean CO_2_ flux was calculated for measurement campaigns/weeks during snow cover or when pooling did not result in a statistically significant relationship between explanatory variable and the pooled CO_2_ fluxes.

Half-hourly Reco or GPP fluxes were derived using the model parameters Rref and E0 or α and GPP2000, respectively, and the time series of measured air or soil temperatures and PAR.

For fluxes between consecutive campaigns/weeks, the parameters from the first campaign/week were used to model forward, and those from the second campaign to model backward. Then, 0.5-hourly values were obtained as the distance-weighted average of both fluxes (cf. Tiemeyer et al., 2024). During periods of harvest or snow cover, model parameters were kept constant up to the event, after which parameters from the subsequent campaign were applied (Eickenscheidt et al., 2015). NEE fluxes were calculated 0.5-hourly by summing Reco and GPP at each time step. Annual balances of Reco, GPP, and NEE were derived by summing the modeled 0.5-hourly Reco, GPP and NEE fluxes, respectively. See **Table S3** for model evaluation metrics as described for campaign-specific model evaluation.

Uncertainties in the annual balances of Reco, GPP, and NEE were quantified by calculating annual sums using the upper and lower bounds of the estimated parameters (Rref, E0, α, GPP2000), based on their standard errors (Drösler, 2005; Elsgaard et al., 2012; Eickenscheidt et al., 2015). This approach accounts for the potential inaccuracies in the fitted Reco and GPP response functions, however, uncertainties arising from the interpolation between consecutive measurement campaigns, which may introduce additional, unquantified error, cannot be captured (cf. Heller et al., 2025).

Due to leakage issues in the LI-840 analyzer between January 1 and April 24, 2020, the campaign-based modeling of Reco and GPP, based on the Lloyd & Taylor and Michaelis-Menten functions, could not be applied for the *Typha* treatments at FSM-E during this period (see Figure S2b). A similar limitation affected the campaign-based GPP modeling for the *Phragmites* treatments at FSM-E between April 4 and July 7, 2020, caused by a malfunctioning switching valve (see Figure S2b). To gap-fill these specific periods, we employed plant species-specific GAMMs using the 'mgcv' R package (v1.9.1; Wood, 2023). These models were calibrated using all available measured Reco or calculated GPP fluxes from the respective treatment over the two subsequent years of observations. In general we followed the steps described in section 2.5 of the main manuscript. As ecological drivers for Reco or GPP fluxes we used: air or soil temperatures; WT starting from 0.5-hour measurement intervals and aggregated to various temporal scales up to a maximum of 14-day means; PAR; vegetation indices such as the normalized difference vegetation index (NDVI) and the leaf area index (LAI). For each response variable (Reco *Typha* FSM-E, GPP *Typha* FSM-E, and Reco *Phragmites* FSM-E) the model formulation began with a saturated model followed by backward selection through dropping non-significant terms one at a time based on p-values of an applied likelihood ratio test. Because consecutive flux measurements from the same replicate were not independent (i.e., temporal pseudoreplication), we accounted for this by including 'replicate' as a random effect in the model structures. In addition, to address heteroscedasticity observed in the residuals, we incorporated a variance function into the models. The final formula for Reco was CO_2_ flux ~ te(Tair, WT, NDVI, bs = c("cs", "cs", "cs"), random=list(replicate =~1), weights = varIdent(form=~1|Month)) (R^2^adj. = 0.858). Regardless of plant species (*Typha* or *Phragmites*), the final model structure for GPP was as follows: CO_2_ flux ~ te(PAR, NDVI, LAI, bs = c("cs", "cs", "cs"), random=list(replicate =~1), weights = varIdent(form=~1|Month)) (*Typha* FSM-E: R^2^adj. = 0.934; *Phragmites* FSM-E: R^2^adj. = 0.946). Half-hourly Reco or GPP fluxes were estimated using the 'predict' function of the 'mgcv' R package (v1.9.1; Wood, 2023), based on the continuous time series of the respective measured covariates for the specified periods.

Gas fluxes of CH_4_ and N_2_O were calculated by robust linear or nonlinear Hutchinson-Mosier regression (HMR, diffusion-based) using the R package *gasfluxes* (version 0.4, Fuß et al., 2020). For further details on the CH_4_ and N_2_O flux calculation procedures see Bockermann et al. (2024). Annual, replicate-specific gas balances were calculated by linearly interpolating the measured fluxes. For field-scale sites, these balances were then averaged across the three replicates to obtain treatment-specific annual gas balances, reported with standard error (SE).

In line with the current national emission reporting protocol for organic soils, we calculate ‘CO_2_-C_onsite_’ values in t C ha^−1^ yr^−1^, which comprise the NEE C balance and harvest C export (Tiemeyer et al., 2020). For the three fertilized RCG treatments we also included fertilizer C import in the ‘CO_2_-C_onsite_’ values. To arrive at ‘CO_2_-C_organic_’ (Tiemeyer et al., 2020, eq. 1), off-site emissions from DOC leaching are included by adding the IPCC EF of 0.31 (for drained sites) and 0.24 t C ha^−1^ yr^−1^ (for rewetted sites) (IPCC, 2014). Methane emissions are reported as ‘CH_4 organic_’, which include CH_4_ emissions from the land itself (‘CH_4 land_’) as well as emissions from open ditches times their respective areal fractions (IPCC, 2014; Tiemeyer et al., 2020). The term is simplified for rewetted areas as ditches are assumed to emit similar amounts of CH_4_ as the rewetted land (‘CH_4 organic_’ = ‘CH_4 land_’). We added CH_4_ emissions from drainage ditches in annual balances of treatments with WT < −0.1 m by including the IPCC default EF (‘CH_4 ditch_’) of 527 kg CH_4_ ha^−1^ yr^−1^ (for shallow-drained grassland, IPCC, 2014) multiplied by the national areal default ditch fraction of 0.013 (Tiemeyer et al., 2020). In line with Tiemeyer et al. (2020) we report N_2_O emissions for all areas including rewetted areas as direct N_2_O emissions in kg N_2_O-N ha^−1^ yr^−1^.

**
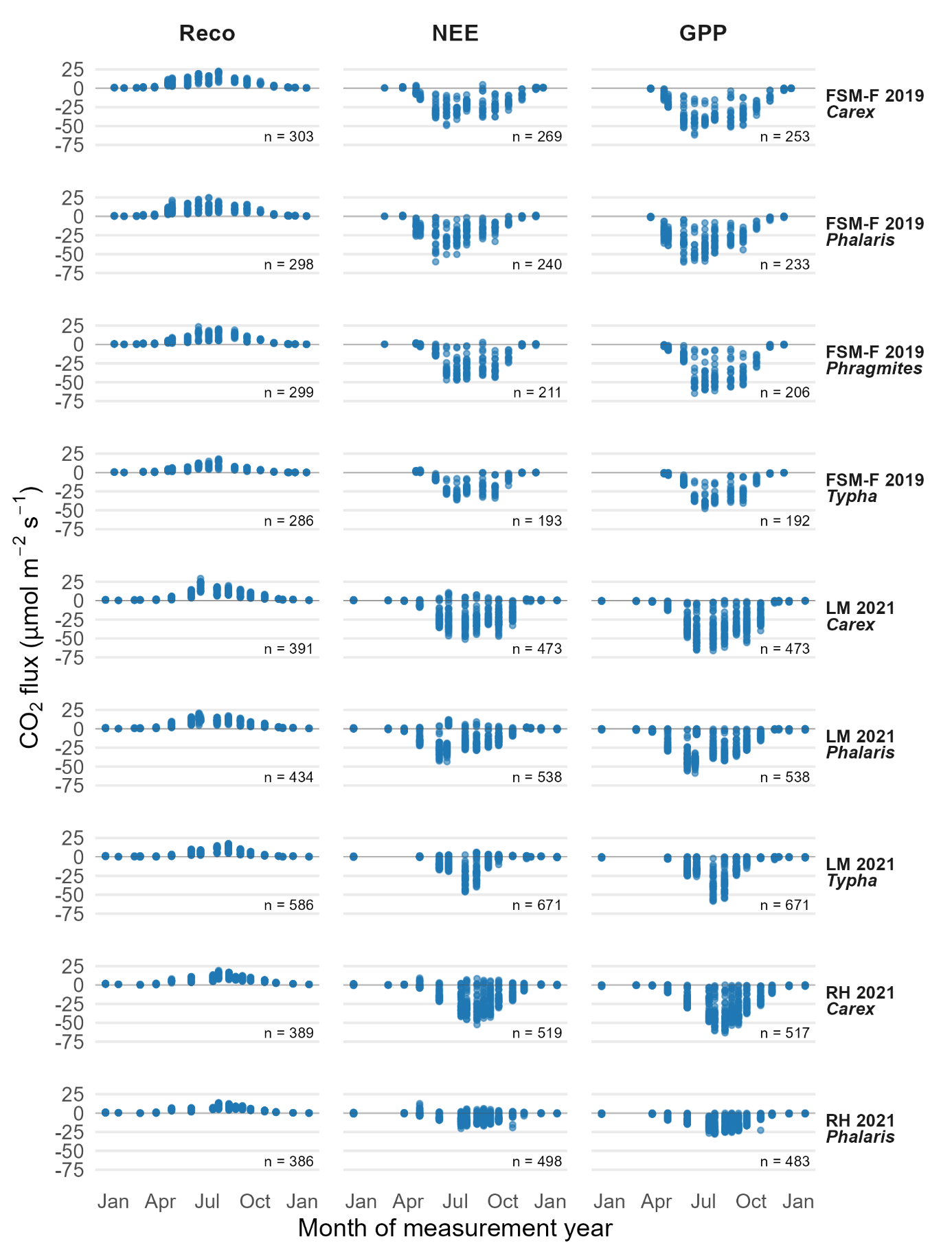
**

**Figure S2a**. Time series of manually measured CO_2_ ecosystem respiration (Reco in µmol CO_2_ m^−2^ s^−1^) and net ecosystem exchange (NEE in µmol CO_2_ m^−2^ s^−1^) fluxes, as well as calculated gross primary production (GPP in µmol CO_2_ m^−2^ s^−1^) fluxes for all tested paludicultures during the greenhouse gas measurement campaigns in 2019 at the field-scale sites Freisinger Moos (FSM-F), and in 2021 for Langenmosen (LM) and Riedhausen (RH). Data are differentiated by site and paludiculture plant species. For the nine manually measured field-scale treatments, CO_2_ fluxes of the three replicates were aggregated for campaign-specific modelling.

**
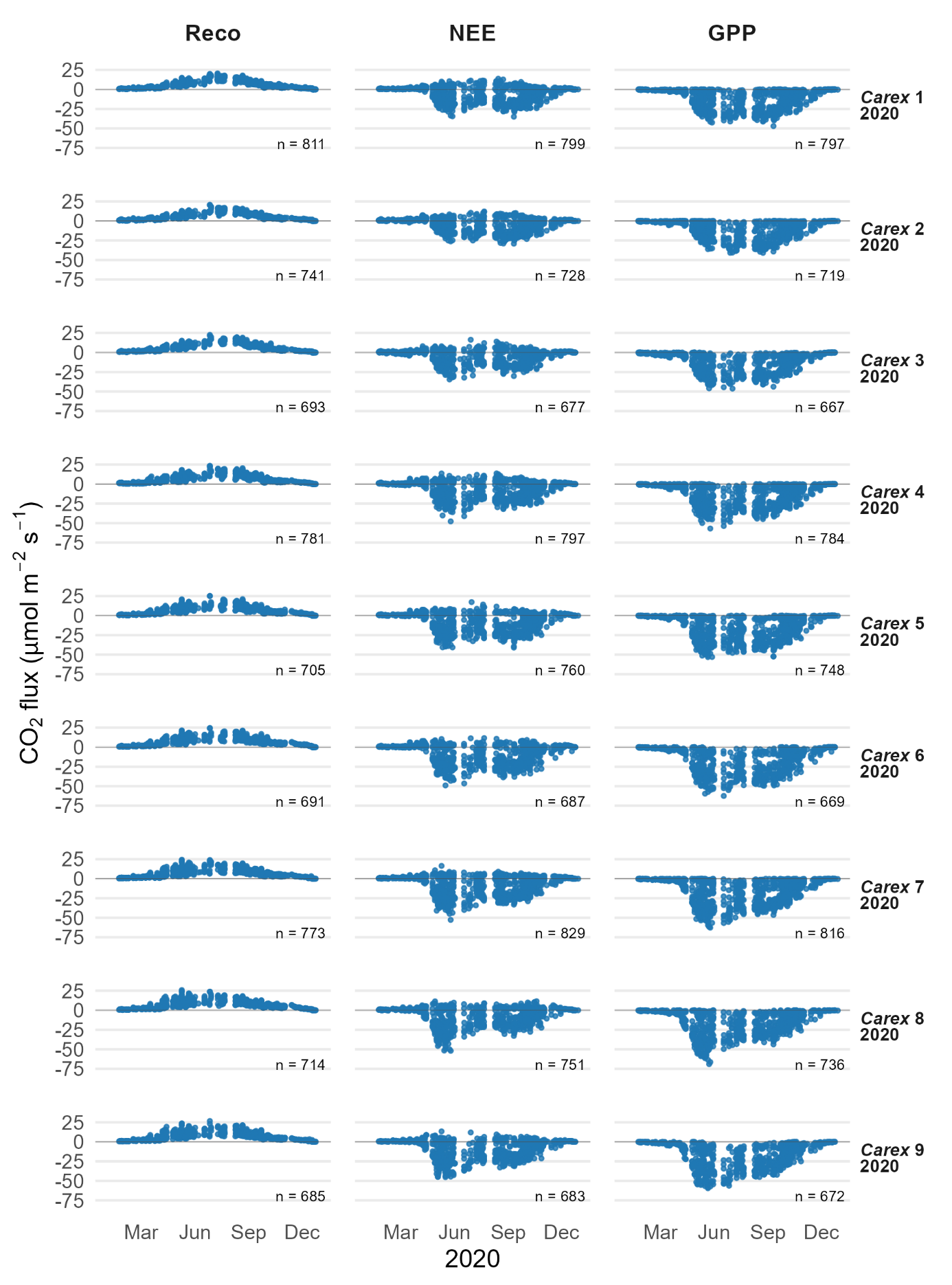
**

**Figure S2b**. Time series of automatically (ARC) measured CO_2_ ecosystem respiration (Reco in µmol CO_2_ m^−2^ s^−1^) and net ecosystem exchange (NEE in µmol CO_2_ m^−2^ s^−1^) fluxes, as well as calculated gross primary production (GPP in µmol CO_2_ m^−2^ s^−1^) fluxes for all tested paludicultures during the greenhouse gas measurement campaigns in 2020 and 2021 at the site Freisinger Moos (FSM-E). All ARC measured fluxes are shown per replicate, as the high temporal resolution allowed flux models to be developed for each replicate separately. This page: FSM-E Carex 2020.

**Figure S2b. continued**: Time series of automatically (ARC) measured CO_2_ ecosystem respiration (Reco in µmol CO_2_ m^−2^ s^−1^) and net ecosystem exchange (NEE in µmol CO_2_ m^−2^ s^−1^) fluxes, as well as calculated gross primary production (GPP in µmol CO_2_ m^−2^ s^−1^) fluxes for FSM-E *Phalaris* 2020.

***
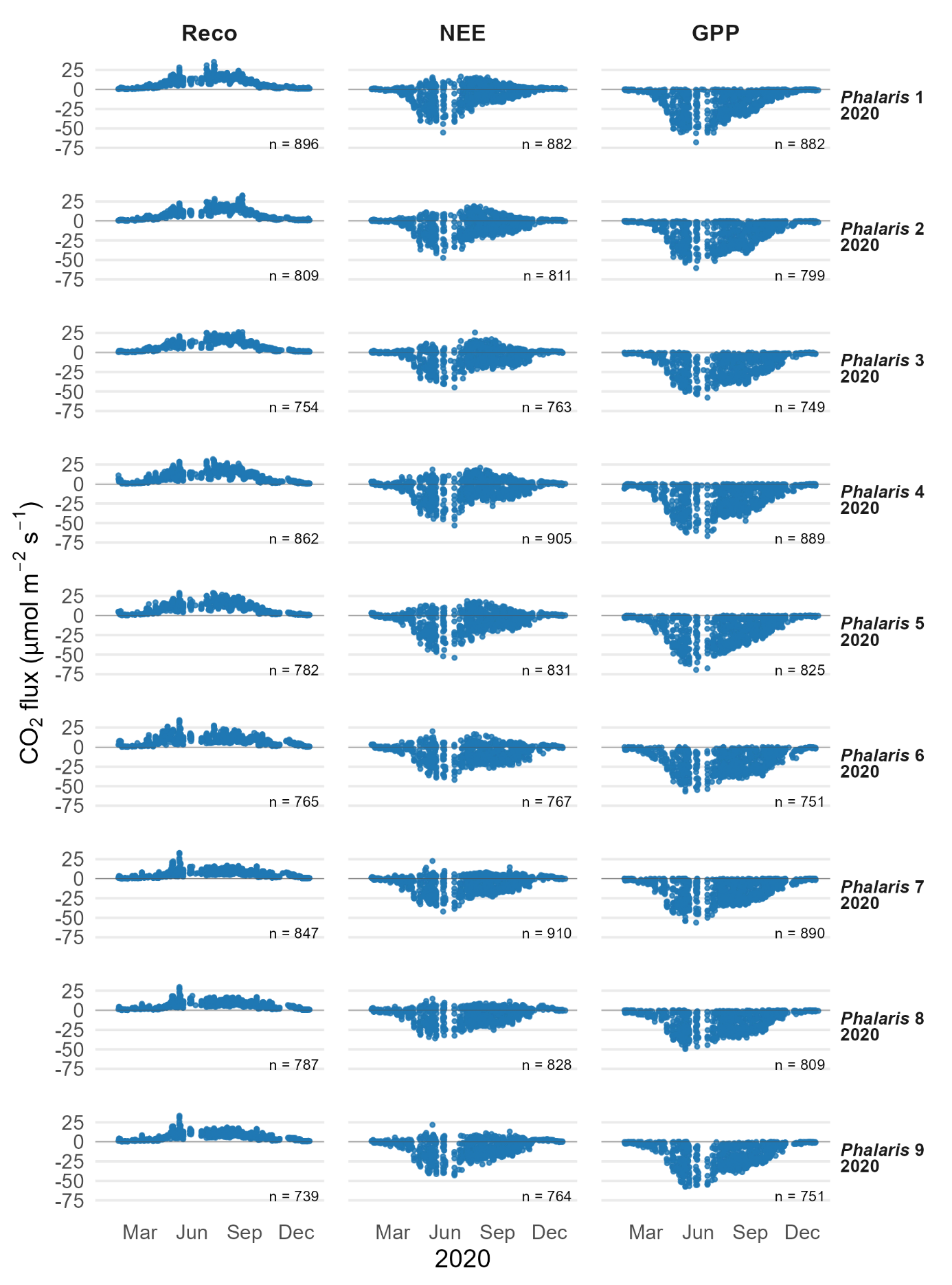
***

**Figure S2b. continued**: Time series of automatically (ARC) measured CO_2_ ecosystem respiration (Reco in µmol CO_2_ m^−2^ s^−1^) and net ecosystem exchange (NEE in µmol CO_2_ m^−2^ s^−1^) fluxes, as well as calculated gross primary production (GPP in µmol CO_2_ m^−2^ s^−1^) fluxes for FSM-E *Phragmites* 2020.

***
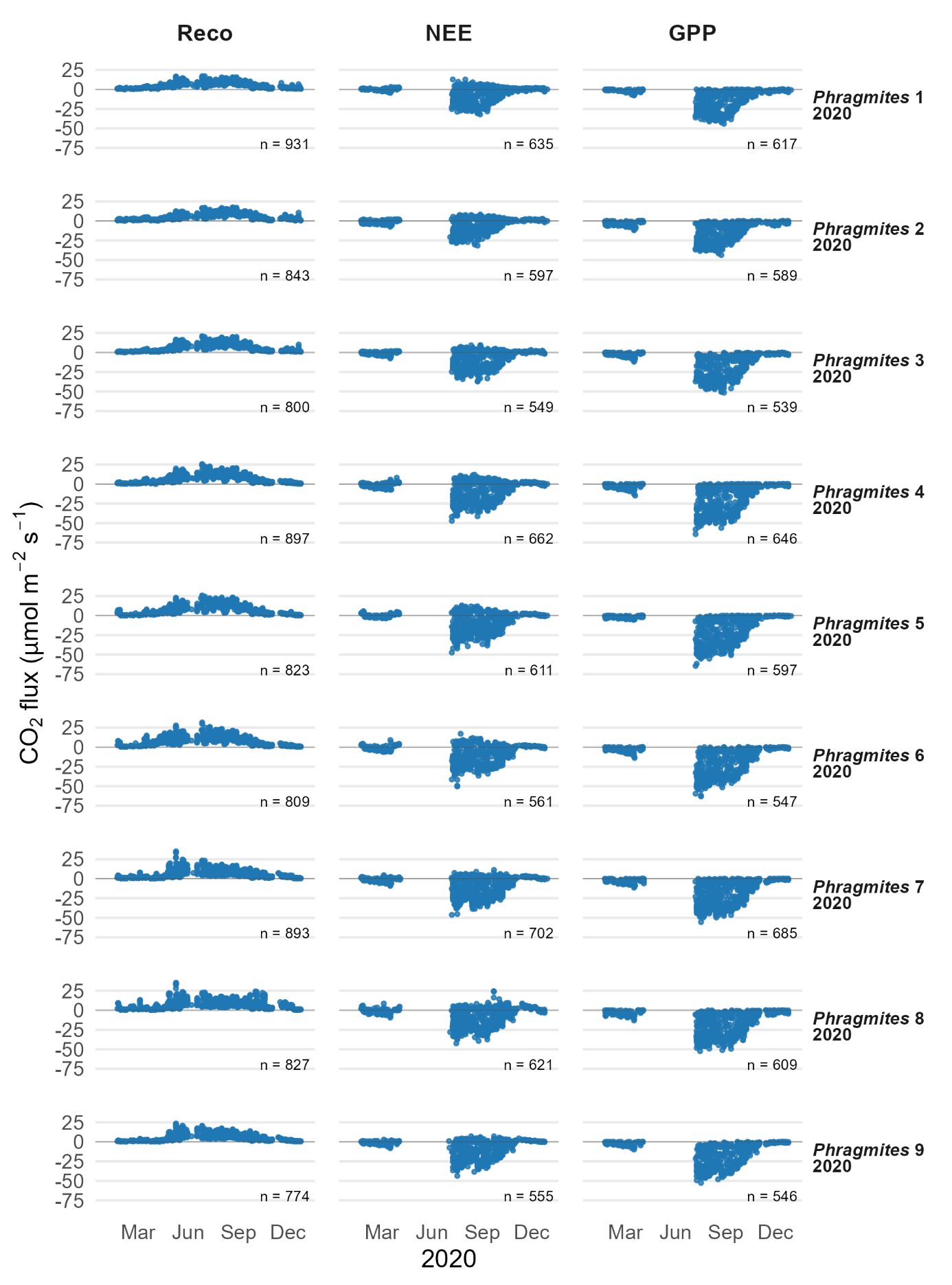
***

**Figure S2b. continued**: Time series of automatically (ARC) measured CO_2_ ecosystem respiration (Reco in µmol CO_2_ m^−2^ s^−1^) and net ecosystem exchange (NEE in µmol CO_2_ m^−2^ s^−1^) fluxes, as well as calculated gross primary production (GPP in µmol CO_2_ m^−2^ s^−1^) fluxes for FSM-E *Typha* 2020.

***
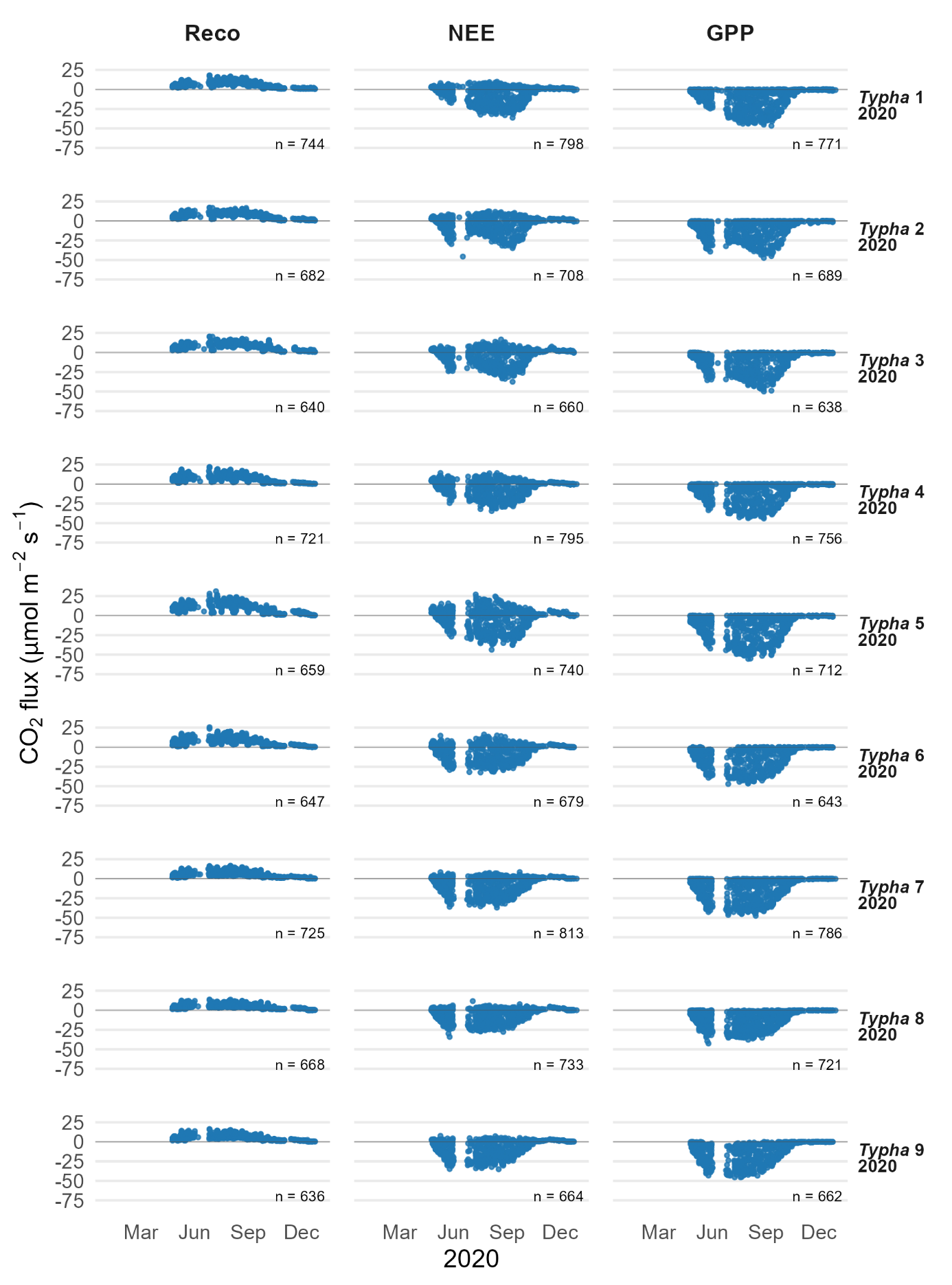
***

**Figure S2b. continued**: Time series of automatically (ARC) measured CO_2_ ecosystem respiration (Reco in µmol CO_2_ m^−2^ s^−1^) and net ecosystem exchange (NEE in µmol CO_2_ m^−2^ s^−1^) fluxes, as well as calculated gross primary production (GPP in µmol CO_2_ m^−2^ s^−1^) fluxes for FSM-E *Carex* 2021.

***
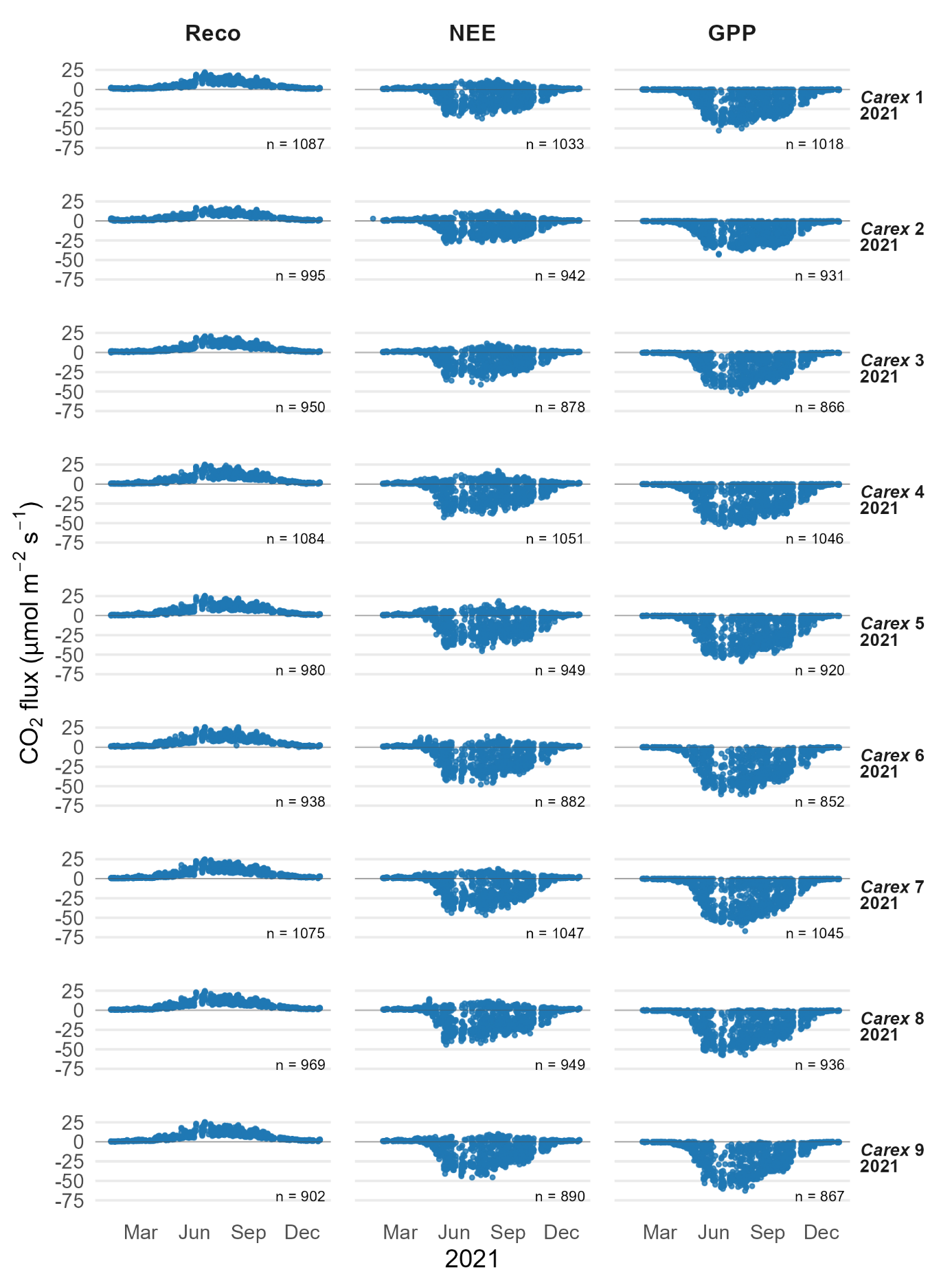
***

**Figure S2b. continued**: Time series of automatically (ARC) measured CO_2_ ecosystem respiration (Reco in µmol CO_2_ m^−2^ s^−1^) and net ecosystem exchange (NEE in µmol CO_2_ m^−2^ s^−1^) fluxes, as well as calculated gross primary production (GPP in µmol CO_2_ m^−2^ s^−1^) fluxes for FSM-E *Phalaris* 2021.

***
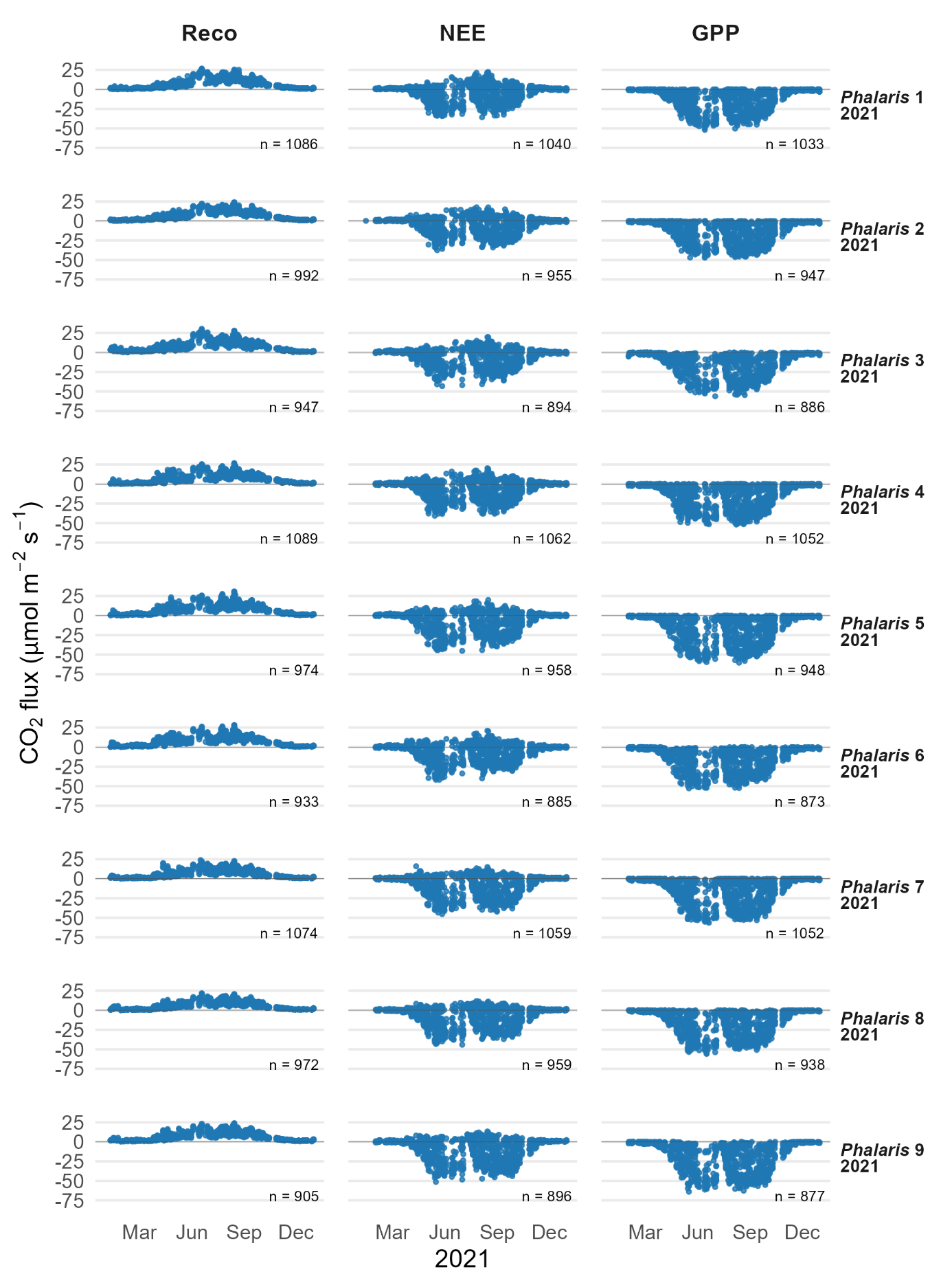
***

**Figure S2b. continued**: Time series of automatically (ARC) measured CO_2_ ecosystem respiration (Reco in µmol CO_2_ m^−2^ s^−1^) and net ecosystem exchange (NEE in µmol CO_2_ m^−2^ s^−1^) fluxes, as well as calculated gross primary production (GPP in µmol CO_2_ m^−2^ s^−1^) fluxes for FSM-E *Phragmites* 2021.

***
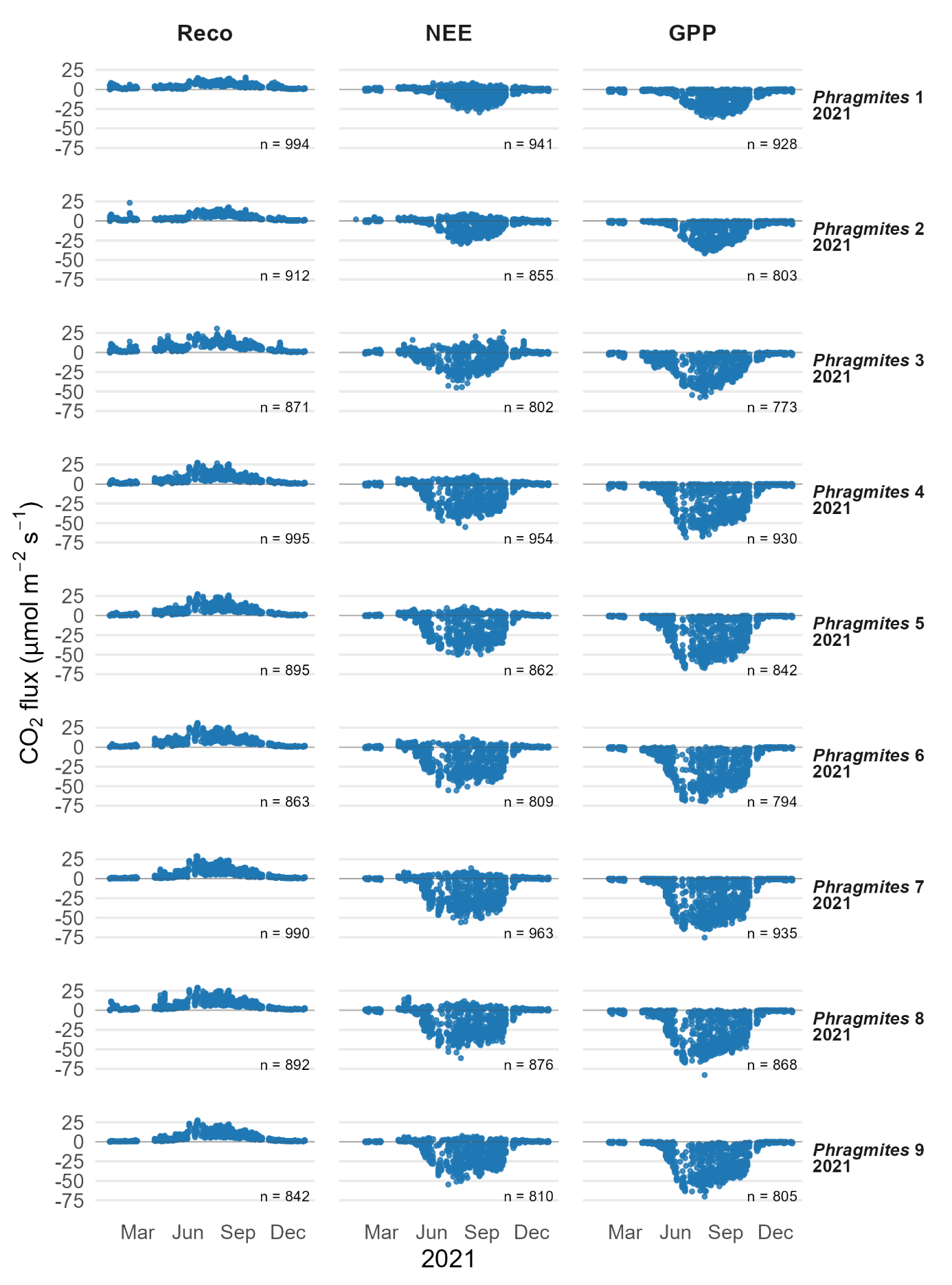
***

**Figure S2b. continued**: Time series of automatically (ARC) measured CO_2_ ecosystem respiration (Reco in µmol CO_2_ m^−2^ s^−1^) and net ecosystem exchange (NEE in µmol CO_2_ m^−2^ s^−1^) fluxes, as well as calculated gross primary production (GPP in µmol CO_2_ m^−2^ s^−1^) fluxes at the field-scale site FSM-E *Typha* 2021.

***
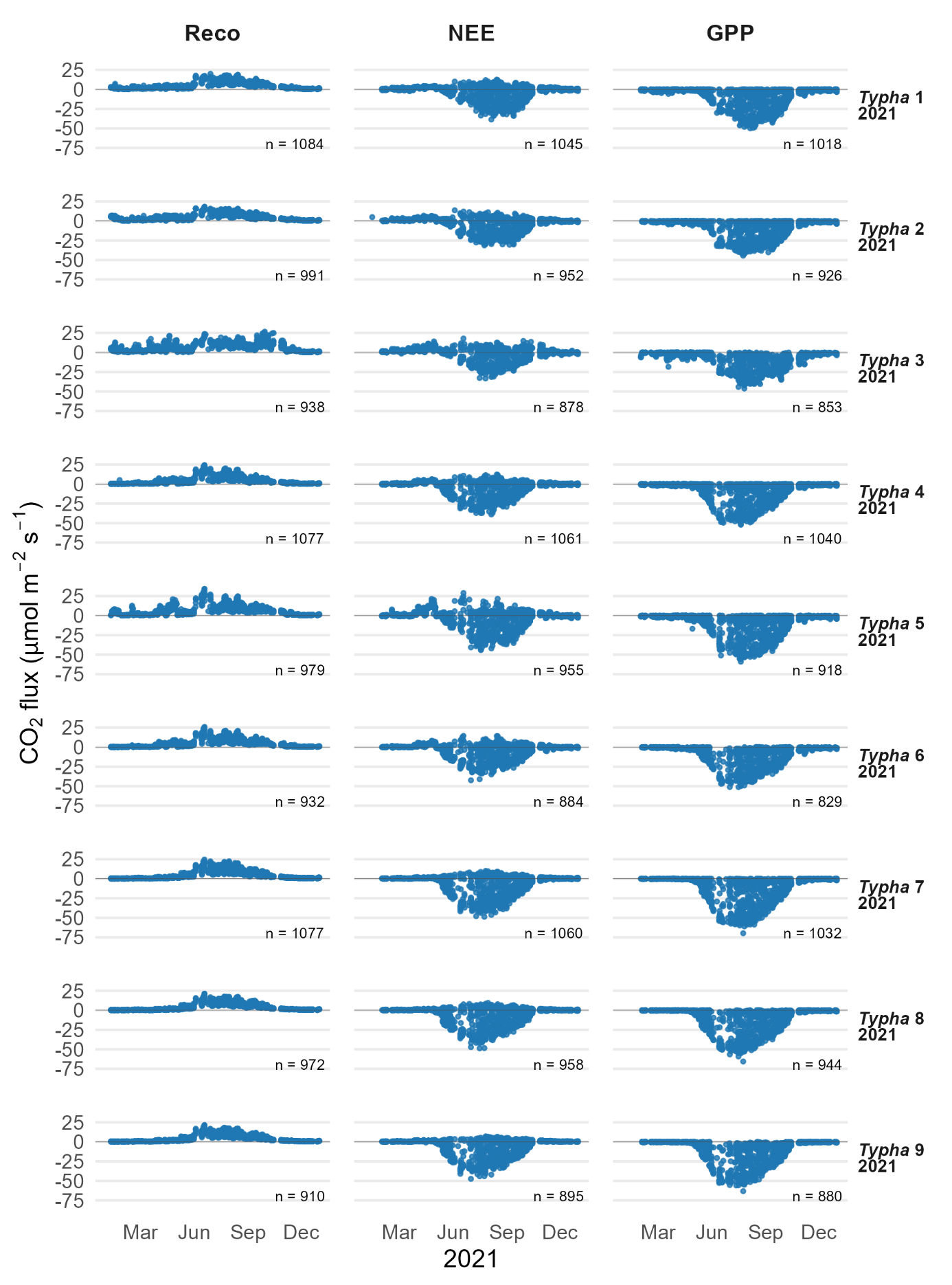
***


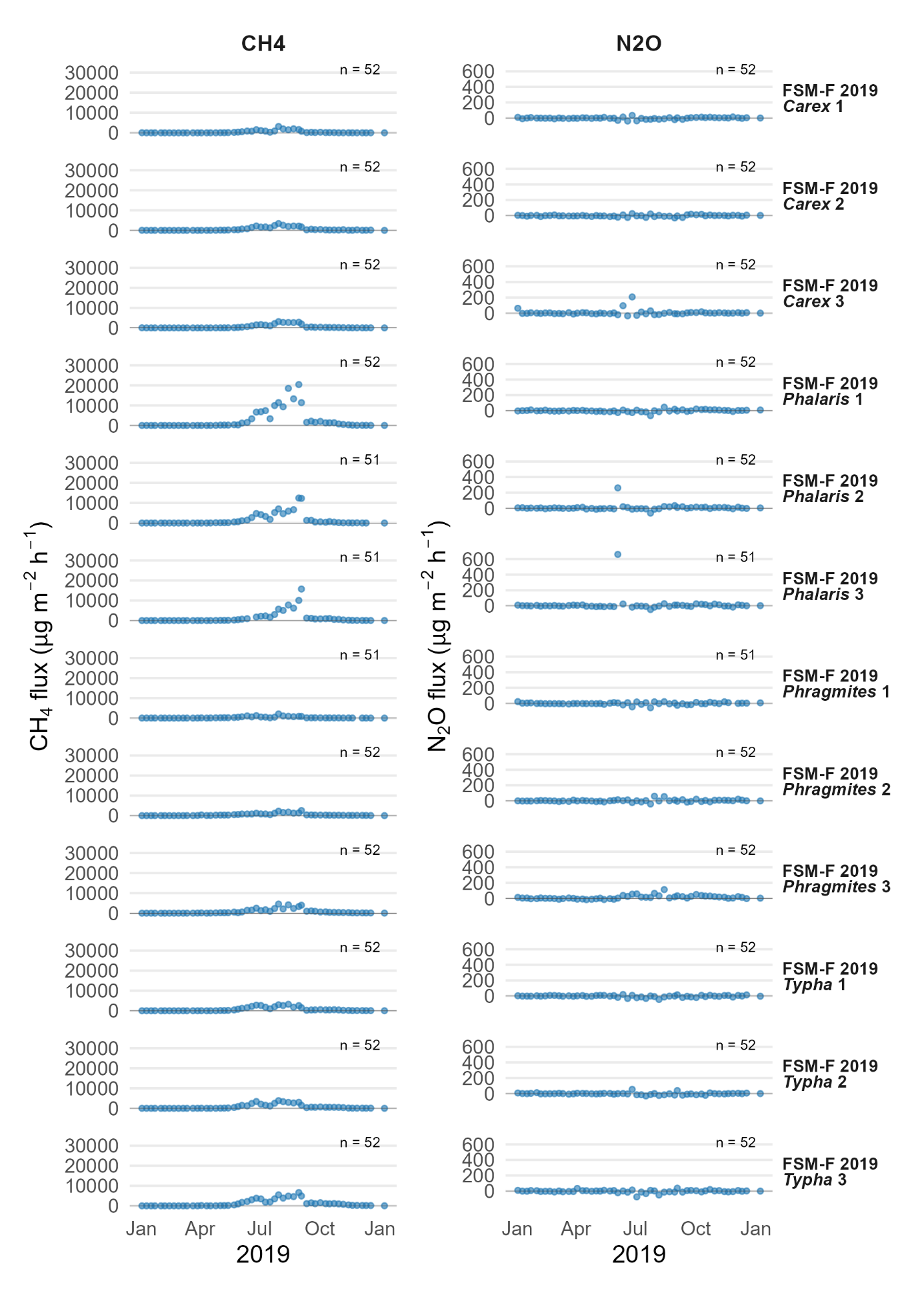


**Figure S3a.** Time series of manually measured methane (µg CH_4_ m^−2^ h^−1^) and nitrous oxide (µg N_2_O m^−2^ h^−1^) fluxes are shown for all replicates of the tested paludicultures during the greenhouse gas measurement campaigns in 2019 and 2021 at the field-scale sites Freisinger Moos (FSM-F), Langenmosen (LM), and Riedhausen (RH), differentiated by site and paludiculture plant species for each replicate (1-3). This page: Carex, Phalaris, Phragmites and Typha treatment replicates at the field-scale site FSM-F 2019.

**Figure S3a. continued:** Time series of manually measured methane (µg CH_4_ m^−2^ h^−1^) and nitrous oxide (µg N_2_O m^−2^ h^−1^) fluxes of the *Carex*, *Phalaris* and *Typha* treatment replicates at the field-scale site Langenmosen (LM) in 2021.


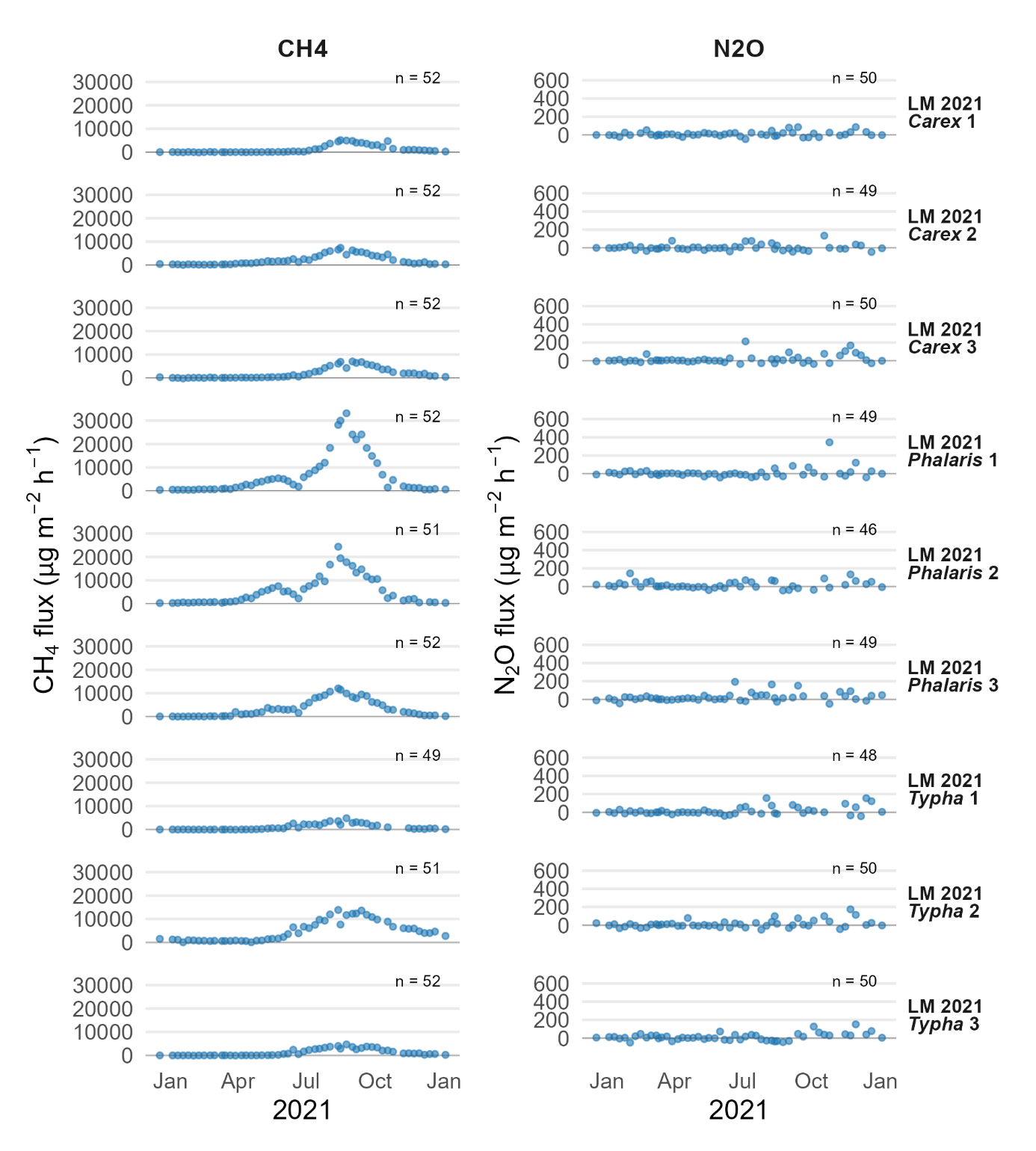


**Figure S3a. continued:** Time series of manually measured methane (µg CH_4_ m^−2^ h^−1^) and nitrous oxide (µg N_2_O m^−2^ h^−1^) fluxes of the *Carex* and *Phalaris* treatment replicates at the field-scale site Riedhausen (RH) in 2021.


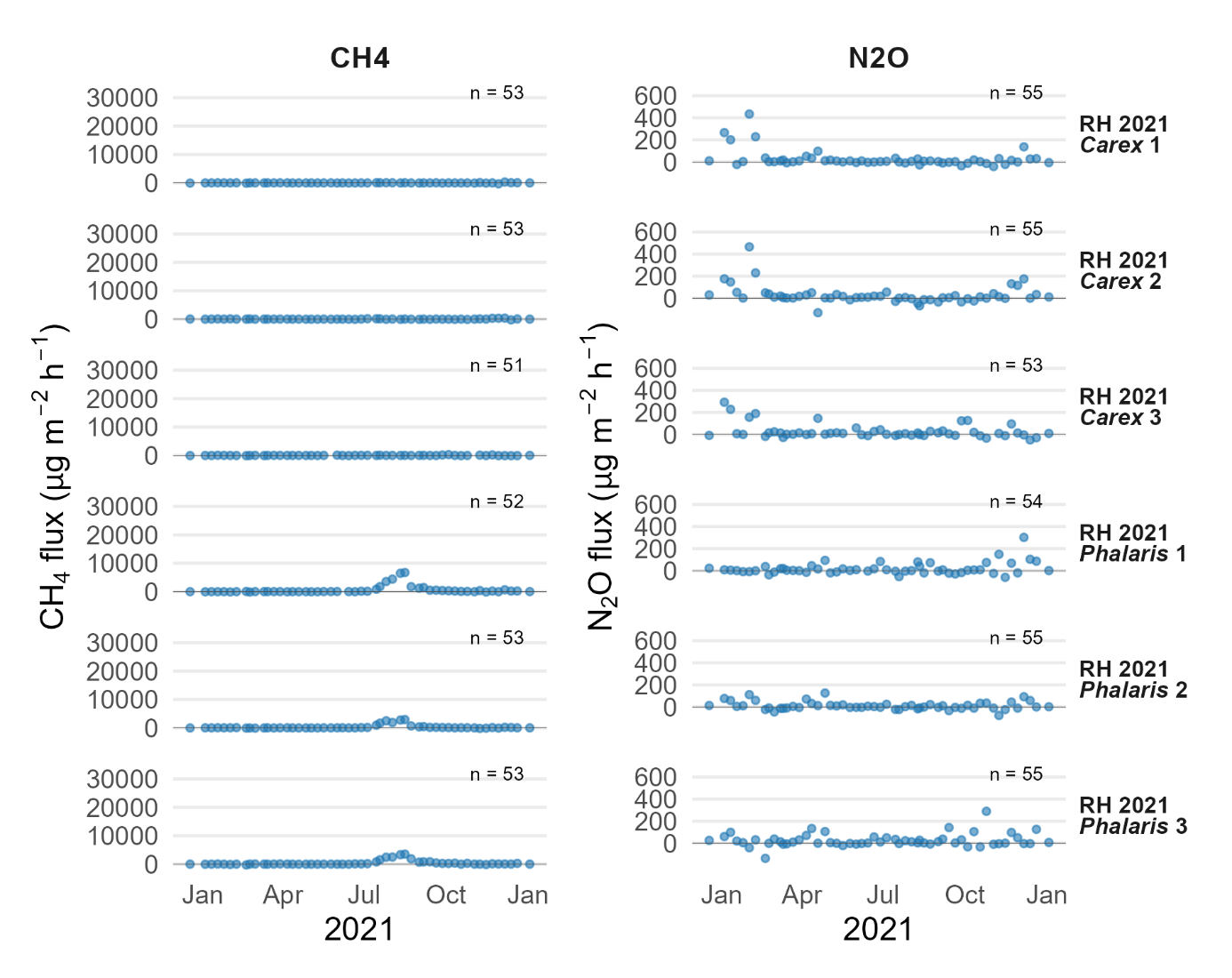


**
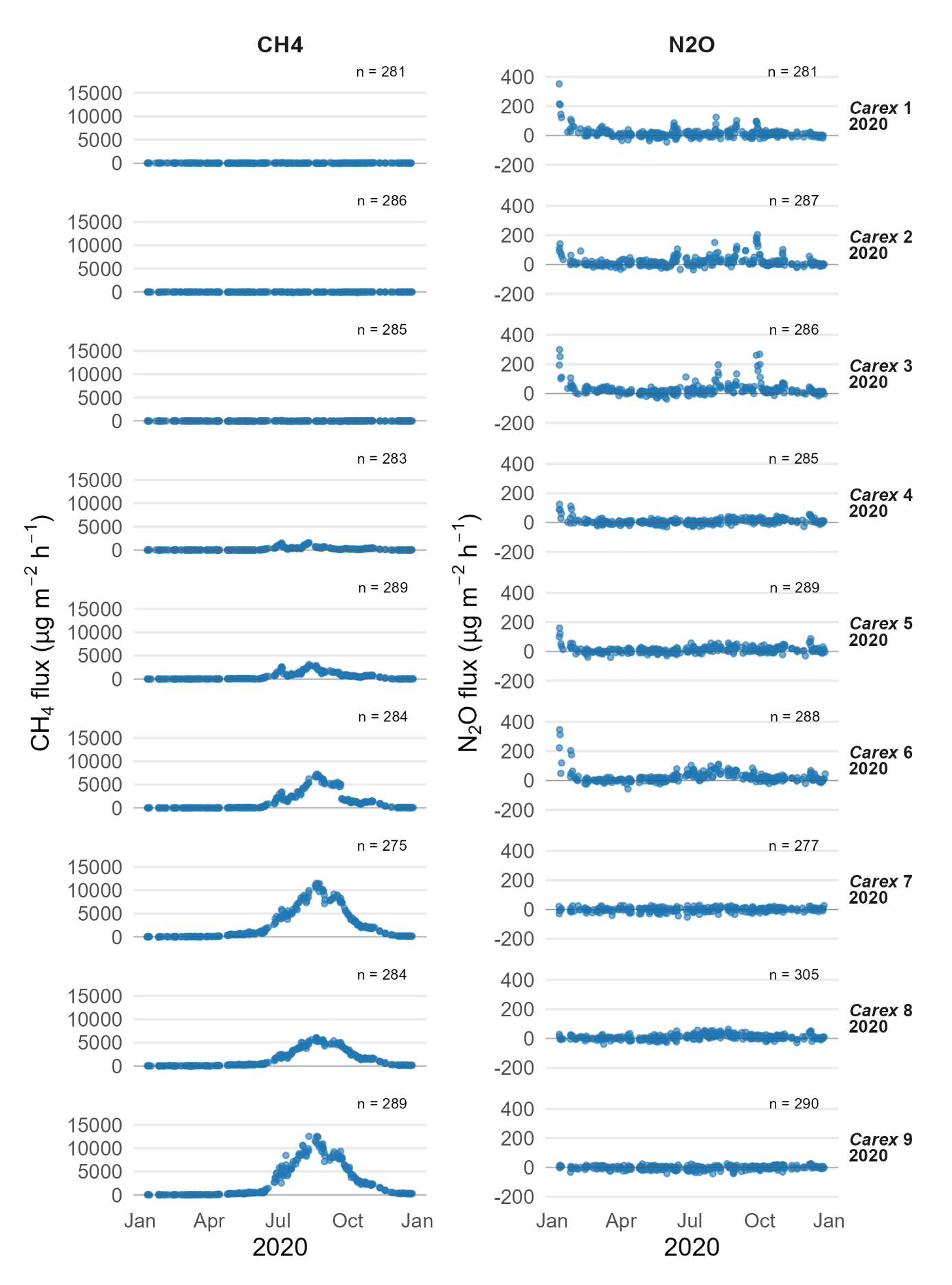
**

**Figure S3b**. Time series of automatically (ARC) measured methane (µg CH_4_ m^−2^ h^−1^) and nitrous oxide (µg N_2_O m^−2^ h^−1^) fluxes are shown for all replicates of the tested paludicultures during the greenhouse gas measurement campaigns in 2020 and 2021 at the site Freisinger Moos (FSM-E). This page: treatment replicates of Carex 2020.

**Figure S3b. continued:** Time series of automatically (ARC) measured methane (µg CH_4_ m^−2^ h^−1^) and nitrous oxide (µg N_2_O m^−2^ h^−1^) fluxes of the *Phalaris* treatment replicates at site FSM-E in 2020.

***
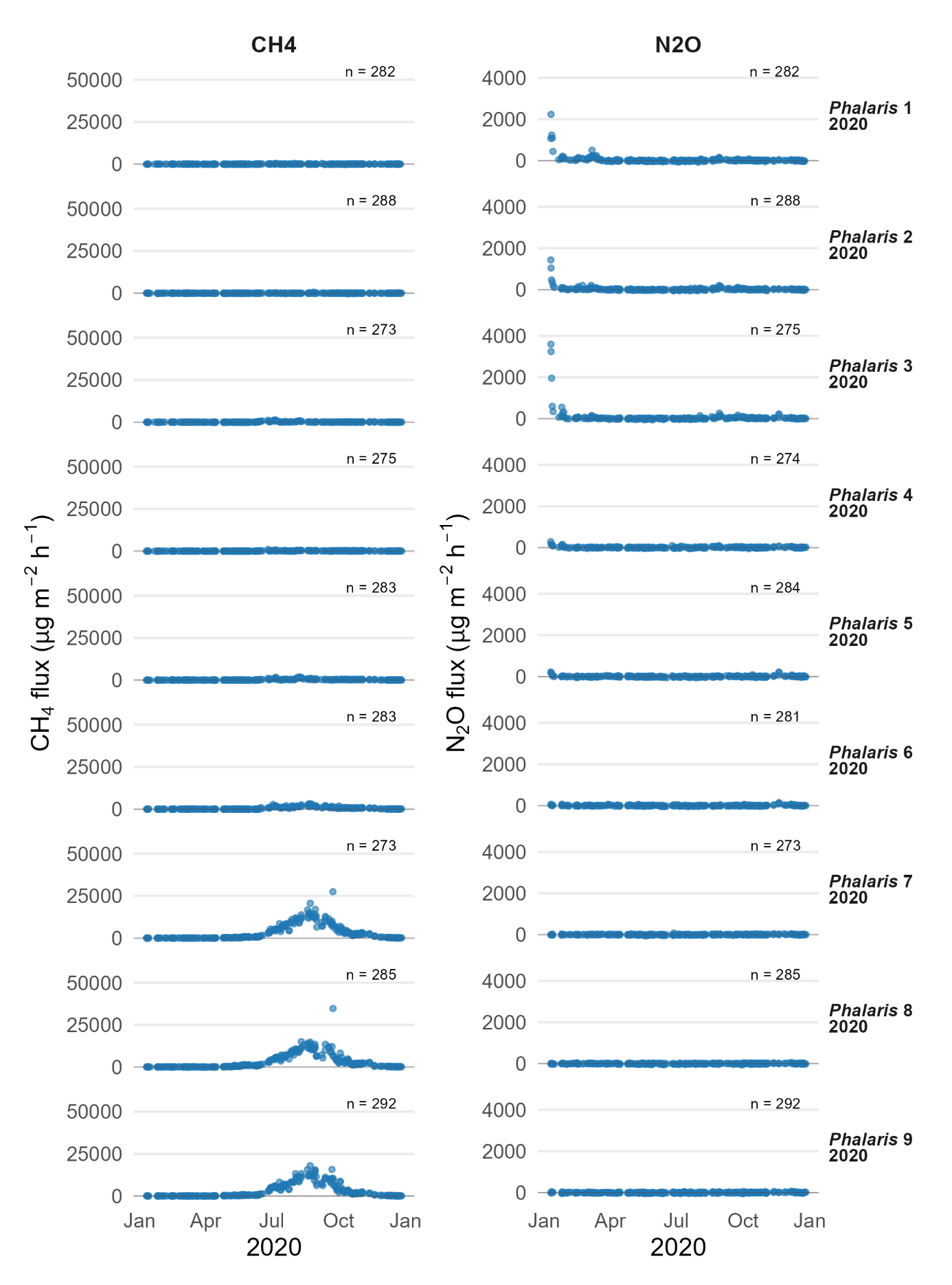
***

**Figure S3b. continued:** Time series of automatically (ARC) measured methane (µg CH_4_ m^−2^ h^−1^) and nitrous oxide (µg N_2_O m^−2^ h^−1^) fluxes of the *Phragmites* treatment replicates at site FSM-E in 2020.

***
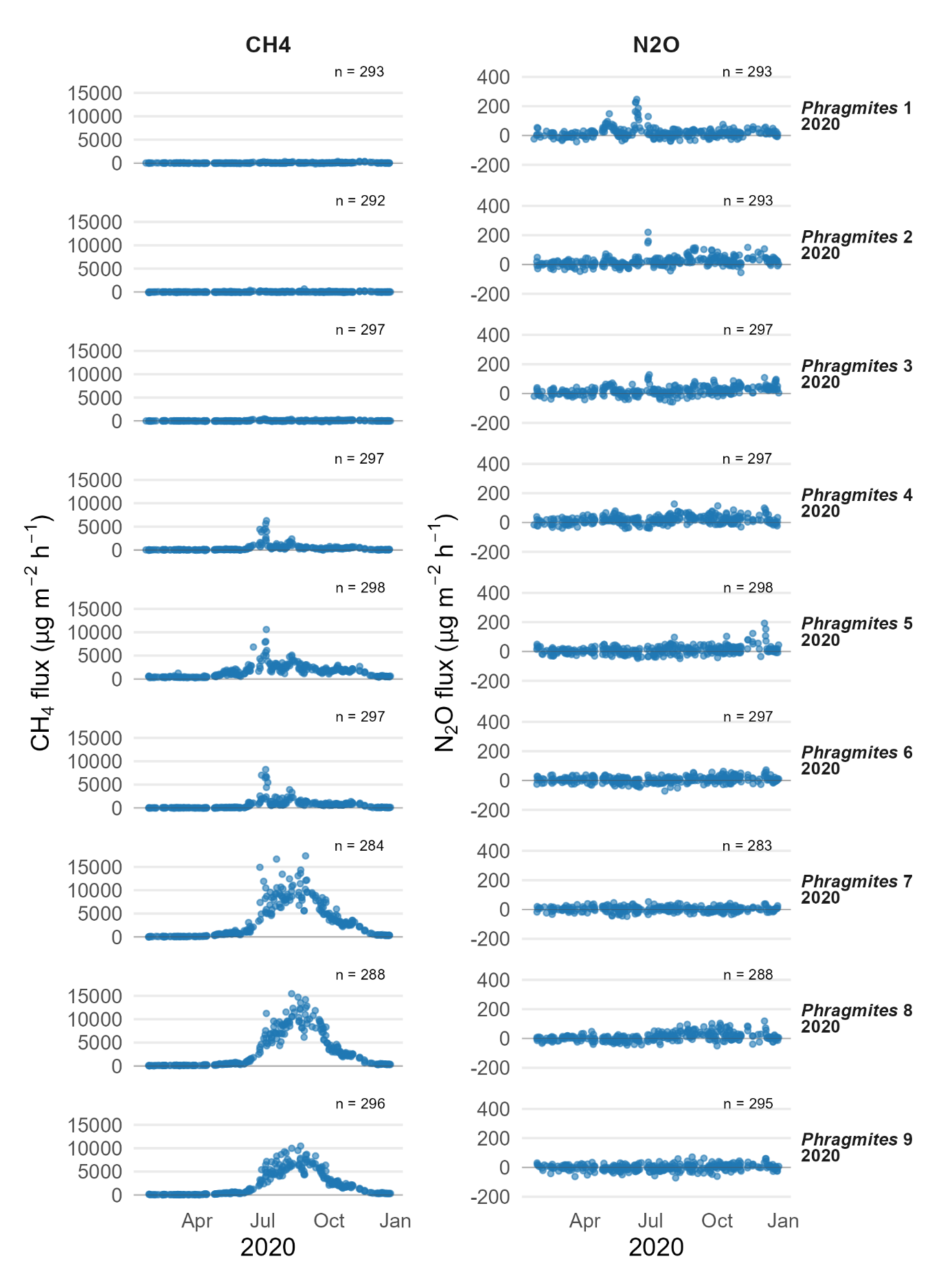
***

**Figure S3b. continued:** Time series of automatically (ARC) measured methane (µg CH_4_ m^−2^ h^−1^) and nitrous oxide (µg N_2_O m^−2^ h^−1^) fluxes of the *Typha* treatment replicates at site FSM-E in 2020.

***
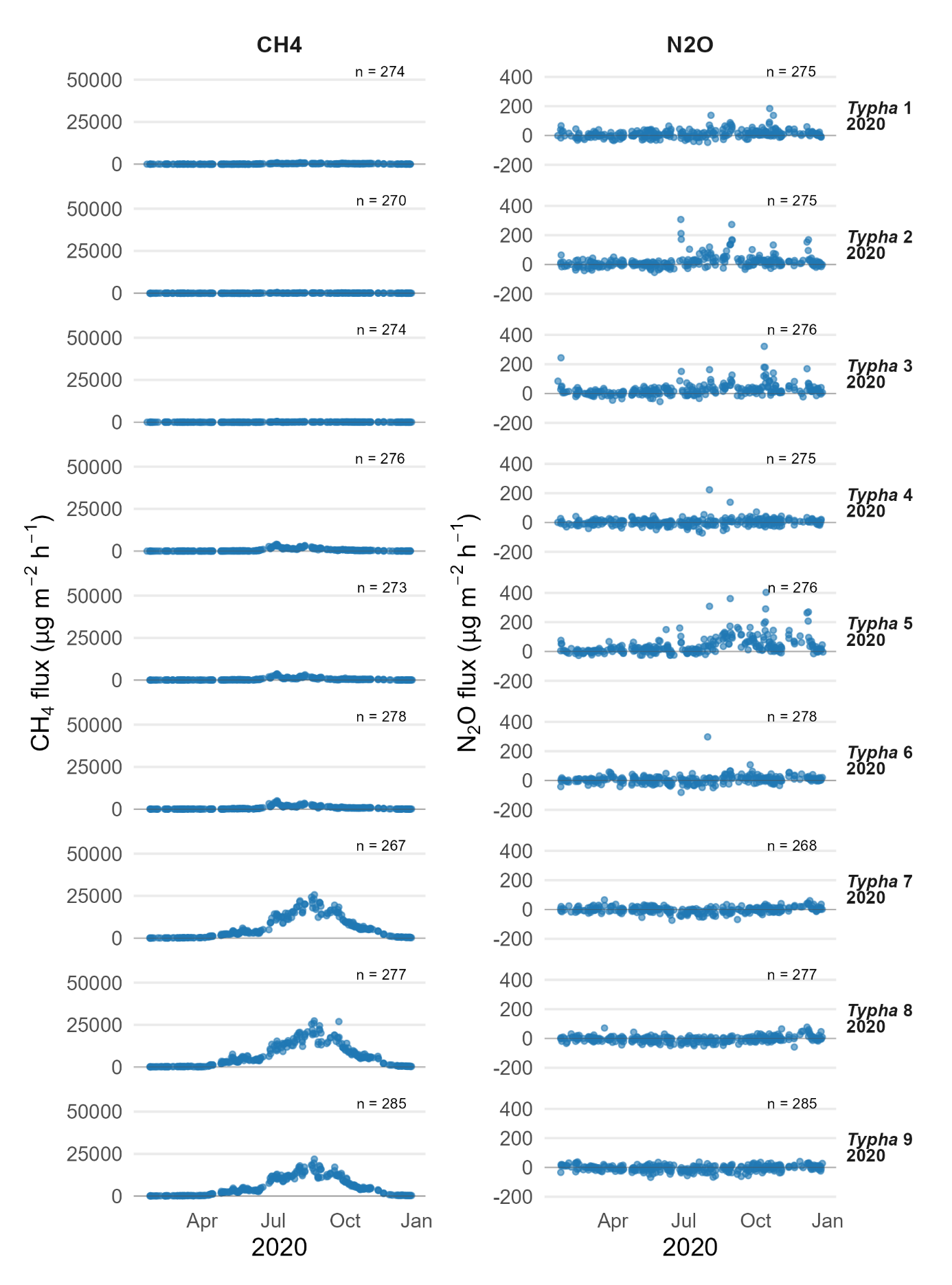
***

**Figure S3b. continued:** Time series of automatically (ARC) measured methane (µg CH_4_ m^−2^ h^−1^) and nitrous oxide (µg N_2_O m^−2^ h^−1^) fluxes of the *Carex* treatment replicates at site FSM-E in 2021.

***
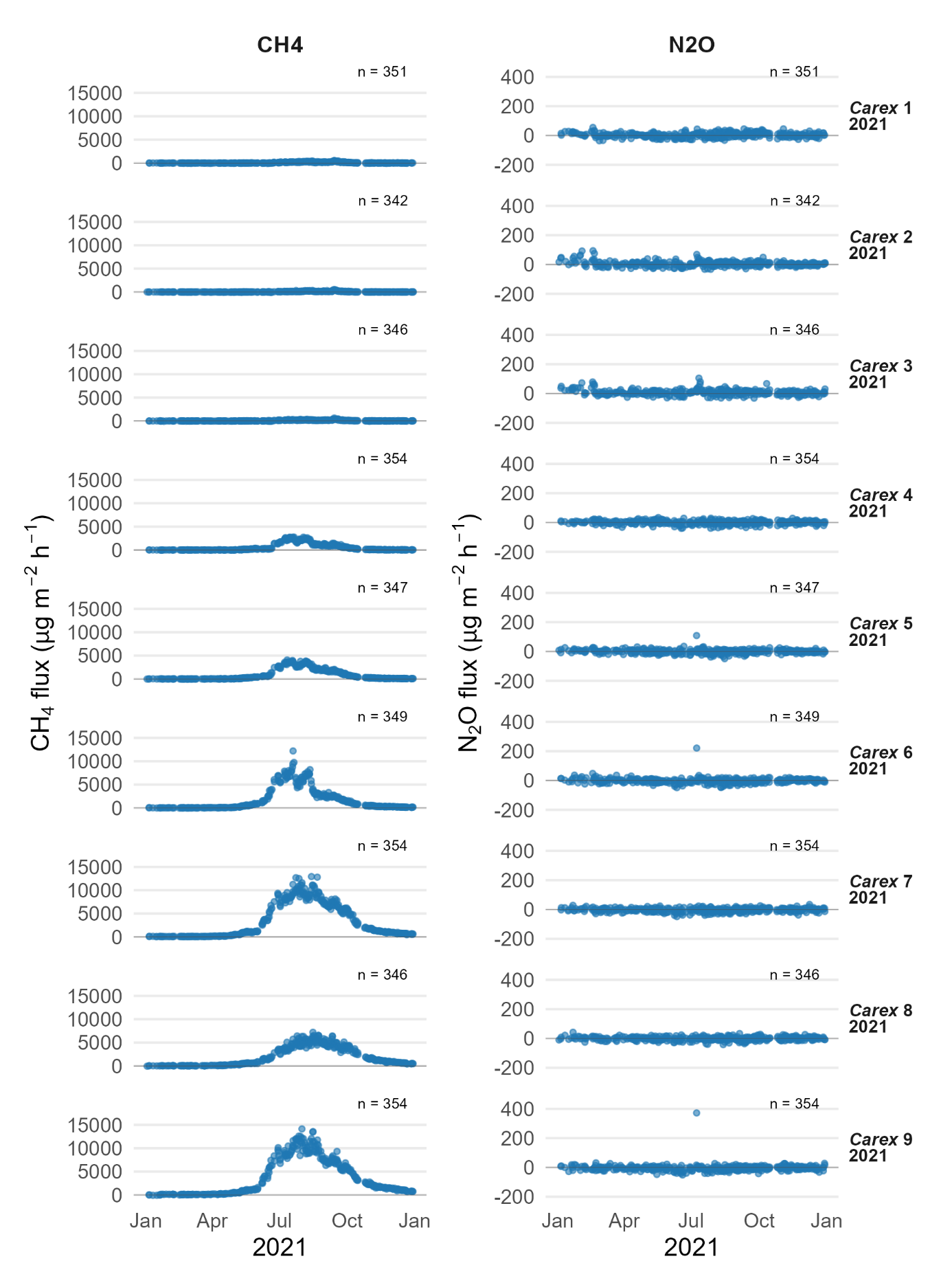
***

**Figure S3b. continued:** Time series of automatically (ARC) measured methane (µg CH_4_ m^−2^ h^−1^) and nitrous oxide (µg N_2_O m^−2^ h^−1^) fluxes of the *Phalaris* treatment replicates at site FSM-E in 2021.

***
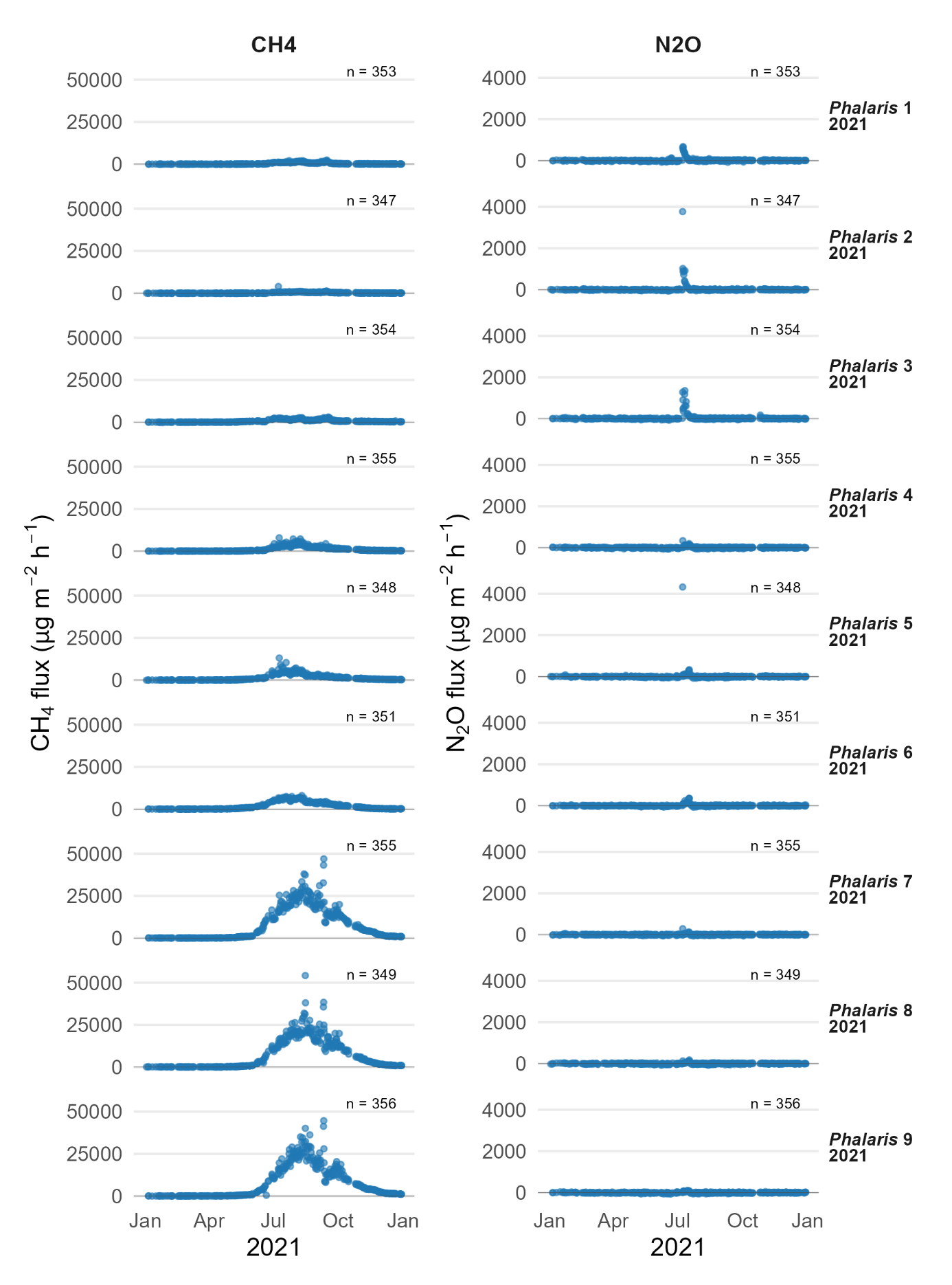
***

**Figure S3b. continued:** Time series of automatically (ARC) measured methane (µg CH_4_ m^−2^ h^−1^) and nitrous oxide (µg N_2_O m^−2^ h^−1^) fluxes of the *Phragmites* treatment replicates at site FSM-E in 2021.

***
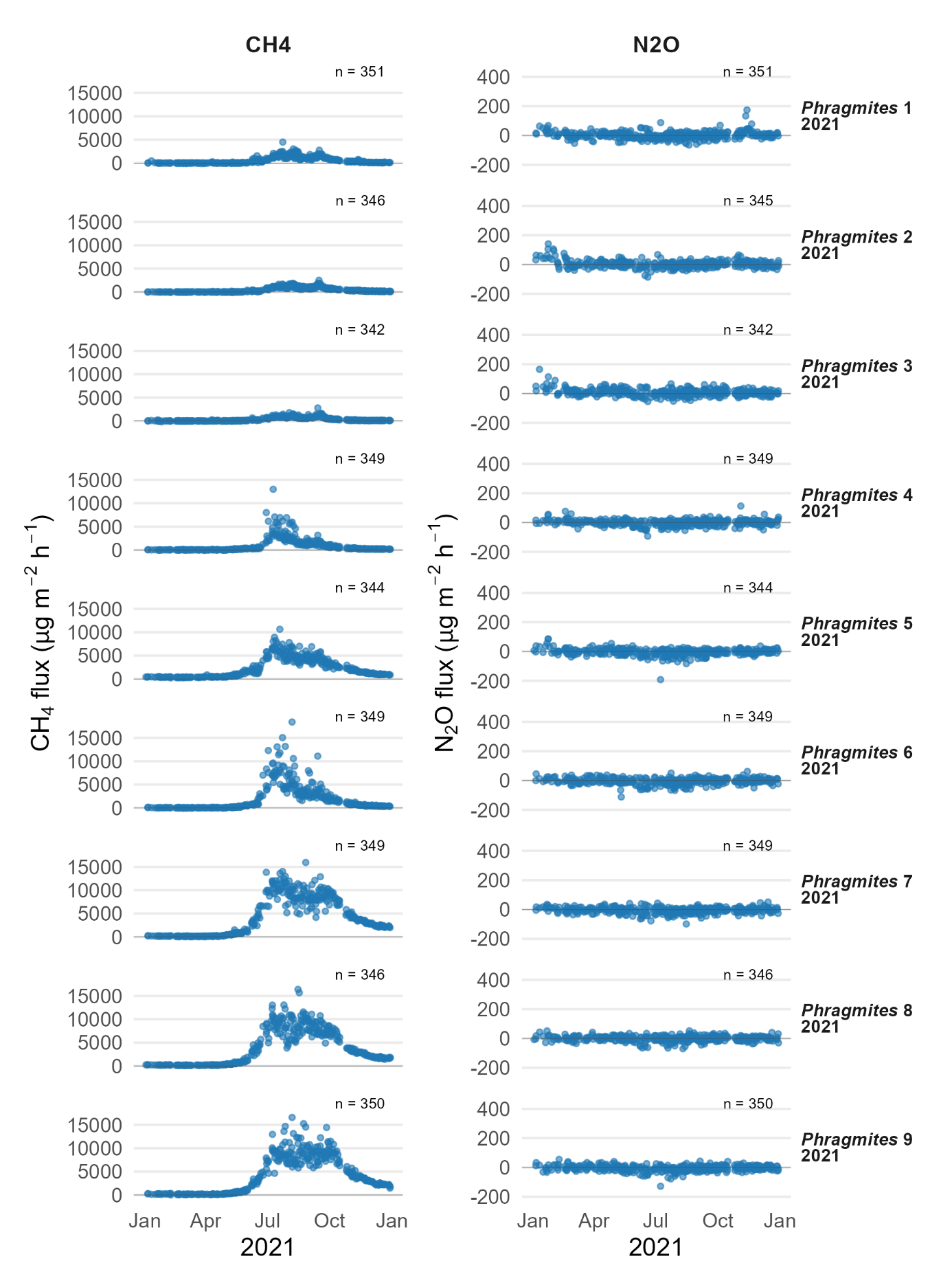
***

**Figure S3b. continued:** Time series of automatically (ARC) measured methane (µg CH_4_ m^−2^ h^−1^) and nitrous oxide (µg N_2_O m^−2^ h^−1^) fluxes of the *Typha* treatment replicates at site FSM-E in 2021.


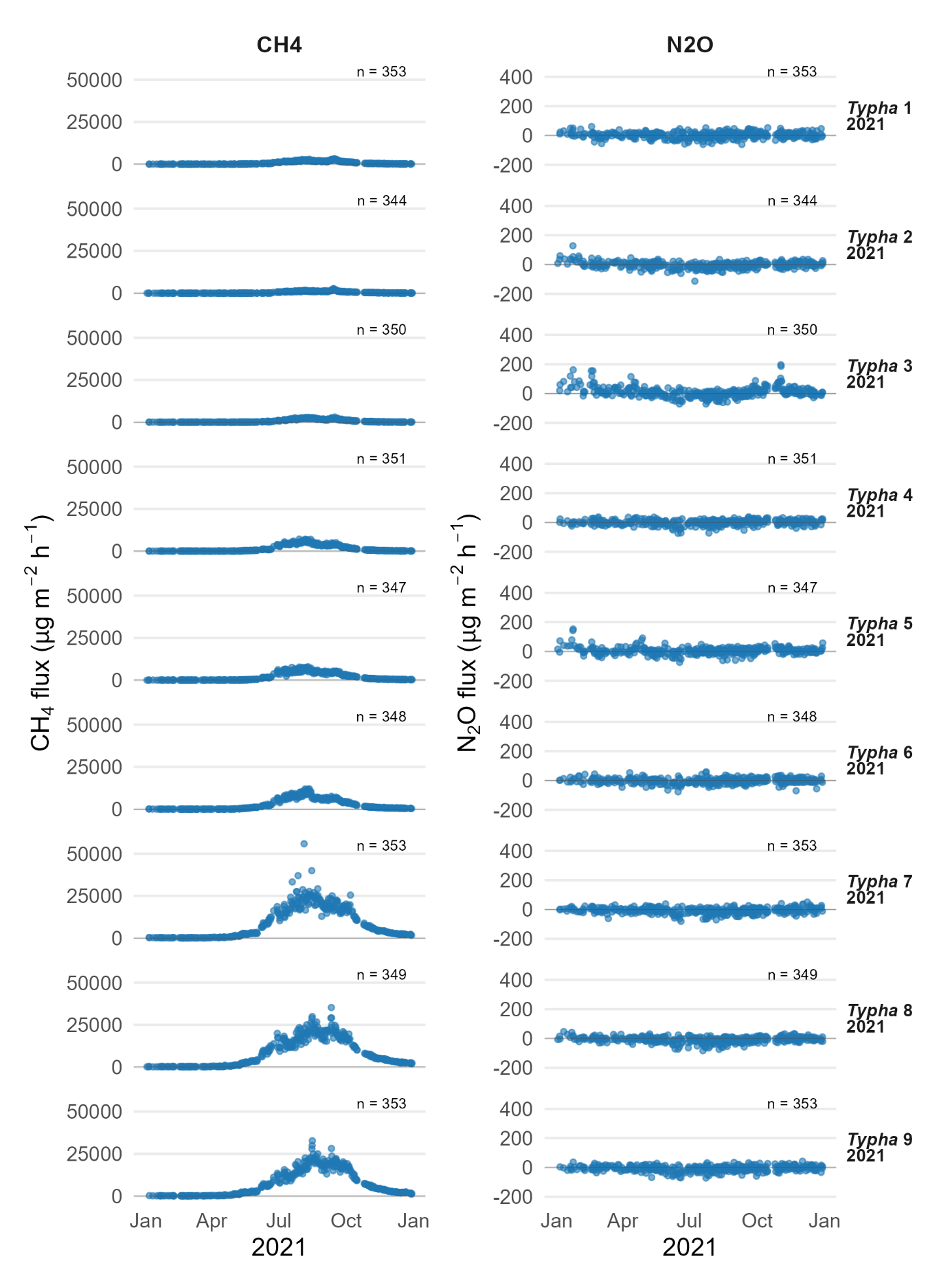


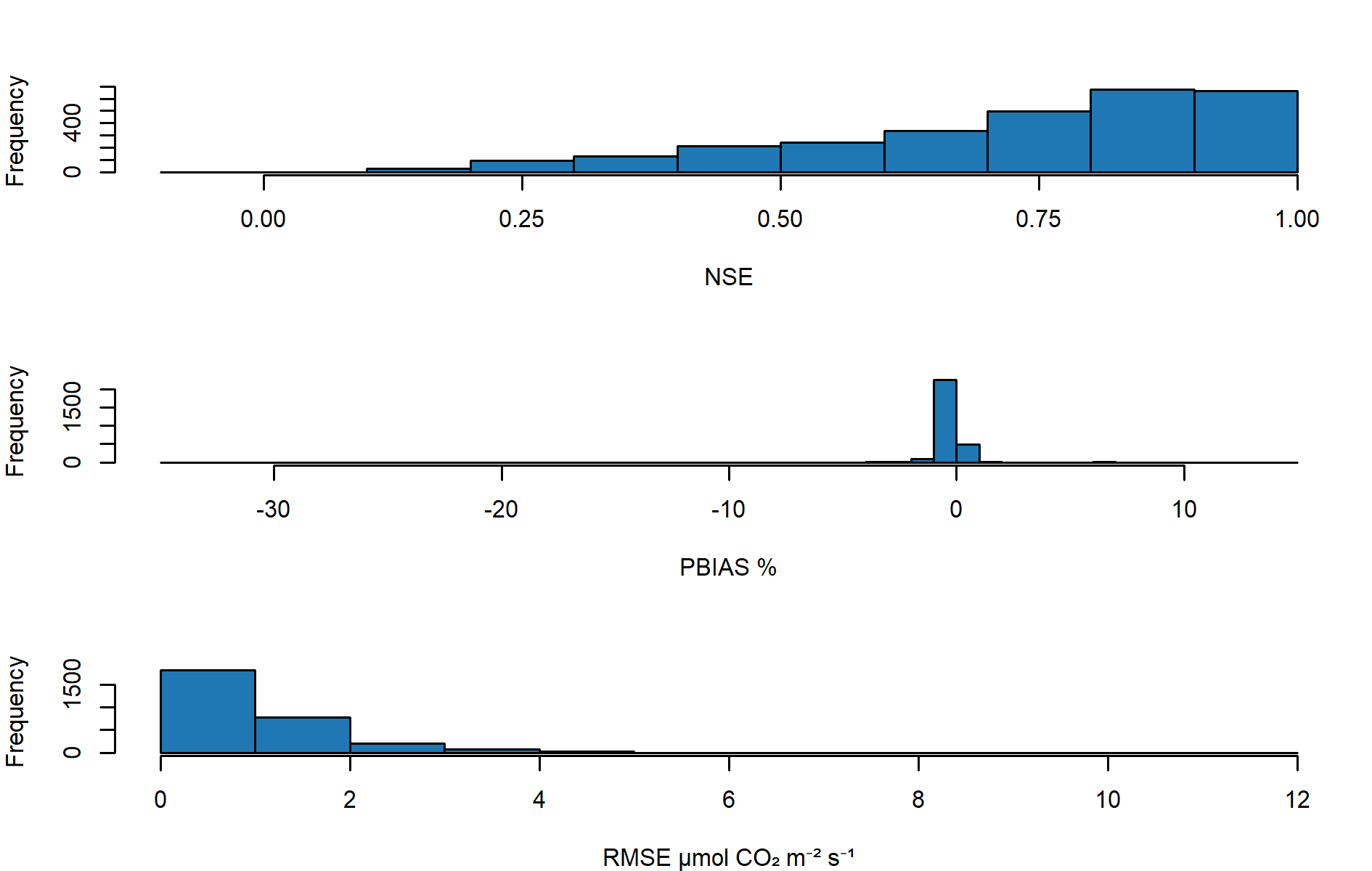


**(a)**


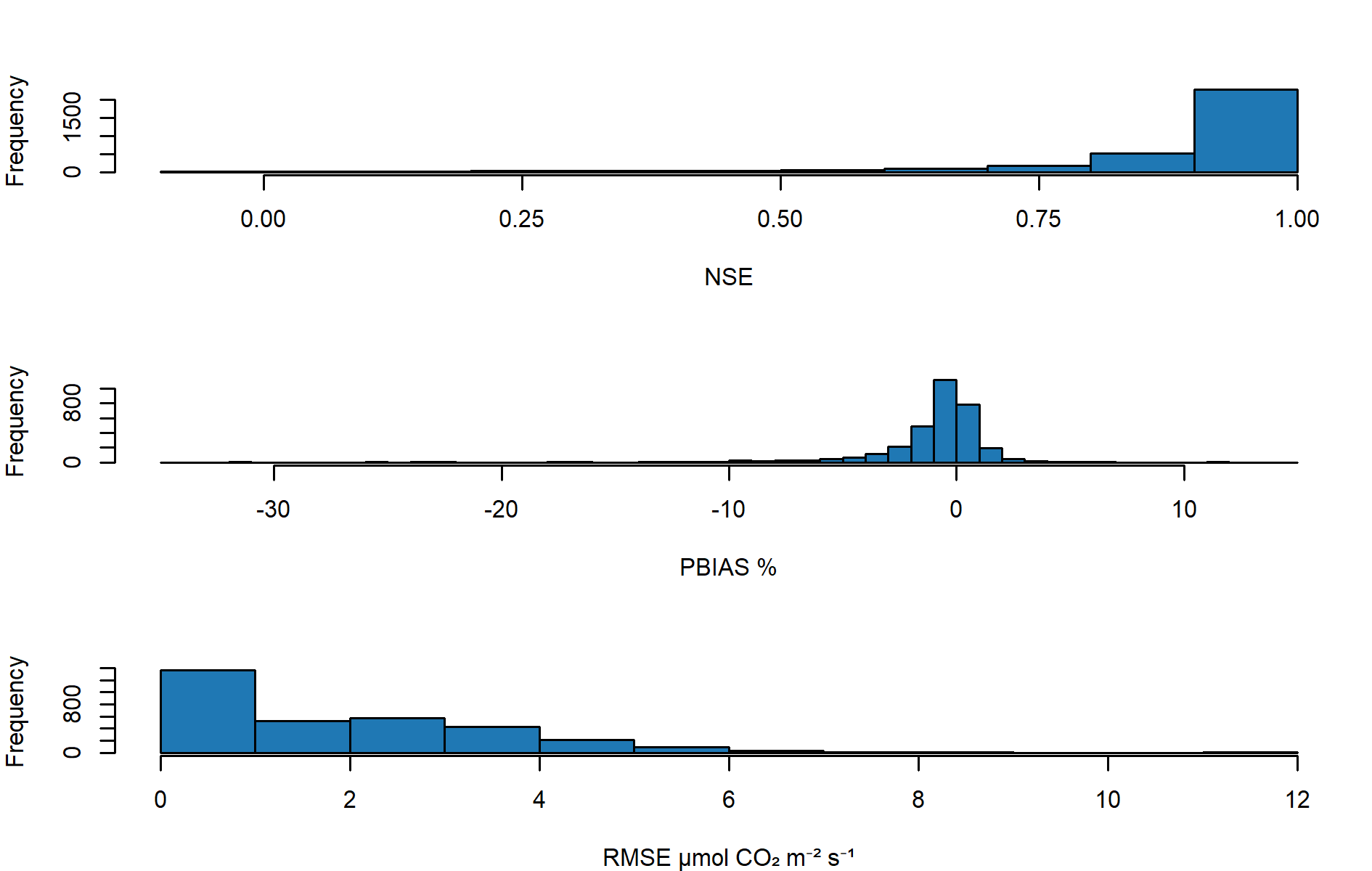


**(b)**

**Figure S4**. Histogramms of model evaluation metrics – Nash-Sutcliffe Efficiency (NSE), Percent Bias (PBIAS), and Root Mean Square Error (RMSE) – for campaign-based models of (a) carbon dioxide ecosystem respiration and (b) gross primary production across all paludiculture treatments. Nash-Sutcliffe Efficiency (NSE) gives the goodness of model fit, with values ≥ 0 indicating better performance than the mean and 1 indicating a perfect match; Percent Bias (PBIAS) represents model accuracy and systematic bias in %, i.e., under- or overestimation; Root Mean Square Error (RMSE) gives the model prediction error in µmol CO_2_ m^−2^ s^−1^ (input unit).


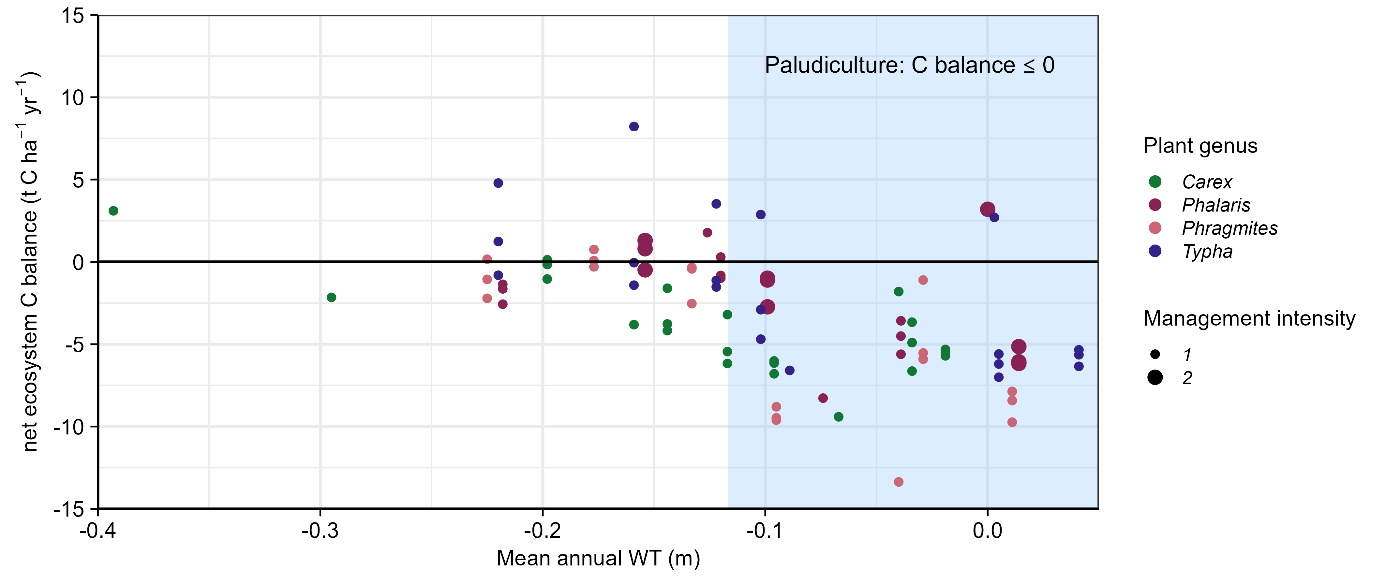


**Figure S5.** Responses of annual net ecosystem carbon balances (NECB in t C ha^−1^ yr^−1^) to the mean annual water table (WT in m) for the paludicultures differentiated by plant genus and management intensity: 1 = one winter harvest; 2 = one summer harvest + one winter harvest + fertilization (in FSM-E treatments only). n = 83 annual treatment (field-scale sites) and replicate (FSM-E ARC) balances. The blue band shows the WT range where the NECB is ≤ 0 indicating peat-preserving potential in line with the definition of paludicultures.

**Table S2**. Model evaluation metrics assessing the accuracy and reliability of annual balance carbon dioxide ecosystem respiration (Reco), gross primary production (GPP) and net ecosystem exchange (NEE) models of all studied paludicultures (n = 81), including Pearson's Correlation Coefficient (Pearson’s *r*), Nash-Sutcliffe Efficiency (NSE), Percent Bias (PBIAS) in %, Root Mean Square Error (RMSE) in μmol CO_2_ m⁻² s⁻¹, Normalized Root Mean Square Error (NRMSE) in %, and Root Mean Square Error to Standard Deviation Ratio (RSR).

| Treatment or  ARC Replicate | Flux | Pearson's *r* | NSE | PBIAS | RMSE | NRMSE | RSR |
| --- | --- | --- | --- | --- | --- | --- | --- |
|  |  |  |  | % | μmol CO_2_  m⁻² s⁻¹ | % |  |
| FSM-F 2019, *Carex* | Reco | 0.99 | 0.99 | −0.20 | 0.65 | 11.00 | 0.11 |
|  | GPP | 0.98 | 0.96 | 0.30 | 3.39 | 18.90 | 0.19 |
|  | NEE | 0.95 | 0.91 | −5.60 | 4.06 | 30.70 | 0.31 |
| FSM-F 2019, *Phalaris* | Reco | 0.95 | 0.90 | −0.30 | 1.79 | 30.90 | 0.31 |
|  | GPP | 0.94 | 0.89 | 0.20 | 5.97 | 33.40 | 0.33 |
|  | NEE | 0.85 | 0.72 | −4.20 | 7.40 | 53.30 | 0.53 |
| FSM-F 2019, *Phragmites* | Reco | 0.99 | 0.98 | −0.20 | 0.87 | 15.50 | 0.16 |
|  | GPP | 0.97 | 0.94 | 0.60 | 5.03 | 23.80 | 0.24 |
|  | NEE | 0.94 | 0.88 | 0.60 | 5.72 | 34.90 | 0.35 |
| FSM-F 2019, *Typha* | Reco | 0.99 | 0.98 | −0.20 | 0.55 | 13.70 | 0.14 |
|  | GPP | 0.99 | 0.97 | 0.40 | 2.56 | 17.00 | 0.17 |
|  | NEE | 0.97 | 0.93 | −5.80 | 3.03 | 25.70 | 0.26 |
| LM 2021, *Carex* | Reco | 0.99 | 0.98 | 0.10 | 0.81 | 12.80 | 0.13 |
|  | GPP | 0.98 | 0.96 | −0.10 | 3.97 | 20.40 | 0.20 |
|  | NEE | 0.94 | 0.89 | −6.80 | 4.88 | 33.50 | 0.34 |
| LM 2021, *Phalaris* | Reco | 0.98 | 0.95 | 0.00 | 1.19 | 21.90 | 0.22 |
|  | GPP | 0.99 | 0.98 | 0.00 | 2.56 | 15.50 | 0.16 |
|  | NEE | 0.97 | 0.94 | −3.60 | 3.01 | 24.00 | 0.24 |
| LM 2021, *Typha* | Reco | 0.99 | 0.97 | 0.00 | 0.65 | 16.50 | 0.17 |
|  | GPP | 0.97 | 0.95 | 0.40 | 2.88 | 22.50 | 0.22 |
|  | NEE | 0.95 | 0.89 | −11.90 | 3.23 | 33.20 | 0.33 |
| RH 2021, *Carex* | Reco | 0.98 | 0.97 | 0.00 | 0.78 | 17.70 | 0.18 |
|  | GPP | 0.98 | 0.97 | 0.30 | 3.22 | 18.00 | 0.18 |
|  | NEE | 0.96 | 0.92 | −4.50 | 4.01 | 27.90 | 0.28 |
| RH 2021, *Phalaris* | Reco | 0.99 | 0.98 | 0.00 | 0.45 | 13.40 | 0.13 |
|  | GPP | 0.98 | 0.96 | 0.00 | 1.69 | 20.70 | 0.21 |
|  | NEE | 0.93 | 0.86 | −5.70 | 2.12 | 37.80 | 0.38 |
| FSM-E 2020, *Carex* 1 | Reco | 0.97 | 0.94 | −0.10 | 1.09 | 24.50 | 0.24 |
|  | GPP | 0.98 | 0.97 | −0.30 | 2.27 | 18.30 | 0.18 |
|  | NEE | 0.93 | 0.86 | 17.70 | 3.79 | 38.00 | 0.38 |
| FSM-E 2020, *Carex* 2 | Reco | 0.97 | 0.95 | −0.10 | 0.96 | 23.30 | 0.23 |
|  | GPP | 0.99 | 0.97 | 0.00 | 1.97 | 17.20 | 0.17 |
|  | NEE | 0.94 | 0.87 | 19.70 | 3.12 | 35.50 | 0.35 |
| FSM-E 2020, *Carex* 3 | Reco | 0.97 | 0.94 | −0.10 | 1.10 | 23.40 | 0.23 |
|  | GPP | 0.98 | 0.97 | 0.00 | 2.30 | 18.20 | 0.18 |
|  | NEE | 0.92 | 0.83 | 23.30 | 4.00 | 41.60 | 0.42 |
| FSM-E 2020, *Carex* 4 | Reco | 0.96 | 0.92 | −0.10 | 1.31 | 27.90 | 0.28 |
|  | GPP | 0.98 | 0.97 | −0.20 | 2.53 | 18.00 | 0.18 |
|  | NEE | 0.93 | 0.85 | 18.10 | 4.28 | 38.40 | 0.38 |
| FSM-E 2020, *Carex* 5 | Reco | 0.97 | 0.95 | −0.10 | 1.09 | 23.00 | 0.23 |
|  | GPP | 0.98 | 0.97 | 0.20 | 2.77 | 17.70 | 0.18 |
|  | NEE | 0.93 | 0.85 | 17.50 | 4.68 | 38.30 | 0.38 |
| FSM-E 2020, *Carex* 6 | Reco | 0.97 | 0.94 | −0.10 | 1.14 | 23.50 | 0.23 |
|  | GPP | 0.98 | 0.97 | −0.10 | 3.02 | 18.20 | 0.18 |
|  | NEE | 0.94 | 0.85 | 19.10 | 5.00 | 38.40 | 0.38 |
| FSM-E 2020, *Carex* 7 | Reco | 0.98 | 0.97 | −0.20 | 0.86 | 17.50 | 0.18 |
|  | GPP | 0.98 | 0.97 | −0.30 | 2.81 | 17.40 | 0.17 |
|  | NEE | 0.93 | 0.84 | 17.00 | 5.03 | 40.30 | 0.40 |
| FSM-E 2020, *Carex* 8 | Reco | 0.97 | 0.93 | −0.20 | 1.25 | 25.70 | 0.26 |
|  | GPP | 0.98 | 0.96 | −0.50 | 3.22 | 20.40 | 0.20 |
|  | NEE | 0.92 | 0.82 | 15.80 | 5.28 | 42.50 | 0.43 |
| FSM-E 2020, *Carex* 9 | Reco | 0.98 | 0.96 | −0.20 | 0.96 | 19.00 | 0.19 |
|  | GPP | 0.98 | 0.96 | −0.30 | 3.07 | 18.90 | 0.19 |
|  | NEE | 0.93 | 0.84 | 17.30 | 4.92 | 39.60 | 0.40 |
| FSM-E 2020, *Phalaris* 1 | Reco | 0.97 | 0.95 | 0.00 | 1.50 | 22.90 | 0.23 |
|  | GPP | 0.98 | 0.97 | −0.90 | 2.68 | 18.40 | 0.18 |
|  | NEE | 0.93 | 0.86 | 17.90 | 4.29 | 37.70 | 0.38 |
| FSM-E 2020, *Phalaris* 2 | Reco | 0.97 | 0.95 | −0.10 | 1.52 | 22.30 | 0.22 |
|  | GPP | 0.98 | 0.97 | −0.60 | 2.62 | 18.30 | 0.18 |
|  | NEE | 0.94 | 0.86 | 23.40 | 4.06 | 37.40 | 0.37 |
| FSM-E 2020, *Phalaris* 3 | Reco | 0.98 | 0.96 | 0.00 | 1.33 | 20.30 | 0.20 |
|  | GPP | 0.98 | 0.96 | −0.30 | 2.83 | 20.10 | 0.20 |
|  | NEE | 0.92 | 0.83 | 25.50 | 4.47 | 41.10 | 0.41 |
| FSM-E 2020, *Phalaris* 4 | Reco | 0.96 | 0.92 | 0.00 | 1.95 | 27.40 | 0.27 |
|  | GPP | 0.98 | 0.97 | −0.90 | 2.80 | 18.20 | 0.18 |
|  | NEE | 0.92 | 0.83 | 21.30 | 4.98 | 41.80 | 0.42 |
| FSM-E 2020, *Phalaris* 5 | Reco | 0.97 | 0.94 | 0.00 | 1.67 | 23.90 | 0.24 |
|  | GPP | 0.97 | 0.94 | −0.70 | 4.05 | 25.20 | 0.25 |
|  | NEE | 0.90 | 0.80 | 20.00 | 5.44 | 44.50 | 0.45 |
| FSM-E 2020, *Phalaris* 6 | Reco | 0.92 | 0.85 | 0.00 | 2.45 | 39.10 | 0.39 |
|  | GPP | 0.98 | 0.96 | −0.70 | 2.88 | 20.70 | 0.21 |
|  | NEE | 0.91 | 0.80 | 22.10 | 4.74 | 44.60 | 0.45 |
| FSM-E 2020, *Phalaris* 7 | Reco | 0.95 | 0.90 | −0.20 | 1.46 | 31.50 | 0.32 |
|  | GPP | 0.98 | 0.97 | −0.70 | 2.31 | 17.70 | 0.18 |
|  | NEE | 0.93 | 0.82 | 22.50 | 4.21 | 42.00 | 0.42 |
| FSM-E 2020, *Phalaris* 8 | Reco | 0.95 | 0.90 | −0.10 | 1.44 | 31.40 | 0.31 |
|  | GPP | 0.98 | 0.96 | −0.70 | 2.37 | 19.10 | 0.19 |
|  | NEE | 0.92 | 0.83 | 19.30 | 3.92 | 41.00 | 0.41 |
| FSM-E 2020, *Phalaris* 9 | Reco | 0.96 | 0.92 | −0.10 | 1.40 | 27.80 | 0.28 |
|  | GPP | 0.98 | 0.96 | −0.40 | 2.75 | 19.30 | 0.19 |
|  | NEE | 0.93 | 0.85 | 16.40 | 4.29 | 39.30 | 0.39 |
| FSM-E 2020, *Phragmites* 1 | Reco | 0.97 | 0.94 | −0.10 | 0.98 | 25.00 | 0.25 |
|  | GPP | 0.99 | 0.98 | −0.30 | 1.84 | 14.30 | 0.14 |
|  | NEE | 0.93 | 0.85 | 9.40 | 3.75 | 38.20 | 0.38 |
| FSM-E 2020, *Phragmites* 2 | Reco | 0.97 | 0.95 | −0.10 | 0.91 | 22.90 | 0.23 |
|  | GPP | 0.98 | 0.96 | −0.40 | 2.34 | 19.60 | 0.20 |
|  | NEE | 0.89 | 0.78 | 9.00 | 4.19 | 46.80 | 0.47 |
| FSM-E 2020, *Phragmites* 3 | Reco | 0.98 | 0.97 | −0.10 | 0.84 | 17.30 | 0.17 |
|  | GPP | 0.98 | 0.97 | −0.20 | 2.54 | 17.30 | 0.17 |
|  | NEE | 0.94 | 0.86 | 14.20 | 3.97 | 36.80 | 0.37 |
| FSM-E 2020, *Phragmites* 4 | Reco | 0.98 | 0.95 | −0.20 | 1.22 | 22.10 | 0.22 |
|  | GPP | 0.99 | 0.97 | −0.10 | 2.90 | 17.10 | 0.17 |
|  | NEE | 0.91 | 0.82 | 13.80 | 5.32 | 42.40 | 0.42 |
| FSM-E 2020, *Phragmites* 5 | Reco | 0.97 | 0.94 | −0.10 | 1.53 | 25.40 | 0.25 |
|  | GPP | 0.98 | 0.97 | −0.20 | 3.08 | 17.90 | 0.18 |
|  | NEE | 0.92 | 0.85 | 9.80 | 4.97 | 39.10 | 0.39 |
| FSM-E 2020, *Phragmites* 6 | Reco | 0.97 | 0.95 | −0.10 | 1.39 | 22.40 | 0.22 |
|  | GPP | 0.98 | 0.97 | 0.30 | 3.07 | 18.20 | 0.18 |
|  | NEE | 0.94 | 0.87 | 12.20 | 4.43 | 36.30 | 0.36 |
| FSM-E 2020, *Phragmites* 7 | Reco | 0.96 | 0.93 | −0.20 | 1.45 | 27.00 | 0.27 |
|  | GPP | 0.99 | 0.98 | −0.30 | 2.41 | 15.60 | 0.16 |
|  | NEE | 0.94 | 0.88 | 10.60 | 4.26 | 35.10 | 0.35 |
| FSM-E 2020, *Phragmites* 8 | Reco | 0.89 | 0.79 | −0.20 | 2.63 | 46.00 | 0.46 |
|  | GPP | 0.98 | 0.97 | −0.80 | 2.79 | 18.70 | 0.19 |
|  | NEE | 0.92 | 0.83 | 11.70 | 4.99 | 41.50 | 0.42 |
| FSM-E 2020, *Phragmites* 9 | Reco | 0.97 | 0.95 | −0.10 | 1.04 | 22.60 | 0.23 |
|  | GPP | 0.99 | 0.97 | −0.40 | 2.52 | 17.10 | 0.17 |
|  | NEE | 0.95 | 0.90 | 11.00 | 3.71 | 32.30 | 0.32 |
| FSM-E 2020, *Typha* 1 | Reco | 0.98 | 0.96 | −0.20 | 0.84 | 21.20 | 0.21 |
|  | GPP | 0.99 | 0.97 | −0.20 | 2.33 | 16.90 | 0.17 |
|  | NEE | 0.94 | 0.86 | 20.50 | 4.03 | 36.90 | 0.37 |
| FSM-E 2020, *Typha* 2 | Reco | 0.96 | 0.91 | −0.10 | 1.13 | 29.30 | 0.29 |
|  | GPP | 0.98 | 0.95 | −0.10 | 2.68 | 22.10 | 0.22 |
|  | NEE | 0.91 | 0.80 | 32.10 | 4.54 | 45.00 | 0.45 |
| FSM-E 2020, *Typha* 3 | Reco | 0.95 | 0.90 | −0.10 | 1.36 | 31.80 | 0.32 |
|  | GPP | 0.86 | 0.73 | 0.10 | 6.77 | 51.50 | 0.51 |
|  | NEE | 0.76 | 0.56 | 14.10 | 7.07 | 65.90 | 0.66 |
| FSM-E 2020, *Typha* 4 | Reco | 0.95 | 0.90 | −0.30 | 1.53 | 31.60 | 0.32 |
|  | GPP | 0.98 | 0.97 | 0.40 | 2.45 | 18.40 | 0.18 |
|  | NEE | 0.92 | 0.80 | 39.70 | 4.81 | 45.10 | 0.45 |
| FSM-E 2020, *Typha* 5 | Reco | 0.93 | 0.86 | −0.20 | 2.40 | 36.80 | 0.37 |
|  | GPP | 0.97 | 0.95 | 0.30 | 3.74 | 23.30 | 0.23 |
|  | NEE | 0.90 | 0.77 | 62.10 | 6.48 | 47.90 | 0.48 |
| FSM-E 2020, *Typha* 6 | Reco | 0.95 | 0.91 | −0.10 | 1.53 | 30.10 | 0.30 |
|  | GPP | 0.98 | 0.95 | 0.20 | 3.10 | 22.10 | 0.22 |
|  | NEE | 0.90 | 0.75 | 34.10 | 5.43 | 49.60 | 0.50 |
| FSM-E 2020, *Typha* 7 | Reco | 0.98 | 0.96 | −0.10 | 0.72 | 19.80 | 0.20 |
|  | GPP | 0.99 | 0.97 | −0.20 | 2.32 | 16.40 | 0.16 |
|  | NEE | 0.92 | 0.82 | 22.10 | 4.84 | 42.50 | 0.43 |
| FSM-E 2020, *Typha* 8 | Reco | 0.95 | 0.91 | −0.10 | 0.90 | 29.80 | 0.30 |
|  | GPP | 0.98 | 0.97 | −0.30 | 2.13 | 18.70 | 0.19 |
|  | NEE | 0.93 | 0.82 | 22.40 | 3.85 | 41.90 | 0.42 |
| FSM-E 2020, *Typha* 9 | Reco | 0.98 | 0.96 | −0.20 | 0.74 | 20.60 | 0.21 |
|  | GPP | 0.98 | 0.96 | −0.10 | 2.59 | 19.10 | 0.19 |
|  | NEE | 0.95 | 0.87 | 19.90 | 3.90 | 36.40 | 0.36 |
| FSM-E 2021, *Carex* 1 | Reco | 0.98 | 0.96 | −0.10 | 0.90 | 19.60 | 0.20 |
|  | GPP | 0.99 | 0.98 | −0.30 | 1.73 | 12.60 | 0.13 |
|  | NEE | 0.95 | 0.90 | 13.70 | 3.46 | 32.20 | 0.32 |
| FSM-E 2021, *Carex* 2 | Reco | 0.98 | 0.96 | −0.10 | 0.83 | 20.60 | 0.21 |
|  | GPP | 0.99 | 0.98 | −0.20 | 1.39 | 12.30 | 0.12 |
|  | NEE | 0.95 | 0.88 | 17.20 | 3.02 | 33.90 | 0.34 |
| FSM-E 2021, *Carex* 3 | Reco | 0.98 | 0.96 | −0.10 | 0.95 | 20.00 | 0.20 |
|  | GPP | 0.99 | 0.98 | −0.50 | 1.86 | 13.60 | 0.14 |
|  | NEE | 0.94 | 0.87 | 15.10 | 3.80 | 36.20 | 0.36 |
| FSM-E 2021, *Carex* 4 | Reco | 0.98 | 0.96 | −0.10 | 1.13 | 20.70 | 0.21 |
|  | GPP | 0.99 | 0.98 | −0.30 | 2.46 | 15.70 | 0.16 |
|  | NEE | 0.94 | 0.88 | 15.50 | 4.26 | 34.50 | 0.34 |
| FSM-E 2021, *Carex* 5 | Reco | 0.98 | 0.96 | −0.20 | 1.06 | 20.00 | 0.20 |
|  | GPP | 0.99 | 0.98 | −0.10 | 2.42 | 14.60 | 0.15 |
|  | NEE | 0.94 | 0.88 | 16.90 | 4.59 | 35.00 | 0.35 |
| FSM-E 2021, *Carex* 6 | Reco | 0.97 | 0.95 | −0.10 | 1.27 | 22.60 | 0.23 |
|  | GPP | 0.99 | 0.98 | −0.50 | 2.72 | 15.50 | 0.15 |
|  | NEE | 0.94 | 0.86 | 16.70 | 5.10 | 36.90 | 0.37 |
| FSM-E 2021, *Carex* 7 | Reco | 0.99 | 0.98 | −0.10 | 0.76 | 13.80 | 0.14 |
|  | GPP | 0.99 | 0.98 | −0.50 | 2.42 | 13.80 | 0.14 |
|  | NEE | 0.95 | 0.90 | 13.20 | 4.37 | 32.20 | 0.32 |
| FSM-E 2021, *Carex* 8 | Reco | 0.99 | 0.97 | −0.10 | 0.83 | 16.60 | 0.17 |
|  | GPP | 0.99 | 0.98 | −0.30 | 2.36 | 14.50 | 0.14 |
|  | NEE | 0.95 | 0.89 | 13.90 | 4.27 | 33.20 | 0.33 |
| FSM-E 2021, *Carex* 9 | Reco | 0.99 | 0.98 | −0.10 | 0.76 | 13.80 | 0.14 |
|  | GPP | 0.99 | 0.98 | −0.60 | 2.51 | 14.80 | 0.15 |
|  | NEE | 0.94 | 0.88 | 14.30 | 4.56 | 35.00 | 0.35 |
| FSM-E 2021, *Phalaris* 1 | Reco | 0.97 | 0.94 | 0.00 | 1.46 | 23.60 | 0.24 |
|  | GPP | 0.98 | 0.97 | −1.00 | 2.51 | 17.60 | 0.18 |
|  | NEE | 0.93 | 0.85 | 23.10 | 4.35 | 38.20 | 0.38 |
| FSM-E 2021, *Phalaris* 2 | Reco | 0.98 | 0.96 | 0.00 | 1.14 | 20.00 | 0.20 |
|  | GPP | 0.98 | 0.97 | −0.50 | 2.57 | 18.60 | 0.19 |
|  | NEE | 0.92 | 0.83 | 26.50 | 4.52 | 40.70 | 0.41 |
| FSM-E 2021, *Phalaris* 3 | Reco | 0.97 | 0.94 | 0.00 | 1.58 | 24.60 | 0.25 |
|  | GPP | 0.98 | 0.97 | −0.40 | 2.81 | 18.30 | 0.18 |
|  | NEE | 0.91 | 0.81 | 20.90 | 5.21 | 44.10 | 0.44 |
| FSM-E 2021, *Phalaris* 4 | Reco | 0.97 | 0.94 | 0.00 | 1.43 | 24.60 | 0.25 |
|  | GPP | 0.99 | 0.97 | −0.50 | 2.50 | 16.20 | 0.16 |
|  | NEE | 0.93 | 0.86 | 17.30 | 4.64 | 37.70 | 0.38 |
| FSM-E 2021, *Phalaris* 5 | Reco | 0.97 | 0.94 | 0.00 | 1.54 | 24.20 | 0.24 |
|  | GPP | 0.99 | 0.97 | −0.60 | 2.88 | 16.50 | 0.17 |
|  | NEE | 0.93 | 0.85 | 20.20 | 5.25 | 38.20 | 0.38 |
| FSM-E 2021, *Phalaris* 6 | Reco | 0.96 | 0.93 | 0.00 | 1.65 | 26.70 | 0.27 |
|  | GPP | 0.99 | 0.97 | −0.40 | 2.64 | 17.20 | 0.17 |
|  | NEE | 0.92 | 0.83 | 20.70 | 4.94 | 41.20 | 0.41 |
| FSM-E 2021, *Phalaris* 7 | Reco | 0.98 | 0.96 | −0.10 | 1.05 | 20.90 | 0.21 |
|  | GPP | 0.99 | 0.98 | −0.40 | 2.52 | 15.10 | 0.15 |
|  | NEE | 0.94 | 0.87 | 13.90 | 4.85 | 35.60 | 0.36 |
| FSM-E 2021, *Phalaris* 8 | Reco | 0.98 | 0.97 | 0.00 | 0.86 | 18.20 | 0.18 |
|  | GPP | 0.99 | 0.98 | −0.40 | 2.30 | 14.40 | 0.14 |
|  | NEE | 0.94 | 0.87 | 15.40 | 4.65 | 36.10 | 0.36 |
| FSM-E 2021, *Phalaris* 9 | Reco | 0.98 | 0.96 | 0.00 | 1.10 | 20.60 | 0.21 |
|  | GPP | 0.99 | 0.97 | −0.50 | 3.06 | 16.50 | 0.17 |
|  | NEE | 0.94 | 0.87 | 14.60 | 5.50 | 36.60 | 0.37 |
| FSM-E 2021, *Phragmites* 1 | Reco | 0.96 | 0.91 | −0.10 | 0.92 | 29.40 | 0.29 |
|  | GPP | 0.99 | 0.98 | −0.90 | 1.38 | 14.30 | 0.14 |
|  | NEE | 0.94 | 0.88 | 15.30 | 2.71 | 35.20 | 0.35 |
| FSM-E 2021, *Phragmites* 2 | Reco | 0.97 | 0.94 | −0.10 | 0.89 | 23.70 | 0.24 |
|  | GPP | 0.99 | 0.98 | −0.30 | 1.42 | 12.90 | 0.13 |
|  | NEE | 0.94 | 0.86 | 17.20 | 3.05 | 37.10 | 0.37 |
| FSM-E 2021, *Phragmites* 3 | Reco | 0.94 | 0.88 | 0.00 | 1.96 | 34.70 | 0.35 |
|  | GPP | 0.98 | 0.97 | −0.30 | 2.59 | 18.00 | 0.18 |
|  | NEE | 0.92 | 0.82 | 23.50 | 4.83 | 41.90 | 0.42 |
| FSM-E 2021, *Phragmites* 4 | Reco | 0.98 | 0.97 | −0.20 | 0.94 | 17.30 | 0.17 |
|  | GPP | 0.99 | 0.97 | −0.60 | 3.05 | 16.10 | 0.16 |
|  | NEE | 0.95 | 0.89 | 14.80 | 4.87 | 32.70 | 0.33 |
| FSM-E 2021, *Phragmites* 5 | Reco | 0.99 | 0.97 | −0.20 | 0.92 | 16.60 | 0.17 |
|  | GPP | 0.99 | 0.97 | −0.60 | 3.34 | 17.00 | 0.17 |
|  | NEE | 0.95 | 0.88 | 16.20 | 5.41 | 34.90 | 0.35 |
| FSM-E 2021, *Phragmites* 6 | Reco | 0.99 | 0.98 | −0.20 | 0.96 | 15.50 | 0.16 |
|  | GPP | 0.98 | 0.97 | −0.70 | 3.60 | 17.90 | 0.18 |
|  | NEE | 0.94 | 0.87 | 16.70 | 5.69 | 36.30 | 0.36 |
| FSM-E 2021, *Phragmites* 7 | Reco | 0.98 | 0.97 | −0.10 | 1.02 | 18.20 | 0.18 |
|  | GPP | 0.99 | 0.98 | −0.70 | 3.11 | 15.40 | 0.15 |
|  | NEE | 0.95 | 0.89 | 13.50 | 5.21 | 32.40 | 0.32 |
| FSM-E 2021, *Phragmites* 8 | Reco | 0.97 | 0.93 | −0.10 | 1.54 | 26.10 | 0.26 |
|  | GPP | 0.98 | 0.97 | −0.70 | 3.52 | 17.50 | 0.17 |
|  | NEE | 0.95 | 0.89 | 14.50 | 5.25 | 33.30 | 0.33 |
| FSM-E 2021, *Phragmites* 9 | Reco | 0.99 | 0.97 | −0.10 | 0.91 | 16.30 | 0.16 |
|  | GPP | 0.98 | 0.97 | −1.10 | 3.24 | 17.40 | 0.17 |
|  | NEE | 0.95 | 0.88 | 13.60 | 4.94 | 34.30 | 0.34 |
| FSM-E 2021, *Typha* 1 | Reco | 0.98 | 0.96 | −0.10 | 0.93 | 20.90 | 0.21 |
|  | GPP | 0.99 | 0.98 | −0.20 | 1.88 | 14.60 | 0.15 |
|  | NEE | 0.94 | 0.87 | 26.10 | 3.53 | 35.60 | 0.36 |
| FSM-E 2021, *Typha* 2 | Reco | 0.97 | 0.93 | −0.10 | 1.01 | 25.50 | 0.25 |
|  | GPP | 0.99 | 0.97 | 0.10 | 1.97 | 16.60 | 0.17 |
|  | NEE | 0.94 | 0.87 | 28.80 | 3.45 | 36.70 | 0.37 |
| FSM-E 2021, *Typha* 3 | Reco | 0.90 | 0.80 | 0.00 | 2.43 | 44.10 | 0.44 |
|  | GPP | 0.97 | 0.95 | −1.10 | 2.52 | 22.70 | 0.23 |
|  | NEE | 0.92 | 0.82 | 40.30 | 3.86 | 42.00 | 0.42 |
| FSM-E 2021, *Typha* 4 | Reco | 0.98 | 0.96 | −0.30 | 0.89 | 19.70 | 0.20 |
|  | GPP | 0.99 | 0.97 | 0.00 | 2.23 | 16.20 | 0.16 |
|  | NEE | 0.94 | 0.87 | 21.40 | 3.92 | 36.70 | 0.37 |
| FSM-E 2021, *Typha* 5 | Reco | 0.95 | 0.91 | −0.10 | 1.85 | 30.40 | 0.30 |
|  | GPP | 0.98 | 0.96 | −0.60 | 3.11 | 20.60 | 0.21 |
|  | NEE | 0.92 | 0.83 | 35.70 | 5.22 | 41.30 | 0.41 |
| FSM-E 2021, *Typha* 6 | Reco | 0.98 | 0.95 | −0.20 | 1.08 | 21.60 | 0.22 |
|  | GPP | 0.98 | 0.97 | −0.30 | 2.49 | 18.60 | 0.19 |
|  | NEE | 0.91 | 0.80 | 32.20 | 4.59 | 45.10 | 0.45 |
| FSM-E 2021, *Typha* 7 | Reco | 0.99 | 0.98 | −0.10 | 0.73 | 14.20 | 0.14 |
|  | GPP | 0.99 | 0.98 | −0.50 | 2.57 | 15.30 | 0.15 |
|  | NEE | 0.93 | 0.86 | 17.90 | 4.89 | 37.70 | 0.38 |
| FSM-E 2021, *Typha* 8 | Reco | 0.99 | 0.98 | −0.10 | 0.60 | 13.70 | 0.14 |
|  | GPP | 0.99 | 0.98 | −0.10 | 2.22 | 14.20 | 0.14 |
|  | NEE | 0.95 | 0.90 | 16.30 | 3.96 | 32.00 | 0.32 |
| FSM-E 2021, *Typha* 9 | Reco | 0.99 | 0.98 | −0.10 | 0.62 | 13.70 | 0.14 |
|  | GPP | 0.99 | 0.97 | −0.20 | 2.44 | 15.90 | 0.16 |
|  | NEE | 0.95 | 0.89 | 17.40 | 4.05 | 33.90 | 0.34 |

## References

Bockermann, C., Eickenscheidt, T., & Drösler, M. (2024). Adaptation of fen peatlands to climate change: Rewetting and management shift can reduce greenhouse gas emissions and offset climate warming effects. *Biogeochemistry*, 167(4), 563–588. <https://doi.org/10.1007/s10533-023-01113-z>

Bockermann, C., Eickenscheidt, T., & Drösler, M. (2025). Greenhouse gas mitigation potential of temperate fen paludicultures - Dataset [Data set]. Zenodo. <https://doi.org/10.5281/zenodo.16026838>

Beetz, S., Liebersbach, H., Glatzel, S., Jurasinski, G., Buczko, U., & Höper, H. (2013). Effects of land-use intensity on the full greenhouse gas balance in an Atlantic peat bog, *Biogeosciences*, 10, 1067–1082, <https://doi.org/10.5194/bg-10-1067-2013>

Drösler, M. (2005). Trace gas exchange and climatic relevance of bog ecosystems, southern Germany. PhD thesis, Universität München, Germany, 182 pp.

Eickenscheidt, T., Heinichen, J., & Drösler, M. (2015). The greenhouse gas balance of a drained fen peatland is mainly controlled by land-use rather than soil organic carbon content. *Biogeosciences*, *12*(17), 5161–5184. <https://doi.org/10.5194/bg-12-5161-2015>

Elsgaard, L., Gorres, C.-M., Hoffmann, C. C., Blicher-Mathiesen, G., Schelde, K., & Petersen, S. O. (2012). Net ecosystem exchange of CO2 and carbon balance for eight temperate organic soils under agricultural management, *Agr. Ecosyst. Environ.*, 162, 52–67.

Falge, E., Baldocchi, D., Olson, R., Anthoni, P., Aubinet, M., Bernhofer, C., Burba, G., Ceulemans, R., Clement, R., Dolman, H., Granier, A., Gross, P., Grünwald, T., Hollinger, D., Jensen, N.-O., Katul, G., Keronen, P., Kowalski, A., Lai, C. T., … Wofsy, S. (2001). Gap filling strategies for defensible annual sums of net ecosystem exchange. *Agric. For. Meteorol.*, 107(1), 43–69. <https://doi.org/10.1016/S0168-1923(00)00225-2>

Fuss, R., Hueppi, R., & Pedersen, A. R. (2020). *gasfluxes: Greenhouse Gas Flux Calculation from Chamber Measurements* (Computer software). R package version 0.4-4. <https://CRAN.R-project.org/package=gasfluxes>

Heller, S., Tiemeyer, B., Oehmke, W., Gatersleben, P., & Dettmann, U. (2025). Wetter, but not wet enough—Limited greenhouse gas mitigation effects of subsurface irrigation and blocked ditches in an intensively cultivated grassland on fen peat, *Agric. For. Meteorol.*, 362, 110367. <https://doi.org/10.1016/j.agrformet.2024.110367>

IPCC. (2014). *IPCC 2014, 2013 Supplement to the 2006 IPCC Guidelines for National Greenhouse Gas Inventories: Wetlands*, Hiraishi, T., Krug, T., Tanabe, K., Srivastava, N., Baasansuren, J., Fukuda, M. & Troxler, T.G. (eds)*.* Published: IPCC, Switzerland.

Jurasinski, G., Koebsch, F., Guenther, A., & Beetz, S. (2014). *flux: Flux rate calculation from dynamic closed chamber measurements* (Computer software). R package version 0.3-0.1. <https://CRAN.R-project.org/package=flux>

Livingston, G. P., & Hutchinson, G. L. (1995). Enclosure-based measurement of trace gas exchange: Application and sources of error. In *Biogenic Trace Gases: Measuring Emissions from Soil and Water* (pp. 14–50). Blackwell Science.

Lloyd, J., & Taylor, J. A. (1994). On the Temperature Dependence of Soil Respiration. *Funct. Ecol.*, *8*(3), 315–323. <https://doi.org/10.2307/2389824>

Moriasi, D. N, Arnold, J. G., Van Liew, M. W., Bingner, R. L., Harmel, R. D., & Veith, T. L. (2007). Model evaluation guidelines for systematic quantification of accuracy in watershed simulations, *Am. Soc. Agr. Biolo. Engin.*, 50, 885–900.

Nash, J. E. & Sutcliffe, J. V. (1970): River flow forecasting through conceptual models: Part 1. A discussion of principles, *J. Hydrology*, 10, 282–290.

Säurich, A., Tiemeyer, B., Dettmann, U., Fiedler, S., & Don, A. (2021). Substrate quality of drained organic soils—Implications for carbon dioxide fluxes. *J. Plant Nutr. Soil Sci.*, 184(5), 543–555. <https://doi.org/10.1002/jpln.202000475>

Tiemeyer, B., Heller, S., Oehmke, W., Gatersleben, P., Bräuer, M., & Dettmann, U. (2024). Effects of water management and grassland renewal on the greenhouse gas emissions from intensively used grassland on bog peat. *Agric. For. Meteorol.*, 345, 109858. <https://doi.org/10.1016/j.agrformet.2023.109858>

von Post, L. (1924). Das genetische System der organogenen Bildungen Schwedens. In *Memoires sur la nomenclature et la classification des sols* (pp. 287–304). International Committee of Soil Science, 4, No 22.
